# Identification and functional validation of kynureninases from oral bacteria

**DOI:** 10.1080/20002297.2025.2561213

**Published:** 2025-09-21

**Authors:** Pisit Charoenwongwatthana, Halah Ahmed, Alex Charlton, Mark D. Gidley, Vsevolod Telezhkin, Jamie Coulter, Chien-Yi Chang

**Affiliations:** a School of Dental Sciences, Faculty of Medical Sciences, Newcastle University, Newcastle Upon Tyne, UK; b Department of Oral Medicine and Periodontology, Faculty of Dentistry, Mahidol University, Bangkok, Thailand; c Faculty of Science, Agriculture & Engineering, Newcastle University, Newcastle upon Tyne, UK

**Keywords:** Kynureninase, anthranilic acid, kynurenine, *Pseudomonas*, *Burkholderia*, *Ralstonia*, *Stenotrophomonas*

## Abstract

**Background:**

The kynurenine (KYN) pathway produces key metabolites for immunoregulation and neuromodulation in humans, but its presence and activity in the oral microbiome are unclear. This study investigates the functionality of the key kynureninase (KynU), which catalyses kynurenine to anthranilic acid (AA), in oral bacteria.

**Methods:**

Bioinformatic analysis identified putative *kynU* genes in oral bacterial genomes, and structural similarity of the predicted proteins was evaluated using Template Modeling (TM)-score and Root Mean Square Deviation (RMSD) analyses. Selected *kynU* sequences were cloned into the pBAD-His A expression vector. Enzymatic activity was accessed by quantifying AA concentrations using liquid chromatography-mass spectrometry (LC-MS).

**Results:**

Among 71 species, seven oral bacteria were identified to possess the *kynU*. Structural analyses indicated KynU from four species may fold into functional enzymes. Three recombinant KynU from *Burkholderiacepacia*, *Ralstoniapickettii*, and *Stenotrophomonasmaltophilia* produced detectable levels of AA (21.27 ± 12.0 µM, 19.59 ± 8.6 µM, and 46.43 ± 36.8 µM, respectively), confirming functional KYN-to-AA conversion.

**Conclusions:**

This study demonstrates KynU activity in oral bacteria, revealing an unrecognised aspect of microbial metabolism with potential implications for host-microbe interactions. Further investigation is required to elucidate the biological significance of bacterial KYN metabolites and their role in oral diseases.

## Key messages

1. Bioinformatic and structural analyses suggest that the kynurenine (KYN) pathway is present in oral bacteria.
2. Oral bacteria such as *Burkholderia cepacia*, *Ralstonia pickettii*, and *Stenotrophomonas maltophilia* possess functional KynU enzymes that convert KYN to anthranilic acid.
3. This study experimentally confirms active KYN metabolism in oral bacteria using recombinant expression and LC-MS.

## Introduction

Tryptophan (Trp) is an essential amino acid in humans, obtained from dietary sources and primarily absorbed in the small intestine [1]. Beyond its role in protein synthesis, Trp serves as a precursor for a variety of crucial bioactive metabolites, including melatonin, serotonin, and kynurenine (KYN) [2]. Consequently, Trp metabolism and its metabolites are highly relevant to important biological processes such as neuronal signalling and immunomodulation [1]. Although Trp can be catabolised through several pathways that produces a wide range of metabolites, the predominant route is the KYN pathway, which accounts for approximately 95% of Trp catabolism [2]. The KYN pathway also requires a series of multistep enzymatic reactions that contributes to numerous metabolites. As this KYN pathway is the major catabolic route of Trp, its dominance highlights the biological importance of KYN metabolites in cellular regulation [2].

KYN metabolites exhibit diverse biological functions. Kynurenic acid (KYNA) acts as an antioxidant by scavenging reactive oxygen species, whereas 3-hydroxy-L-kynurenine (3-HK) and quinolinic acid (QA) contribute to oxidative stress [3]. Both anthranilic acid (AA) and KYN act as antioxidants or pro-oxidants, depending on cellular environments [3]. Furthermore, QA and KYNA exert distinct neuronal effects; QA promotes neuronal excitability, whereas KYNA is neuroprotective [2]. Picolinic acid (PIC) exhibits antimicrobial activity against several bacteria such as *Mycobacterium avium* complex and *Staphylococcus aureus* [4,5]. Therefore, the KYN pathway dysregulation is implicated in various conditions, including neurological disorders, inflammatory diseases, and autoimmune disorders [3]. Additionally, elevated KYN metabolite levels may play a role in periodontitis [6].

In humans, Trp is converted *via* the KYN pathway by the enzymatic activity of either tryptophan 2,3-dioxygenase (TDO, Enzyme Commission/EC 1.13.11.11) or indoleamine 2,3-dioxygenase (IDO1/IDO2, EC 1.13.11.52), forming *Nʹ*-formylkynurenine, which is converted to KYN by formamidase (EC 3.5.1.9). KYN is catabolised to KYNA by kynurenine aminotransferase (KATs, EC 2.6.1.7), to AA by kynureninase (KynU, EC 3.7.1.3), or to 3-HK by kynurenine monooxygenase (KMO, EC1.14.13.9). AA and 3-HK are subsequently catabolised to 3-hydroxyanthranilic acid, which gives rise to QA or PIC. QA is ultimately utilised for nicotinamide adenine dinucleotide (NAD^+^) biosynthesis, an essential cofactor used in numerous metabolic processes and energy production [2].

Some bacteria can express TDO or IDO, enabling them to catabolise Trp *via* KYN pathway [7]. However, the bacterial KYN pathway remains under-investigated and its roles in influencing human health are not fully understood. *Pseudomonas aeruginosa*, an opportunistic pathogen, is among the few bacteria with a well-characterised KYN pathway. TDO is expressed in *P. aeruginosa*, enabling the catabolism of Trp through the KYN pathway. *P. aeruginosa* also carries the *kynU* gene, encoding KynU which is a pyridoxal phosphate (PLP)-dependent enzyme hydrolysing KYN to AA, similar to the function in humans [8]. AA in *P. aeruginosa* is a precursor for producing *Pseudomonas* quinolone signal (PQS), a key quorum sensing signal molecule regulating bacterial virulence and population behaviours such as biofilm formation [8,9]. AA produced by *P. aeruginosa* also exhibits antibiofilm activity against other bacteria such as *Bacillus subtilis* and *Salmonella enterica* [10].

Certain gut bacterial phyla, including Firmicutes and Proteobacteria, are known to carry the *kynU* gene [11], and these are also common in the oral cavity [12]. However, gene presence does not confirm enzyme functionality. While the impact of bacterial involvement in host KYN metabolism remains unclear, it may alter downstream metabolite levels and affect host health. This study identified *kynU* genes in oral bacteria and confirmed their catalytic activity in converting KYN to AA, suggesting a potential role in modulating oral KYN metabolism.

## Materials and methods

### Identification of bacteria harbouring gene encoding KynU

The Basic Local Alignment Search Tool (BLAST, https://blast.ncbi.nlm.nih.gov/Blast.cgi) and Kyoto Encyclopaedia of Genes and Genomes (KEGG, https://www.genome.jp/kegg/pathway.html) were used to identify bacterial genes *tdo*, *ido*, and *kynU*, which encode the key enzymes in the KYN pathway. To compile a list of bacteria harbouring *tdo* and *ido*, the identification was performed using the EC number of TDO (EC 1.13.11.11) and IDO (EC 1.13.11.52) to retrieve all orthologues from the KEGG database, based on existing annotations (accessed . The expanded Human Oral Microbiome Database (eHOMD, https://www.homd.org) was employed to verify a list of oral bacteria obtained from the identified data. To identify the *kynU* from these oral bacteria, two approaches were conducted. Firstly, a BLAST search was performed using the *kynU* sequence from *P. aeruginosa* PAO1 (accession no. NC002516.2) as a query with an e-value cutoff ≤ 0.001. Secondly, *kynU* orthologues were directly retrieved from the KEGG database, using existing annotations.

### Protein alignment and structural comparison

Amino acid sequences were derived from all bacterial *tdo* and *ido* retrieved from the KEGG database and were used to align and construct phylogenetic trees with MEGA11 [13]. Protein sequence alignment of KynU from oral bacteria was conducted using SnapGene v7.2 (Dotmatics, Boston, MA, US). 3D protein structures of KynU were predicted using AlphaFold2 and AlphaFold DB (https://alphafold.ebi.ac.uk) [14,15]. Structural similarity was evaluated using template modelling (TM)-score and root mean square deviation (RMSD) analyses [16–18].

TM-score provides a quantitative assessment of structural similarity between proteins, based on the distances between aligned residues as a following equation [17,18]:

$$\mathrm{TM}-\mathrm{score}=\;\frac{1}{L}{\left[\sum_{i=1}^{L_{\mathrm{aligned}}}\frac{1}{1+d_{i}^{2}/d_{0}^{2}}\right]}_{\max},$$

where *L* is the length of target protein sequence, *L*_*aligned*_ is the number of aligned residues between two proteins, and *d*_*i*_ is the distance between paired residues of two aligned structures, and *d*_*0*_ is calculated as: $\left(\sqrt[{3}]{L-15}\right)-1.8$.

RMSD is a measurement between superimposed protein structures, calculated based on the distances between pairs of the equivalent atoms as a following equation [16]:

$$\mathrm{RMSD}=\;\sqrt{\frac{1}{n}\sum_{i=1}^{n}d_{i}^{2}}\;,$$

where *n* is the number of equivalent atoms, and *d*_*i*_ is the distance between equivalent atoms of two proteins.

The reference protein structures and predicted models in protein data bank (PDB) file format were analysed with TM-score and RMSD through TM-score on-line tool (https://zhanggroup.org/TM-score). KynU structures from human (*Homo sapiens*), mouse (*Mus musculus*), yeast (*Saccharomyces cerevisiae*), bacteria (*P. aeruginosa* and *P. fluorescens*) were employed as reference proteins for comparative analysis [19,20].

### Cloning of *kynU* gene into an expression vector

*kynU* genes from *Burkholderia cepacia*, *Cupriavidus gilardii*, *H. sapiens*, *P. aeruginosa*, *P. fluorescens*, *P. otitidis*, *Ralstonia pickettii*, and *Stenotrophomonas maltophilia* were synthesised, sequencing-verified, and cloned into the pBAD-His A vector (Thermo Fisher Scientific, Waltham, MA, US) *via XhoI* and *HindIII* sites. The selection of these bacterial genes was based on the identified list of oral bacteria harbouring *kynU*. Plasmids were purified and transformed into *E. coli* DH10B. Constructs were named kynUBC, kynUCG, kynUHS, kynUPA, kynUPF, kynUPO, kynURP, and kynUSM, respectively. Plasmid sequences are available in the Supplementary Table 1.

### Bacterial culture conditions

Recombinant *E. coli* was cultured overnight at 37 °C on Luria-Bertani (LB) agar with 100 µg⋅mL^−1^ ampicillin, then inoculated into LB broth with 100 µg⋅mL^−1^ ampicillin and grown overnight with shaking at 200 rpm for subsequent experiments.

### Protein extraction

Overnight cultures in LB broth were sub-cultured to OD600 0.06–0.08 and incubated at 37 °C for 2 h until OD600 reached 0.4–0.6. Cultures were split into induced (0.1% w/v arabinose) and uninduced groups, then incubated for 3 h at 37 °C. Cells were pelleted (4,000 × g, 10 min, 4 °C), washed twice with PBS, and resuspended in 120 µL spheroplasting buffer containing 2 µL of 25 × protease inhibitor and 500 U⋅mL^−1^ mutanolysin (Sigma Aldrich, Saint Louis, MO, US). After 15 min at 37 °C, cells were lysed with 50 µL of 0.1 mm glass beads using TissueLyser LT (QIAGEN, Hilden, Germany) at 50 Hz for 2 min. The supernatant containing total proteins was collected and stored at –80 °C.

### Protein gel electrophoresis and Western blot analysis

A total 5 µg of protein per sample was resolved on sodium dodecyl sulphate-polyacrylamide gel electrophoresis (SDS-PAGE) and transferred onto polyvinylidene difluoride (PVDF) membrane (GE Healthcare, Chalfont St Giles, UK) and incubated for 1 hr with 6×-His tag monoclonal antibody (HIS.H8) conjugated to horseradish peroxidase (Thermo Fisher Scientific). The antibody was diluted 1:500 in PBS containing 0.05% (v/v) Tween 20 (PBST) and 5% (w/v) skimmed milk. Following three washes with PBST buffer, protein bands were developed using Clarity™ Western ECL substrate (Bio-Rad, Hercules, CA, US).

### KynU functional characterisation

Overnight cultures in LB broth were pelleted (4,000 × g, 10 min, 4 °C), washed twice with PBS, resuspended, and sub-cultured into RPMI 1640 medium (Thermo Fisher Scientific) containing 100 µg⋅mL^−1^ ampicillin and 1% (w/v) glucose. The RPMI 1640 medium was used instead of LB medium because RPMI 1640 medium is a defined medium with a precisely known chemical composition, while LB medium is a complex medium with variable composition of metabolites that could influence the results of functional characterisation [21]. Cultures were initiated at OD600 0.06–0.08 and incubated at 37 °C for 2 h until reaching OD600 0.4–0.6. Expression was induced with 0.1% (w/v) arabinose for 3 h, then divided into two groups: with or without 1 mM KYN (Sigma Aldrich). Both groups received 70 µM PLP (Sigma Aldrich) and were incubated at 37 °C for 30 min before centrifugation at 14,000 × g to collect supernatants for extracellular metabolite analysis. Intracellular metabolites were extracted by adding 400 µL of 80% (v/v) acetonitrile with 0.2% (v/v) formic acid, vortexed, and incubated on ice for 10 min [22], followed by centrifugation at 14,000 × g for 10 min at 4 °C. Constructs kynUPA and pBAD24 served as positive and negative controls, respectively. All samples were analysed by liquid-chromatography mass spectrometry (LC-MS).

### Standard control preparation and LC-MS analysis

L-Kynurenine, kynurenic acid, anthranilic acid, quinolinic acid, and 2-picolinic acid (Sigma Aldrich) were used as standards. Metabolite analysis was performed based on previously published methods [23] by using 2 µL sample injections on a Xevo TQ-S Triple-Quadrupole Mass Spectrometer (Waters, Wilmslow, UK). Separation was performed on a 2.1 × 150 mm Acquity UPLC HSS T3 1.8 µm column at 50 °C with a 200 µL⋅min^−1^ flow rate. Analyte concentrations were determined from peak areas using calibration curves.

## Results

### Bacteria harbouring *kynU*

**Figure 1.**
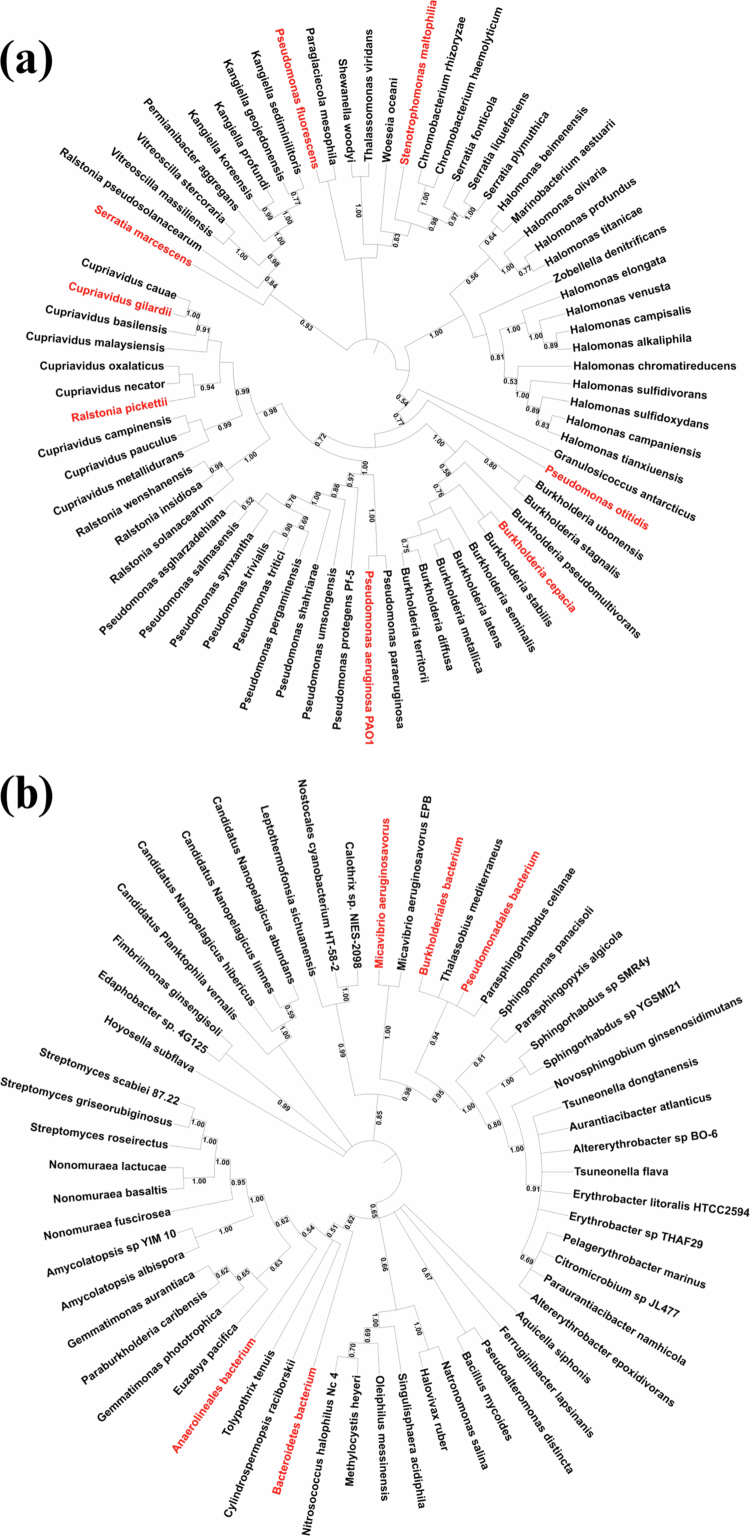
The phylogenetic trees constructed for bacteria harbouring *tdo* (a) and *ido* (b), respectively. A total of 71 bacterial species carried *tdo*, while 57 species carried *ido*. The bootstrap values associated with each internal node represent the level of confident support for the branching pattern. Higher values indicate greater confidence in the inferred evolutionary relationships within that clade. The bootstrap values less than 0.5 were not shown. The oral bacteria were highlighted in red.

Systematic database search from the KEGG database identified 71 species harbouring *tdo*, including eight oral bacteria listed in the eHOMD (Figure 1a), and 57 species harbouring *ido*, including five oral bacteria listed in the eHOMD (Figure 1b). A combination of BLAST searches and direct KEGG database retrieval verified seven oral bacteria harbouring both *tdo* and *kynU*, including *B. cepacia*, *C. gilardii*, *P. aeruginosa*, *P. fluorescens*, *P. otitidis*, *R. pickettii*, and *S. maltophilia*. None of the identified bacteria harboured both *ido* and *kynU*.

### KynU alignment and structural comparison

**Figure 2.**
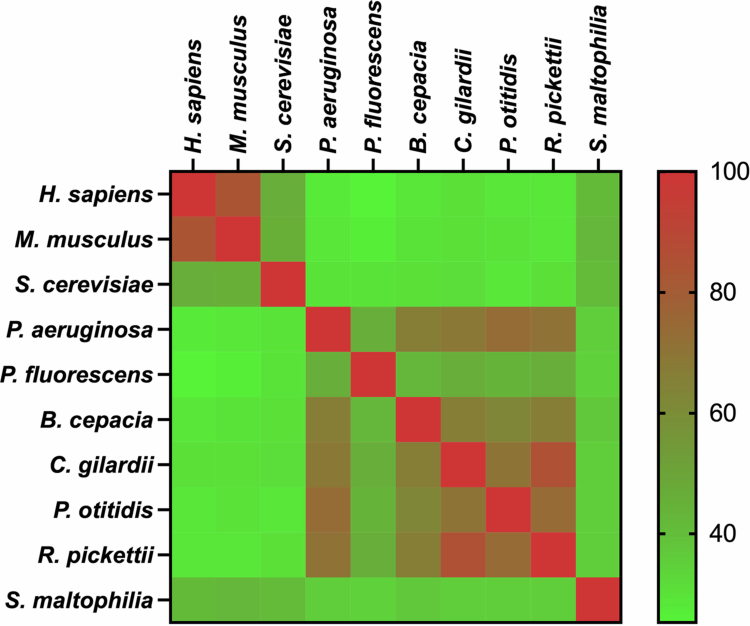
The amino acid sequences of KynU were subjected to compare sequence identity. KynU sequence from *S. maltophilia* shared approximately 40% identity with eukaryotic KynU sequences, while the other bacterial KynU showed around 30% identity. KynU sequences from *B. cepacia*, *C. gilardii*, *P. otitidis*, and *R. pickettii* displayed approximately 70% identity to *P. aeruginosa*.

KynU sequences from the identified oral bacteria were compared for sequence identity within the bacteria and against human, mouse, and yeast. Sequence alignment revealed that most of bacterial sequences shared approximately 30% identity with KynU sequences from *H. sapiens* (KynUHS), *M. musculus* (KynUMM), and *S. cerevisiae* (KynUSC). KynU sequence from *S. maltophilia* (KynUSM) exhibited approximately 40% identity with eukaryotic sequences and around 35% identity with the KynU from *P. aeruginosa* (KynUPA) and *P. fluorescens* (KynUPF). KynU sequences from *B. cepacia* (KynUBC), *C. gilardii* (KynUCG), *P. otitidis* (KynUPO), and *R. pickettii* (KynURP) exhibited approximately 70% identity with KynUPA (Figure 2). Sequence alignment and identity matrix are available in the Supplementary Figure 1 and Supplementary Table 2, respectively.

**Figure 3.**
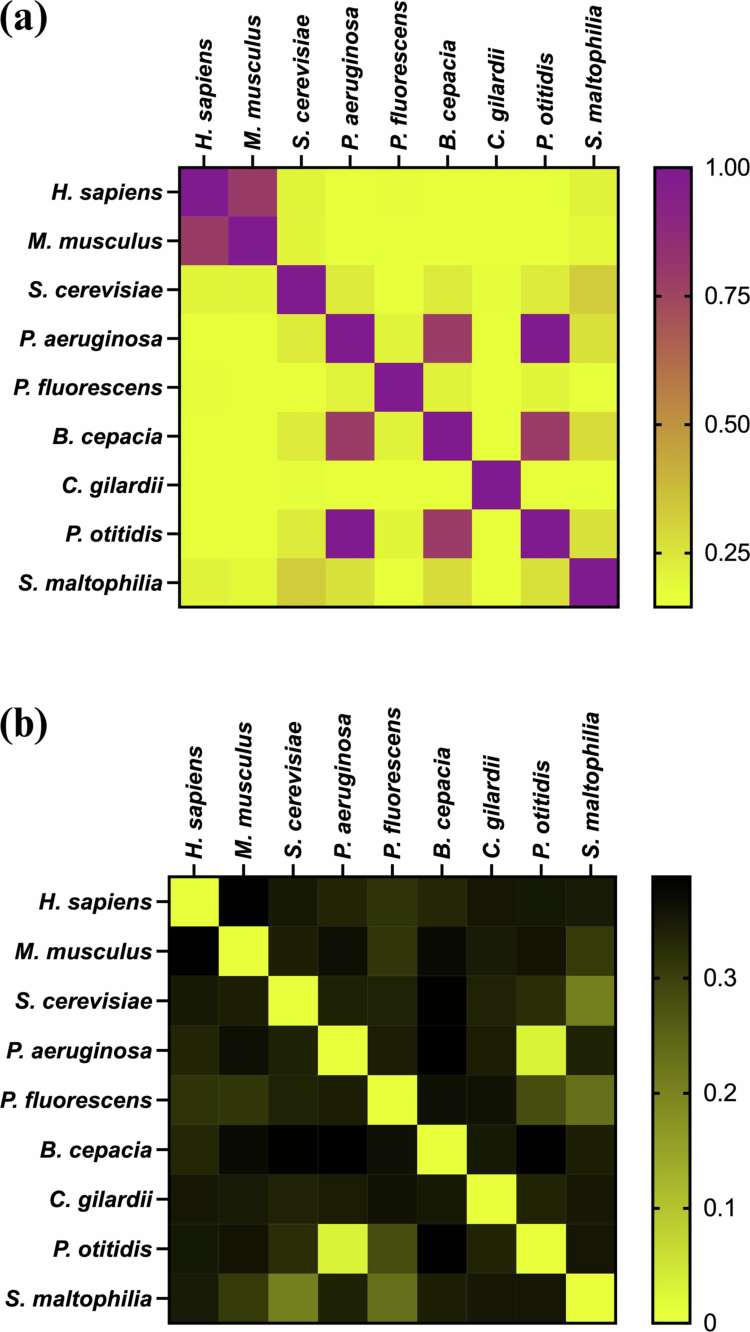
(a) A TM-score was employed to compare the predicted KynU protein structures from these identified bacteria to the reference structures. All predicted bacterial KynU structures showed TM-scores below 0.5 relative to eukaryotic KynU and KynUPF. KynUBC and KynUPO exhibited TM-scores above 0.5 relative to KynUPA. (b) The comparison between superimposed protein structures was assessed by RMSD. All predicted bacterial KYNU structures exhibited RMSD greater than 0.3 nm relative to KynUHS and KynUMM. KynUSM had RMSD approximately 0.2 nm relative to KynUSC and KynUPF. While KynUPO showed RMSD around 0.03 nm relative to KynUPA and 0.28 nm relative to KynUPF, both KynUBC and KynUCG displayed RMSD greater than 0.3 nm.

In addition to sequence identity results, structural comparison was performed based on TM-score and RMSD value, where a TM-score above 0.5 indicates two protein structures share overall similar fold and a RMSD value above 0.3 nm is considered a structural difference [17,24]. Bacterial KynU exhibited structural difference to eukaryotic KynU (TM-score < 0.5, RMSD > 0.3 nm), except KynUSM which showed some degrees of similarity to KynUSC (TM-score = 0.32, RMSD = 0.21 nm). KynUSM also showed partial similarity to KynUPF (TM-score = 0.17, RMSD = 0.23 nm). KynUPO exhibited similar folds with KynUPA (TM-score = 0.99, RMSD = 0.03 nm). KynUBC showed similar overall folds with KynUPA, though local structural differences were noted (TM-score = 0.78, RMSD = 0.39 nm). KynUCG was structurally dissimilar to both KynUPA (TM-score = 0.16, RMSD = 0.35 nm) and KynUPF (TM-score = 0.15, RMSD = 0.36 nm) (Figure 3), while structural analysis of KynURP was not applicable due to the absence of a fully predicted model; therefore, the structural similarity of KynURP cannot be determined. The KynU structures and comparison data are available in the Supplementary Figures 2 and 3, and Supplementary Table 3, respectively.

### KynU protein expression

**Figure 4.**
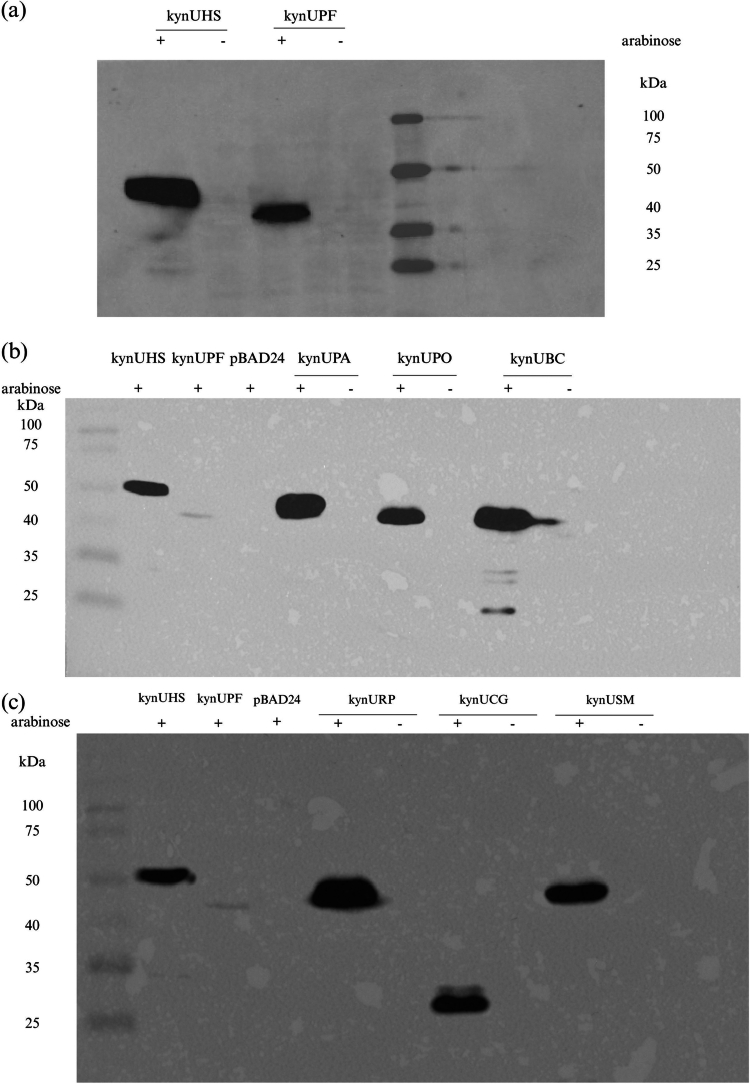
Immunoblots depict the protein expression of KynU in engineered bacteria. All constructs induced with 0.1% (w/v) arabinose showed protein bands on the immunoblot, including (a) KynUHS (56.2 kDa) and KynUPF (47.8 kDa), (b) KynUPA (47.14 kDa), KynUPO (46.63 kDa), and KynUBC (46.39 kDa), and (c) KynURP (46.75 kDa), KynUCG (28.01 kDa), and KynUSM (47.84 kDa). In contrast, no bands were observed in the pBAD24 vector control (b, c) and in constructs without arabinose induction. The plus sign (+) indicates samples induced with 0.1% (w/v) arabinose, while the minus sign (−) indicates uninduced controls.

Given structural similarities between oral bacterial KynU proteins and functional KynU enzymes from eukaryotes and *Pseudomonas*, candidate proteins were expressed and subjected to test for enzymatic activity. To verify the protein expression from recombinant *E. coli*, induction with 0.1% (w/v) arabinose was conducted, which successfully promoted the expression of KynU from the constructs: kynUHS (56.2 kDa), kynUPF (47.8 kDa), and kynUPA (47.14 kDa) and in the experimental group: kynUBC (46.39 kDa), kynUCG (28.01 kDa), kynUPO (46.63 kDa), kynURP (46.75 kDa), and kynUSM (47.84 kDa), as shown in the immunoblot (Figure 4). No protein bands were detected in pBAD24 and in the uninduced controls. These results provided qualitative confirmation that bacterial *kynU* genes in this study were not pseudo-genes, as their expression produced protein sizes corresponding to molecular weights of KynU sequence and His-tag (0.84 kDa).

### KynU enzymatic functional test

**Figure 5.**
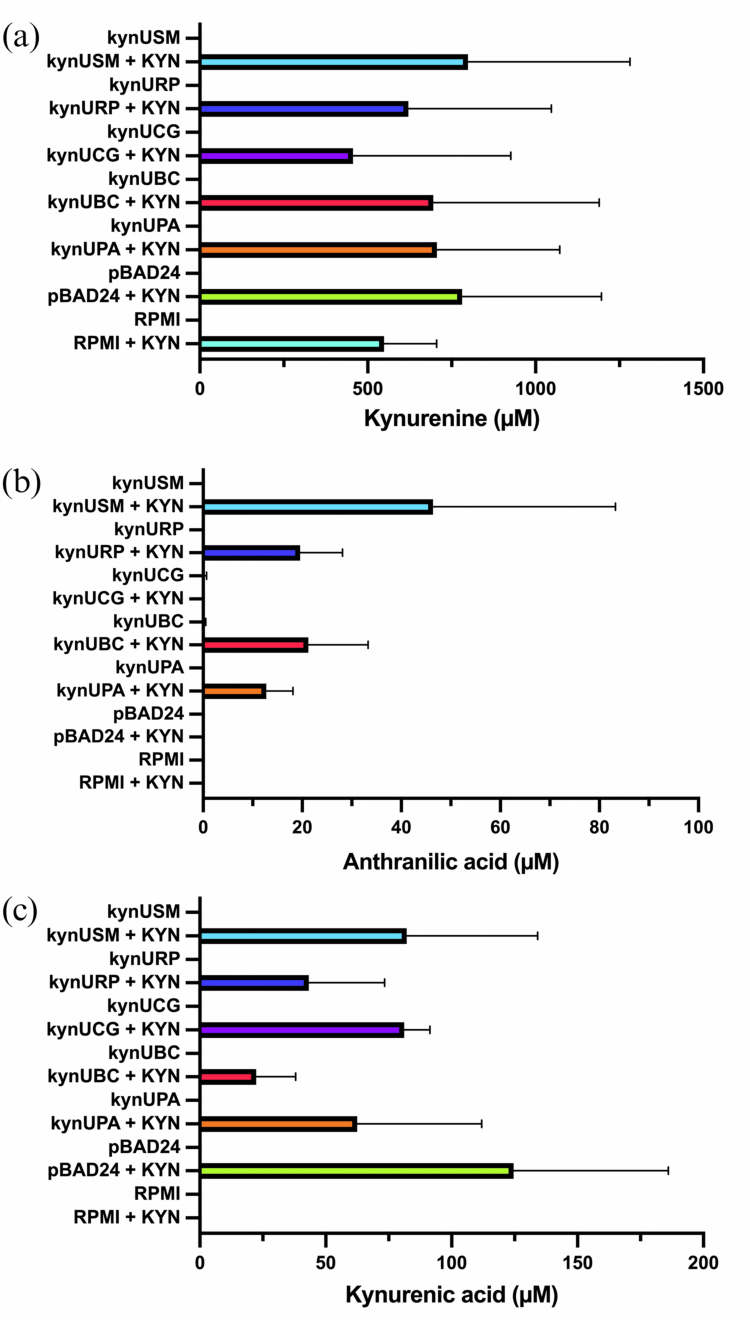
The bar graphs represent the total metabolites detected from functionality tests, including KYN, AA, and KYNA. (a) The results showed KYN detection from KynUBC (695.81 ± 493.5 µM), KynUCG (456.05 ± 470.3 µM), KynURP (621.05 ± 425.8 µM), KynSM (798.93 ± 482.0 µM), KynUPA (705.93 ± 365.9 µM), and pBAD24 (781.40 ± 414.8 µM), all of which were supplemented with KYN. (b) AA production was detected in the KYN-supplemented group for KynUBC (21.27 ± 12.0 µM), KynURP (19.59 ± 8.6 µM), KynUSM (46.43 ± 36.8 µM), and KynUPA (12.77 ± 5.4 µM). (c) Meanwhile, KYNA was detected in all bacterial experiments with KYN, including KynUBC (22.42 ± 15.6 µM), KynUCG (81.12 ± 10.3 µM), KynURP (43.33 ± 30.1 µM), KynUSM (82.21 ± 51.9 µM), KynUPA (62.49 ± 49.5 µM), and pBAD24 (124.59 ± 61.5 µM). The experiments were performed in three independent biological repeats.

Following confirmation of protein expression, KynU enzymatic function of catabolising KYN to AA was evaluated using LC-MS. KYN concentrations were measured to confirm its presence in samples. The total KYN was detected in the KYN-supplemented group: kynUBC (695.81 ± 493.5 µM), kynUCG (456.05 ± 470.3 µM), kynURP (621.05 ± 425.8 µM), kynUSM (798.93 ± 482.0 µM), kynUPA (705.93 ± 365.9 µM), and pBAD24 (781.40 ± 414.8 µM) (Figure 5a). The group without KYN, including RPMI medium alone showed KYN concentrations below the detection limit (< 0.1 µM).

The results showed detection of total AA in the KYN-supplemented group: kynUBC (21.27 ± 12.0 µM), kynURP (19.59 ± 8.6 µM), kynUSM (46.43 ± 36.8 µM), and kynUPA (12.77 ± 5.4 µM), which served as a positive control since KynU from *P. aeruginosa* is known to convert KYN into AA [8] (Figure 5b). Conversely, kynUCG and control conditions, including pBAD24, RPMI medium with or without KYN, and all non-KYK-supplemented groups, exhibited AA concentrations below the detection limit (< 0.001 µM).

To evaluate whether the oral bacterial KynU possesses dual functionality within the KYN pathway, the concentrations of KYNA, QA, and PIC were also measured. Total KYNA production was detected in the experimental group with KYN supplementation, including kynUBC (22.42 ± 15.6 µM), kynUCG (81.12 ± 10.3 µM), kynURP (43.33 ± 30.1 µM), kynUSM (82.21 ± 51.9 µM), kynUPA (62.49 ± 49.5 µM), and pBAD24 (124.59 ± 61.5 µM) (Figure 5c). RPMI medium with or without KYN, and the experimental group without KYN showed no detectable KYNA levels (< 0.1 µM).

QA and PIC were undetectable across all conditions. The constructs kynUHS and kynUPF failed to produce detectable AA levels in two biological repeats, while kynUPO showed inconsistent results ranging from undetectable to above-threshold levels. Therefore, these three constructs were excluded from the interpretation. The extracellular and intracellular ratios of KYN, AA, and KYNA showed high variability, with intracellular metabolites were undetected in one trial. Thus, the values reported here for each metabolite were the total concentrations. Raw data of metabolite concentrations are available in the Supplementary Table 4. The KynU functional test was performed in three independent biological repeats, and metabolite concentrations were reported as mean ± standard deviation.

## Discussion

KYN is a precursor for several biologically active metabolites in humans [2]. While some bacteria can convert Trp to KYN, the bacterial KYN pathway remains poorly understood [11]. This study shows that several oral bacteria, including *B. cepacia*, *R. pickettii*, and *S. maltophilia* possess KYN pathway genes and can produce AA from KYN *via* KynU.

Few bacteria have been identified possessing KynU, with the best-characterised examples are KynUPA and KynUPF [8,20]. Bioinformatic analysis revealed 7 oral bacteria harbouring *tdo* and *kynU*, all within the Proteobacteria phylum, consistent with prior study suggesting Proteobacteria theoretically possess the KYN pathway genes [11]. While Proteobacteria are abundant in the oral cavity, Burkholderiaceae, Pseudomonadaceae, and Xanthomonadaceae have relatively low prevalence in the human oral microbiome [12,25]. However, oral dysbiosis can lead to an increased abundance of these bacteria, which may be correlated with oral diseases such as periodontitis [26,27]. Accumulating evidence also supports their presence in oral niches, e.g.: *P. aeruginosa* has been isolated from subgingival plaque [28,29], and *Burkholderia* and *Ralstonia* have been detected in dental pulp samples [30]. Oral colonisation of *S. maltophilia* has been linked to prolonged antibiotic usage [31]. These findings underscore the importance of investigating these bacterial profiles, particularly their KYN pathways in relation to host-pathogen interactions.

Given the identification of these oral bacteria, this study systematically investigated KynU from identified oral bacteria through sequence identity, structural analysis, protein expression, and functional characterisation. Based on homology modelling, KynU sequences were aligned to assess similarity with eukaryotic KynU, KynUPA, and KynUPF, as higher sequence identity correlates with structural similarity [32]. Prokaryotic KynU sequences shared approximately 30% identity with eukaryotic KynU, suggesting an evolutionary relationship [32]. All analysed bacteria belong to Proteobacteria; thus, KynUBC, KynUCG, KynUPO, and KynURP sequences exhibited around 70% identity to KynUPA, but only 40% similarity to KynUPF. Conversely, KynUSM sequence shared approximately 35% identity with KynUPA and KynUPF. Nevertheless, similar protein structures can share identity as low as 10% [33,34].

TM-score and RMSD value analyses demonstrated that prokaryotic and eukaryotic KynU structures displayed less similarity. This structural divergence may reflect distant homologues and variation in protein evolution [33,35]. In comparison to KynUPA and KynUPF structures, KynUBC, KynUPO, and KynUSM displayed structural similarity with KynUPA and KynUPF, supporting the possibility of functional similarity. Nevertheless, protein expression was confirmed for the constructs kynUBC, kynUCG, kynUHS, kynUPA, kynUPF, kynUPO, kynURP, and kynUSM, which were used for downstream functional evaluation.

The functional assay was performed to evaluate whether predicted KynU could catalyse the conversion of KYN to AA, as established for KynUPA [8]. The KynUBC, KynURP, and KynUSM can produce AA using KYN as a substrate. These results were confirmed by detection of extracellular and intracellular AA from those constructs by LC-MS, suggesting AA was accumulated and also permeable through bacterial cells, which may influence the external environments. AA was undetectable in negative controls and in the absence of KYN, supporting the specificity of enzymatic activity.

KynU is a PLP-dependent enzyme that catalyses the hydrolysis of KYN to AA and 3-HK to 3-hydroxyanthranilic acid [2]. Eukaryotic KynU preferentially acts on 3-HK, whereas prokaryotic KynU favours KYN as a substrate [19,36]. KynUHS exhibits high affinity for 3-HK (Michaelis constant/*Km*~30 µM), but low affinity for KYN (*Km*~500 µM) [19], possibly explaining the lack of AA detection in two biological trials from kynUHS. Additionally, *E. coli* expression of eukaryotic proteins could be hindered by misfolding and lack of post-translational modifications, potentially impairing protein function [37].

The construct kynUCG consistently failed to produce AA in three biological trials. This lack of activity may be attributed to incompatibility with pBAD-His chassis or specific environmental requirements. Additionally, KynUCG showed structural dissimilarity to KynUPA and KynUPF, suggesting KynUCG may have a different function. For constructs kynUPF and kynUPO yielded inconclusive results in AA production from LC-MS analysis: kynUPF was excluded from further analysis as it is already well-characterised, while kynUPO exhibited fluctuating results that precluded definitive conclusions.

AA is an intermediate metabolite produced through KynU activity in humans and bacteria [36]. Clinically, increased AA concentrations have been associated with autoimmune disorders, such as rheumatoid arthritis and type 1 diabetes mellitus in humans [38,39]. While bacterial AA roles are less understood, it aids iron acquisition in *Rhizobium leguminosarum* [40], regulates pathogenicity genes in *Ralstonia solanacearum* [41], and is a precursor for PQS synthesis in *P. aeruginosa*, impacting virulence and immune evasion [8,9].

In humans, AA is also the source for QA production, which is known as a neurotoxic metabolite [2]. Investigating whether oral bacterial AA influences QA production from human cells is crucial to clarify if it can shift the balance of neuroactive KYN metabolites towards the neurotoxic QA branch, which is implicated in neurodegenerative diseases and neuropathic pain [42]. Emerging evidence demonstrates that oral microbiota can communicate with the brain *via* microbial translocation through trigeminal nerve and olfactory system, or indirectly though oral-gut microbiome interactions, which trigger a systemic immune response that compromises the blood–brain barrier, leading to neuroinflammation [43]. Therefore, the demonstration that oral bacteria can produce KYN metabolites highlights the significance of AA in host-pathogen interactions and provides a promising avenue for research on the impact of oral microbiome on oral-brain axis.

Beyond AA detection, KYNA was detected in all KYN-supplemented bacterial samples, whereas it remained undetectable in the medium regardless of KYN addition. These results suggest that KYNA may be produced by a putative protein in *E. coli*-based vector using KYN as a substrate. *E. coli* can express aspartate aminotransferase, which shares similar function to human KAT in catalysing the conversion of KYN to KYNA [44]. This finding may explain the presence of KYNA across all conditions containing KYN. KynU from selected bacteria was confirmed to have no function in QA and PIC production, as both metabolites were undetectable.

## Conclusion

This study demonstrates that oral bacteria, including *B. cepacia*, *R. pickettii*, and *S. maltophilia*, possess the KYN pathway and can catabolise KYN to AA through their functional KynU enzymes. Future directions include the investigation of the biological functions of AA related to oral health, and elucidating the roles of AA in bacteria and potential influence on the oral microbiome. Additionally, the biological importance of KYNA in bacteria requires further investigation, particularly in identifying and characterising the gene responsible for KYNA synthesis in oral bacteria.

## Acknowledgements

We express our gratitude to Ekaterina Kozhevnikova and Philip Hardy for their technical support. We thank the NIHR Newcastle Biomedical Research Centre (BRC) for the infrastructural support of CYC and JC research labs in the School of Dental Sciences at Newcastle University. This research in CYC’s laboratory is supported by the Rosetrees Trust (/100007). PC acknowledges the support of the Newcastle University Overseas Research Scholarship and the Faculty of Dentistry Mahidol University Scholarship for his PhD studentship. The presented research is those of the authors and not necessarily those of the NIHR or the Department of Health and Social Care. The funding agencies have no role in the preparation of this manuscript.

## Author contributions

PC contributed to conception and design, data acquisition, analysis, interpretation, and drafting of the manuscript. HA contributed to data acquisition, analysis, interpretation, and critical revision of the manuscript. AC contributed to analysis and interpretation. MG contributed to data interpretation and critical revision of the manuscript. VT contributed to interpretation and critical revision of the manuscript. JC contributed to conception and design, interpretation, and critical revision of the manuscript. C-YC contributed to conception and design, analysis, interpretation, and critical revision of the manuscript. All authors gave their final approval and agreed to be accountable for all aspects of the work.

## Supplementary Material

Supplementary material Kynureninase_Supplementary material

## Data Availability

The data underlying this article are available in the article and the supplementary material.
