# Supplementary material for: Identification and functional validation of kynureninases from oral bacteria
Source: J Oral Microbiol. 2025 Sep 21;17(1):2561213. doi: 10.1080/20002297.2025.2561213 (PMC12451956; doi:10.1080/20002297.2025.2561213)
Supplement: Supplementary material — Kynureninase_Supplementary material [file ZJOM_A_2561213_SM0187.docx]

**Supplementary Table 1: Plasmid sequences**

| Name | DNA sequence | NCBI accession number |
| --- | --- | --- |
| *kynU_BC_* | ATGATCAAGACCCGTGAAGACGCACTCGCGCTCGACCGCGACGATCCGCTCGCCCCGCTGCGCGACCAGTTCTCCCTGCCCGACGGCGTGATCTATCTCGACGGCAACTCGCTCGGCGCGCAGCCGCGCGCATCGGCCGCCCGCGCGCAGCAGGTGATCGGCGCCGAATGGGGCGAAGGCCTGATCCGCAGCTGGAACACGGCCGGCTGGTTCGCGTTGCCGCGCCGTCTCGGCGACAAGCTCGCGACGCTGATCGGCGGCGCACCGGGCGAAACGGTCGTCACCGACACCATCTCGATCAACCTGTTCAAGCTGCTGTCGGCGATGCTGCGCCACCAGGCCGAGCGCGCGCCCGAGCGCCGCGTGATCGTGTCGGAGCGCTCGAACTTCCCGACCGACCTGTATATCGCGCAGGGGCTGATCGAGCAGCTCGGCGGCAACTATGAACTGCGCCTGATCAACGATCCGGCCGACCTGCCGGCCGCGCTCGGCGCGGACACGGCCGTCGCGATGATCACGCACGTGAATTACCGCACCGGCTACATGCACGACATGCCGGCCGTCACGCAGCTCGTGCACGACGCGGGCGCGCTGATGCTGTGGGACCTCGCGCACTCGGCCGGCGCGGTGCCGGTCGACCTGAACGGCGCGCGCGCCGACGGCGCGGTCGGCTGCACGTACAAGTACCTGAACGGCGGCCCGGGTTCGCCCGCATTCGTGTGGGTGCCGCAGCGTCATCACGCACACTTCTCGCAGCCGCTGTCCGGCTGGTGGGGCCACCGCGCACCGTTCGCGATGCAGCCGGGCTTCGCGCCCGATCCGGGCATCGCGCGCTTCCTGTGCGGCACGCAGCCGATCGTGTCGATGTCGATGGTCGAATGCGGGCTCGACGTGTTCCTGCAAACCGACATGCAGGCGATCCGCCGCAAATCGCTCGCGCTGACCGATGCCTTCATCGCGCTCGTCGAAGCACGCTGCGCGGGCCTGTCGCTGAAGCTCGTCACGCCGCGCGCGCATCACCAGCGCGGCTCGCAGGCGAGCTTCGAGCATCCGCACGGCTACGAGGTGATGCAGGCGCTGATCGCGCGCGGCGTGATCGGCGACTACCGAGAGCCGTACGTGCTGCGCTTCGGCTTCACGCCGCTCTATGTGCGCTTCGTCGACGTGTGGGATGCCGTCGAGACGCTGCGCGACATCCTCGCCACCGACGCCTGGAAGGCGCCCGAGTTCGCCGAACGCGGCGCGGTGACCTGA | CP012981.1 |
| *kynU_CG_* | ATGCTGACCCACGTCAACTACAAGACCGGCGAGATGCTGGACATGGCGGCGCTGACCGAGCTGGCGCATGCGCGCGGCGCGCTGACGGTCTGGGATCTGGCGCATTCGGCGGGCGCGGTGCCGGTCGCGCTGCGCGAGTCCGGCGCCGACTACGCGGTCGGCTGCACCTACAAGTACCTGAACGGCGGCCCTGGTTCGCCAGCTTTCCTGTGGGTCGCGCCGGCGCTGCGCGACGCCTTCTGGCAACCGCTGTCGGGCTGGTGGGGCCACGCCGCGCCGTTCGCGATGGAGCCCGACTATCGCCCGCGCAGCGGCGTCTCGCGCTTCCTGTGCGGCACGCAGCCGATGACGTCGCTGGCGATGGTCGAATGCGGGCTGGAGGTCTTCGGCCGCACCGACATGCAGACGCTGCGCGCCAAGTCGCTGCAGCTGACCGACCTGTTCATCGCGCTGGTCGAAGCGCGCTGCGGCCATCATCCGCTGACGCTCGTCACGCCGCGCGAGCATGCGCGCCGCGGCAGCCAGGTCAGCTTCGCCCATCCGCAGGGCTATGCCGTGATGCAGGCGCTGATCGAGCGCGGCGTGATCGGCGACTACCGCGAGCCCCGGATCATCCGCTTCGGCTTCACGCCGCTCTACACTGGTTTTGCGGACGTCTGGGATGCGGTGGAGGTGCTGCGCGACGTGCTCGACAGCGGCGCCTACGAAGCGGCGCGCTTCCAGACGCGCACGGCGGTGACCTGA | CP010516.1 |
| *kynU_HS_* | ATGGAGCCTTCATCTCTTGAGCTGCCGGCTGACACAGTGCAGCGCATTGCGGCTGAACTCAAATGCCACCCAACGGATGAGAGGGTGGCTCTCCACCTAGATGAGGAAGATAAGCTGAGGCACTTCAGGGAGTGCTTTTATATTCCCAAAATACAGGATCTGCCTCCAGTTGATTTATCATTAGTGAATAAAGATGAAAATGCCATCTATTTCTTGGGAAATTCTCTTGGCCTTCAACCAAAAATGGTTAAAACATATCTTGAAGAAGAACTAGATAAGTGGGCCAAAATAGCAGCCTATGGTCATGAAGTGGGGAAGCGTCCTTGGATTACAGGAGATGAGAGTATTGTAGGCCTTATGAAGGACATTGTAGGAGCCAATGAGAAAGAAATAGCCCTAATGAATGCTTTGACTGTAAATTTACATCTTCTAATGTTATCATTTTTTAAGCCTACGCCAAAACGATATAAAATTCTTCTAGAAGCCAAAGCCTTCCCTTCTGATCATTATGCTATTGAGTCACAACTACAACTTCACGGACTTAACATTGAAGAAAGTATGCGGATGATAAAGCCAAGAGAGGGGGAAGAAACCTTAAGAATAGAGGATATCCTTGAAGTAATTGAGAAGGAAGGAGACTCAATTGCAGTGATCCTGTTCAGTGGGGTGCATTTTTACACTGGACAGCACTTTAATATTCCTGCCATCACAAAAGCTGGACAAGCGAAGGGTTGTTATGTTGGCTTTGATCTAGCACATGCAGTTGGAAATGTTGAACTCTACTTACATGACTGGGGAGTTGATTTTGCCTGCTGGTGTTCCTACAAGTATTTAAATGCAGGAGCAGGAGGAATTGCTGGTGCCTTCATTCATGAAAAGCATGCCCATACGATTAAACCTGCATTAGTGGGATGGTTTGGCCATGAACTCAGCACCAGATTTAAGATGGATAACAAACTGCAGTTAATCCCTGGGGTCTGTGGATTCCGAATTTCAAATCCTCCCATTTTGTTGGTCTGTTCCTTGCATGCTAGTTTAGAGATCTTTAAGCAAGCGACAATGAAGGCATTGCGGAAAAAATCTGTTTTGCTAACTGGCTATCTGGAATACCTGATCAAGCATAACTATGGCAAAGATAAAGCAGCAACCAAGAAACCAGTTGTGAACATAATTACTCCGTCTCATGTAGAGGAGCGGGGGTGCCAGCTAACAATAACATTTTCTGTTCCAAACAAAGATGTTTTCCAAGAACTAGAAAAAAGAGGAGTGGTTTGTGACAAGCGGAATCCAAATGGCATTCGAGTGGCTCCAGTTCCTCTCTATAATTCTTTCCATGATGTTTATAAATTTACCAATCTGCTCACTTCTATACTTGACTCTGCAGAAACAAAAAATTAG | NM_003937.3 |
| *kynU_PA_* | ATGACCACTCGTGACGACTGCCTGGCGCTCGACGCCGGCGATCCCCTGGCCGACCTGCGCCAACTGTTCGCCCTGCCCGACGGCGTGATCTACCTCGACGGCAACTCCCTCGGCGCCCGCCCCCGGGCCGCCGTGGAGCGCGCCGCCGAAGTGGTCGCCGCGGAATGGGGCGAGGGGCTCATCCGCAGCTGGAACAGCGCCGACTGGCGCGGCCTGCCGGAGCGCCTCGGCGACAAGCTGGCGCCCCTGATCGGCGCGCGCGCCGGCGAAGTGGTGATCACCGACACCACATCGATCAACCTGTTCAAGGTGCTCAGCGCCGCCCTACGGATCCAGGAGGAAGACGCGCCAGGGCGCAAGGTGATCGTTTCCGAATCGAGCAACTTCCCCACTGATCTGTACATCGCCGAGGGCCTTACCGACATGCTCCAGCGCGGCTACCGGCTACGCCTGGTAGATGACCCGGAGCAATTGCCGGCGGCGATCGACGCGGATACCGCGGTGGTGATGCTCAGCCATGTCAACTACAAGACCGGCTACCTGCACGACATGCGCGAGGTCACCCGCCTGGTCCACGAAAACGGCGCCCTGGCGATCTGGGACCTGGCCCACTCGGCCGGCGCGCTGCCGCTGGACCTGCACGCGGCCGACGCCGACTACGCCATCGGCTGCACCTACAAGTACCTCAACGGCGGCCCCGGCTCGCCGGCCTACGTGTGGGTCGCGCCGCGCCTGCGCGAGCGTGTCTGGCAACCGCTGTCCGGCTGGTTCGGTCATTCCCGCCAGTTCGCCATGGAGCCGCGCTACCAGCCCGGCGAAGGCATCACCCGGTTCCTCTGCGGCACCCAGCCGATCACCTCGCTGGCCCTGGTCGAATGCGGCCTGGATATCTTCGCCCGGACCGACATGCAGCGCCTGCGCGACAAGTCGCTGGCCCTCGCCGATCTGTTCATCGAATTGGTGGAAAGCCGTTGCGAACGCTTCGGCCTGACCCTGGTGACCCCGCGCGAGCATGCCCGGCGCGGCAGCCATGTCAGCTTCGAGCATGCCCAGGGCTACGCCATCGTCCAGGCCCTGATCGACCGGGGGGTAATCGGCGACTACCGCGAGCCGGGCATCCTGCGCTTCGGCTTCACCCCGCTTTACACCCGCTTCGTCGAGGTCTGGGATGCGGTGCAGGCGTTGCTGGAAATCCTCCAGAGCGAAGCCTGGAAAGAGCCGCGCTACCAGGTCCGGCACAAGGTGACCTGA | NC_002516.2 |
| *kynU_PF_* | ATGAACAAATTCGAACAAACACGCGCACGCTTTCATGTGCCCGAAGGCGTGCGTTACTTTGACGGCAACTCCCTGGGTTTGATGCCTGGCGCGGTGGCATCCCACGTCAACGAAGTGGTGACAAAACAGTGGGCCCAGGGGCTGATTCGGTCGTGGCACGAGGCCCAATGGCGCGTGTTGCCACAGACGGTCGGGCAAAAGGTCGCGCGCCTGATCGGTGCCCAGCCCGCCCACGTCGTGGCGTGCGACTCGACTTCGGCCAACCTGTTTAAAGTGCTGGTCAGTGCCGTGCGGCTGCGCCCTGGTCGCAACACGATTGTCACCGACGCCGACGCGTTCCCCACCGACCTGTACATCGTCTACCAGGTGGCCAGGCTGTTTGACCTCAAGGTGGTTGCTGTCCCGGCGGCGCAGATTGCGTCGCATCTGGATGAACAGGTGGCAACCGTAGTCTTGACCCATGTCGATTACCGCAGCGCCGAGATCTATGACATGGGCCAATACACCCGGCAGGCCCACGGGGTCGGTGCGCTGATTGTCTGGGATCTGTCCCATACCGCCGGCGGTGTTCCCTGCAACCTGGAGGCGGACAATGTCGACTTCGCAGTGGGATGTGGCTACAAGTACCTCAATGGCGGCCCGGGCGCCCCCGCCTATCTTTACGCTGCAGCCAGGCATCTGGACAAGTGCGAGCAACCCCTTGCCGGCTGGTTCGGGCACGCACGCCCGTTCGATTTCAGCCAGGACTACACGCCGGCTGAAGGTGTGTCGCGGTTCCTCTGCGGTACCAACCCGGTCATCGGGCTGTCGGTCCTTTCCAGGGCGCTCGATGAGTTCGATGGTGTTGATATGCAGGAGATCCGCGCGCGTAGCGTGGTCCTGACCGGGCAATTCATCGACGGTGTCGACACCGAGCTCGTCGGGCTTGGTTTTCACCTCGCCTCGCCCCGCGAGGCCGCAAAGCGCGGCAGCCATGTTTCGCTGCGACACCAACAGGGCTACGGGATCATGCAGGAATTGGCCTTGCGCGGTTACATCGGTGACTTCCGCACACCCGACTACATGCGCTTTGGTTTCGCCCCCTTGTACAACAATGAACAGGATGTGTGCGAACTGCTCAGCGAAATCAAACTGATCATGGACACCCGCGCCTGGGATAAACCGGCCTATCAGCAGCGCCGCGACTTTACCTAG | CP008896.1 |
| *kynU_PO_* | ATGACAACAAGACAAGCCTGCCTTGACCTCGACGCCCGCGATGCCCTCGCGCCCCTGCGCGACGCCTTCGCCCTGCCCGAAGGGGTCATCTACCTCGACGGCAACTCCCTCGGCGCGCGCCCCAAGGCCGCCCTCGAACGGGCCCGGCAAGTGGTAGAGGACGAATGGGGCACCGGCCTGATCGGCAGCTGGAACGAAGCCGGCTGGCGCGGCCTGCCCGAGCGCCTGGGCAACCAGCTCGCCAGCCTCATCGGCGCCGGCCAGGACGAAGTGGTGATCACCGACACCACCTCCATCAACCTCTTCAAGGTGCTGGTCGCCGCCCTGCGCGTGCAGGCCCAGCGCGACCCCGCTCGCAAGGTCATCGTCACCGAGAGCAGCAACTTCCCCACCGACATCTACATGGTCGAAGGCCTCGCCGACCTGCTCCAGCAGGGCTACAGCCTGCGCCTGGTGGACACCCCCGAGGAGCTGCCCGCCGTCATCGGCACCGACACCGCCGTCGCCCTCATCACCCAGGTCAACTACAAGACCGGCTACCTGCACGACATGCAGGCCCTCACCGCCCTGGCCCACGAATGCGGCGCGCTAACCATCTGGGACCTCTGCCACTCCGCCGGCGCCGTGCCCATCGACCTCACCGCCGCCAAGGCCGACTACGCCATCGGCTGCACCTACAAGTACCTCAACGGCGGCCCCGGCTCCCCGGCCTTCGTCTGGGTCGCCCCCGCCCTGCGCGAGCTGGTCTGGCAACCGCTGTCCGGCTGGTGGGGCCACGCCCGCCAGTTCGGCATGGAGCCCGCCTACGAGCCGGCACCCGGCATCGGCCGCTACCTCTGCGGCACCCAGCCGATCACTTCCCTGGCCATGATCGAGTGCGGCCTCGACGTCTACGCCCGCACCAGCATGGAGGCCCTGCGGGAGAAGTCCCTGGCCCTCACCGACCTCTTCATCGAGCGCGTGCAGGCCCGCTGCGGCCAGCACCCGCTCACCCTGGTCACCCCCCTGGAGCACGCCCGCCGTGGCAGCCACGTCAGCTACGAGCACCCCGAGGGCTACGCCATCATCCAGGCGCTGATCGACCGGGGCGTGGTCGGCGACTACCGCGAGCCGCGCATCATGCGCTTCGGCTTCACCCCCCTCTACACCCGCTTTGTCGACGTCTGGGACGCCGCCGAAACCCTCGGCGAGATCCTCGACAGCGAGGCCTGGCGCCAGGAGCGCTTCATGGCCCGCAAGCAGGTGACCTGA | AP022642.1 |
| *kynU_RP_* | ATGACGACAACTGACCGCGAGCAATGTATCCGGCTCGATCAGCAAGACCCGCTGCGCCCGCTGCGCGACCAGTTCGCACTGCCCGAGAGCGTGATCTACCTCGACGGCAATTCGCTCGGGGCCCGCCCGCGCGCCGCTGCCGCGCGCGCCGCCCAGGTGGTGGCCGAGGAATGGGGCGAAGGCCTGATCCGCAGCTGGAATACCGCCGGCTGGTTCGAACTGCCGCAGCGGCTGGGCAACAAGCTCGCCCCGCTGGTGGGCGCCGGCCAGGACGAAGTGGTGGTGACCGACACCACCTCGATCAACCTGTTCAAGGTACTGGCCGCGGCGCTGCGGGTGCAGCAGACGCGCGATCCTGCGCGCAAGGTGATCGTGTCCGAGGCCAGCAACTTCCCGACCGACCTCTATATCGCGCAGGGCCTGGCCGACCTGCTGCAGCAGGGCTATTCGCTGCGCCTGGTCAATTCGCCGGCCGAGCTCGACGCCGCGGTCGGCGCCGACACCGCCGTGCTGATGCTCACGCACGTCAACTACAAGACCGGCGAAATGCTCGACATGGCCTCGCTGACCGAGCTCGCCCACGCACGCGGCGCGCTGACGGTGTGGGACCTGTGCCACTCCGCCGGCGCGGTGCCGGTCAACCTGAAGGCGGCCGGCGCCGACTACGCCATCGGCTGCACCTACAAATACCTGAACGGCGGCCCGGGCTCGCCGGCCTTCGTCTGGGTCGCTCCCGCGCTGCGCGATGCCTTCTGGCAGCCGCTGTCGGGCTGGTGGGGCCACGCCGCGCCGTTCGCGATGGAGCCGCAATACCGCCCGGTCGACGGCGTGCGCCGCTTCCTGTGCGGCACGCAGCCGGTGACTTCGCTGGCGATGGTCGAGTGCGGCCTGGACATCTTCGCCCAGACCAATATGCAGGTGCTGCGCGCCAAGTCCCTGCTGCTGACCGACTTGTTCATCGAACTGGTGGAGGCACGCTGCGGCCACCACCCGCTGACGCTGGTCACGCCGCGCGAGCACGCGCGCCGCGGCAGCCAGGTCAGCCTGGAGCATCCGGAAGGCTATGCGCTGGTGCAGGCGCTGATCGAGCGCGGCGTGATCGGCGATTACCGCGAGCCGCGCATCGCCCGCTTCGGTTTCACGCCGCTGTACACCAGCTTCACCGAGGTGTGGGATGCTGTGGAAATCCTGCGCGATGTACTGGACAGCGTCGCCTATCGCGACGCGCGCTTCCAGACGCGCGGCCAGGTGACCTGA | CP006667.1 |
| *kynU_SM_* | ATGTCCGACCTGCTCAGCCGCACCCACGCCATCGCCTTGGACGCCGCCGATCCGCTGCGCCCGCTGCGCAGTGAATTCCTGATTCCGCGCCATGAGGGCGGCGAGCAGACCTACTTCGTAGGCAACTCGCTGGGCCTGCAGCCGCGCGGCGCGCAGGCAGCGGTGCAGGAAGTGATGAAGCAGTGGGGCGAACTGGCGGTGGAAGGCCACTTCACCGGCCCGACGCAGTGGCTGTCCTACCACCGCCTGGTGAGCGCGCAGCTGGCCCGCGTGGTTGGCGCGCTGCCCAGCGAAGTGGTGGCGATGAACACGCTGAGCGTAAACCTGCACCTGATGATGGTCAGCTTCTACCGGCCGACCACGCAGCGCCCGGTGATCCTGATGGAAGCCGGCGCGTTCCCGACCGATCGCCACGCGGTGGAAGCCCAGATCCGCTTCCATGGCTTCGACCCGGCCGAGTGCCTGGTGGAAGTGCAGCCGGACGAAGCCAACGGCACGATTTCACTGAACGCCATCGAGCGCGCCATGACCGAACACGGCCCGCGCCTGGCGCTGGTGCTGTGGCCTGGCGTGCAGTACCGCACCGGCCAGGCCTTCGACCTCGATGCGATCACCCGCGCCGCCCGCCTGCAGGGCGCACGCATCGGCTTCGACCTGGCGCACTCGGTCGGCAACCTGCCGCTGCGCCTGCATGACGTGGCCCCCGATTTCGCCGTGTGGTGCCATTACAAGTACCTCAACAGCGGCCCGGGCGCAGTGGCTGGCGCCTTCGTGCACGAGCGCCACCACCGCGATACCACCCTGCCGCGCTTTGCTGGCTGGTGGGGCCATGAGGAAGCCACCCGCTTCCAGATGGCGCCGCAGTTCACCCCGGCCATCGGCGCCGAGGGTTGGCAGCTGAGCAATCCACCGATCCTCGGCCTGGCACCGCTGCGCGCCTCGCTGGACCTGTTCGAGCGCGCCGGCATGGAGGCGCTGCGCAGCAAATCGCTGGCGCTCACCGGCATGCTCGAAGCACTGGTGCGCGCACGCCTGCCGCAGGTGCTGGACATCATCACCCCGGCCGATCCGCAGCGCCGCGGCTGCCAGTTGTCGCTGCGCGTGATCGGTGGCCGCGAGCGTGGCCGCGCACTGTTCGAGCACCTGCGCGGCATCGGTGTGCTCGGCGACTGGCGCGAACCCGACGTGATCCGCATCTCGCCCACCCCGCTCTACAACCGCTACCTGGACGTGCACCACTTCGTCGAGGAAGTGGAAGCCTGGGCCGGCCTTTGA | HE798556.1 |

**Supplementary Figure 1: Amino acid sequence alignment for KynU**


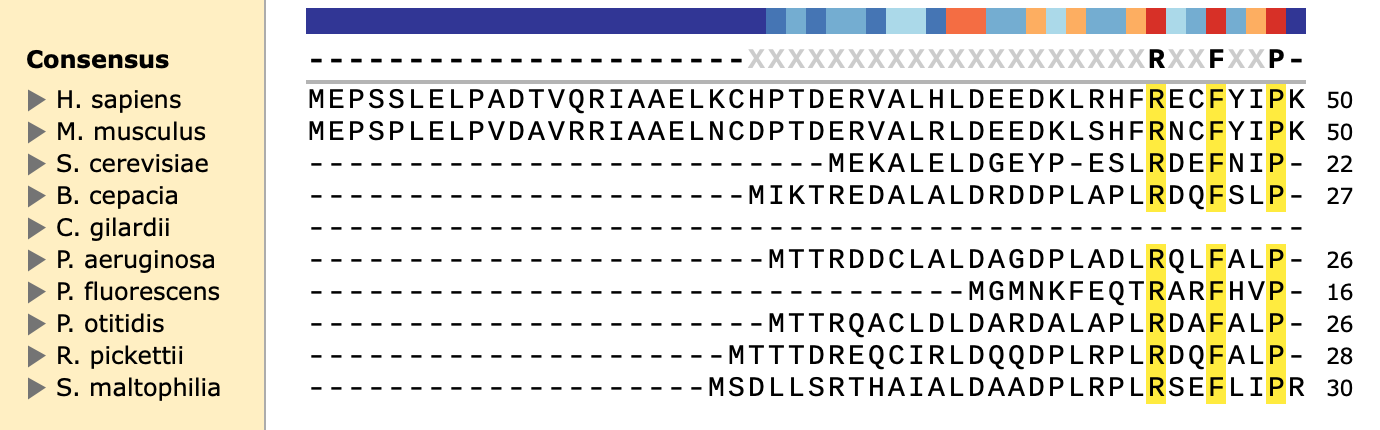


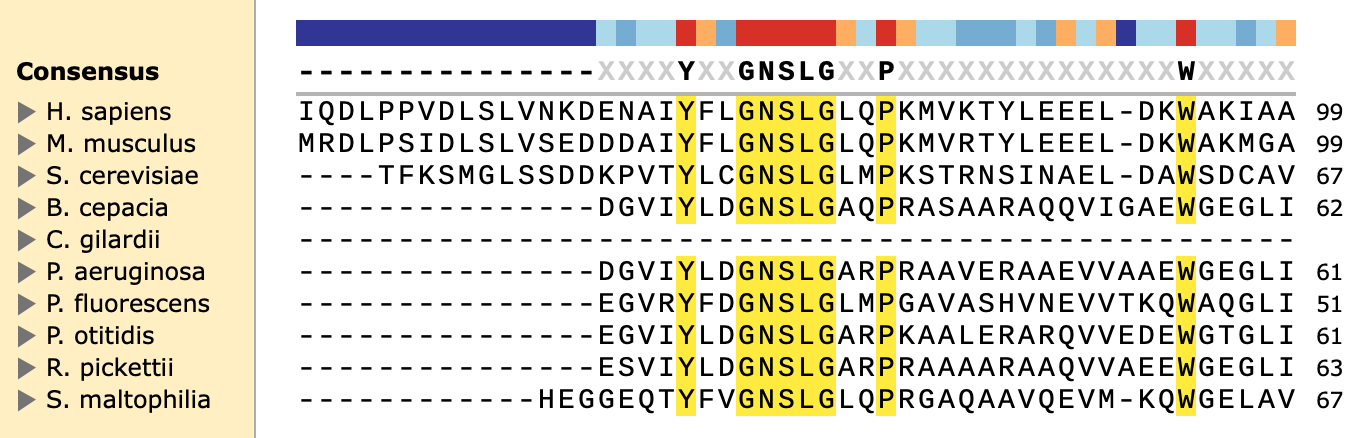


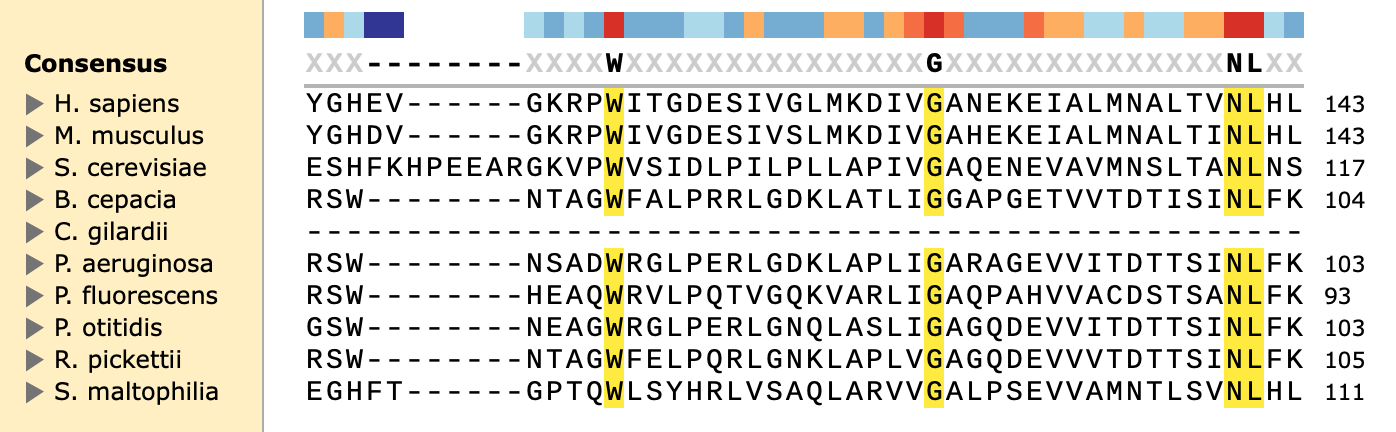


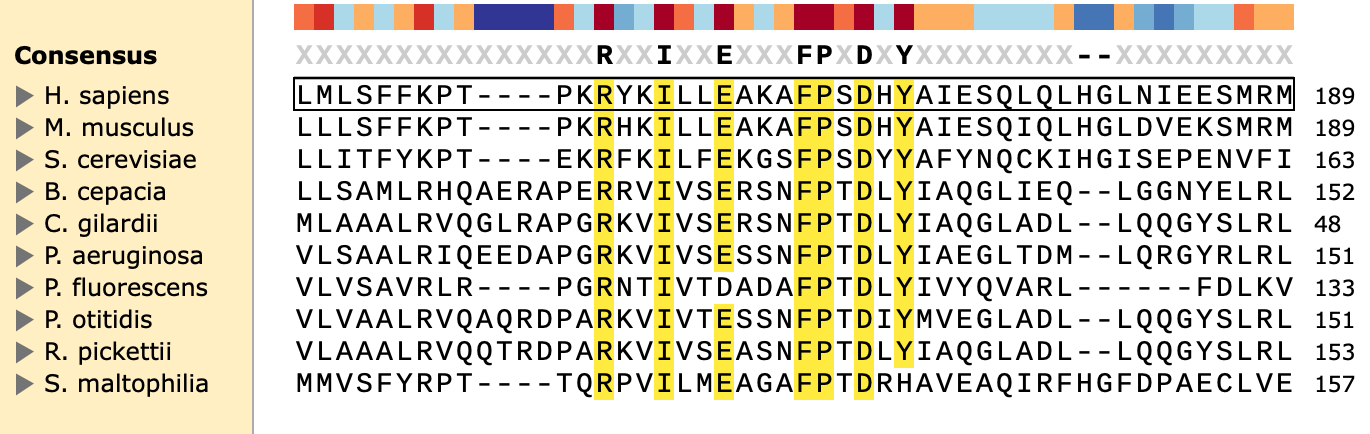


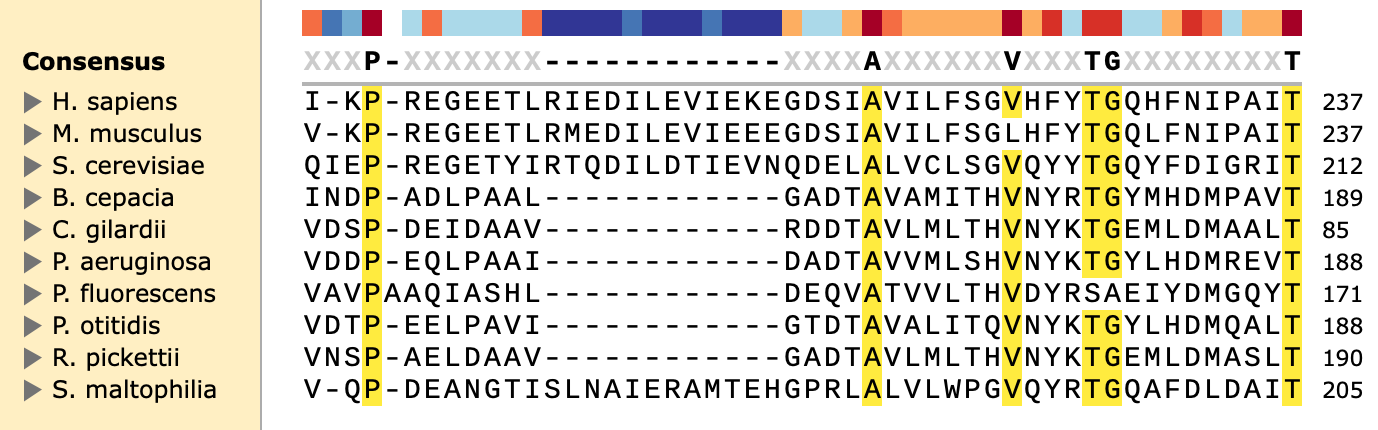


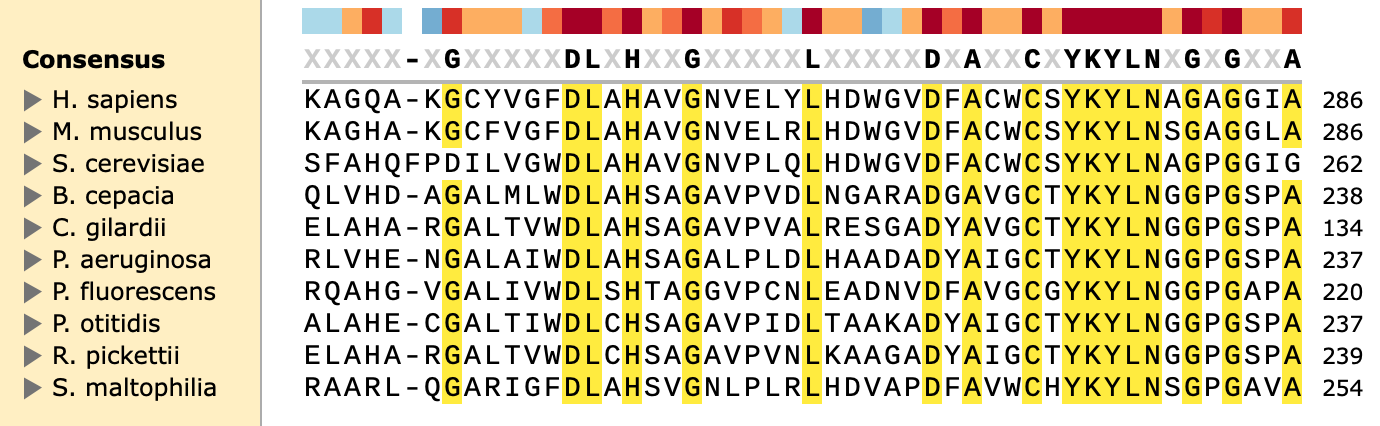


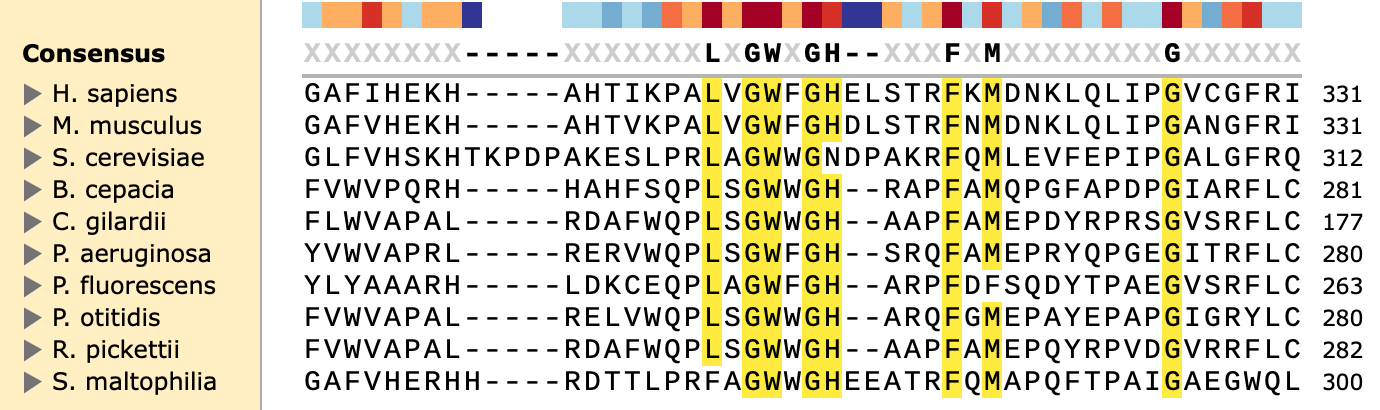


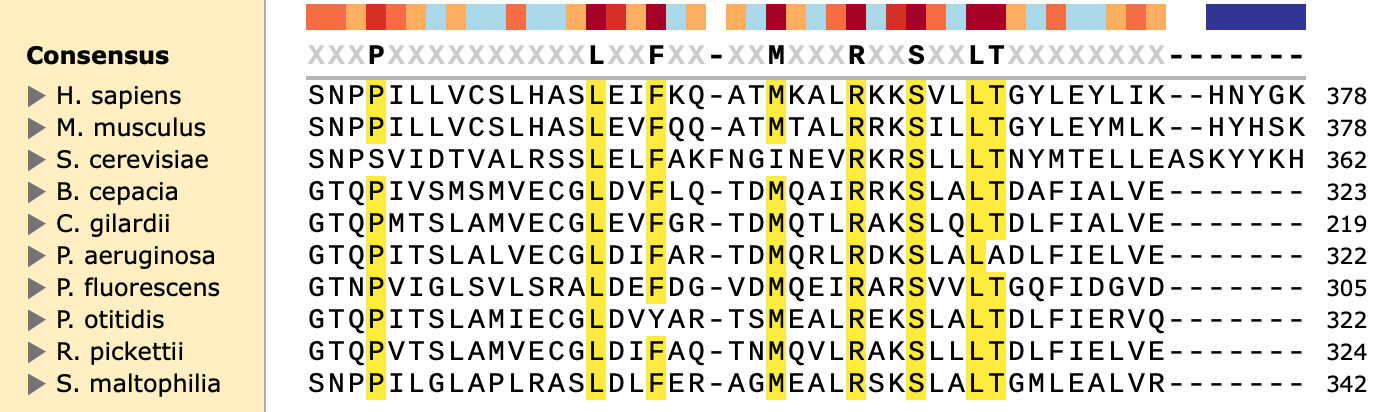


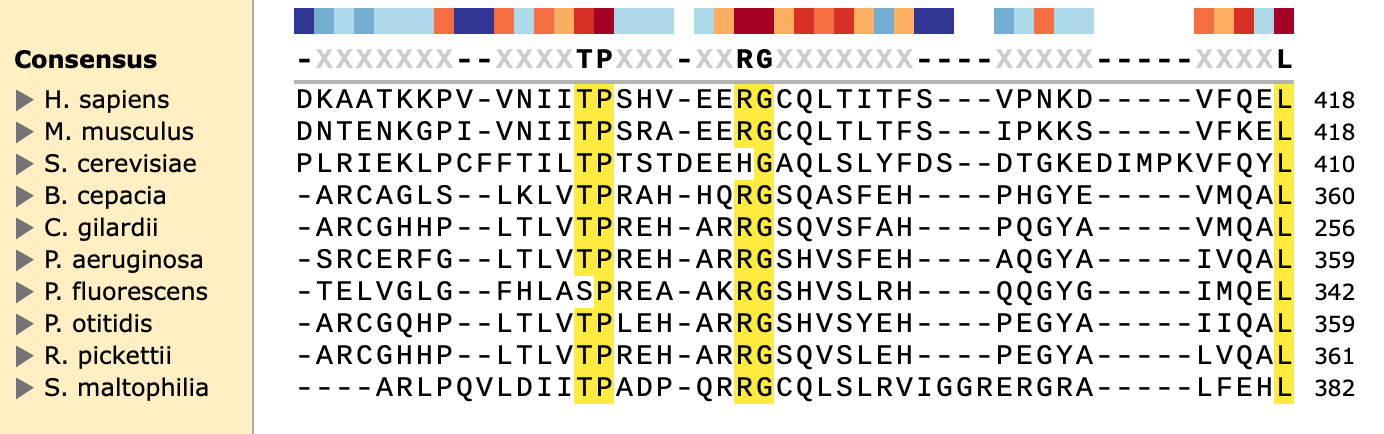


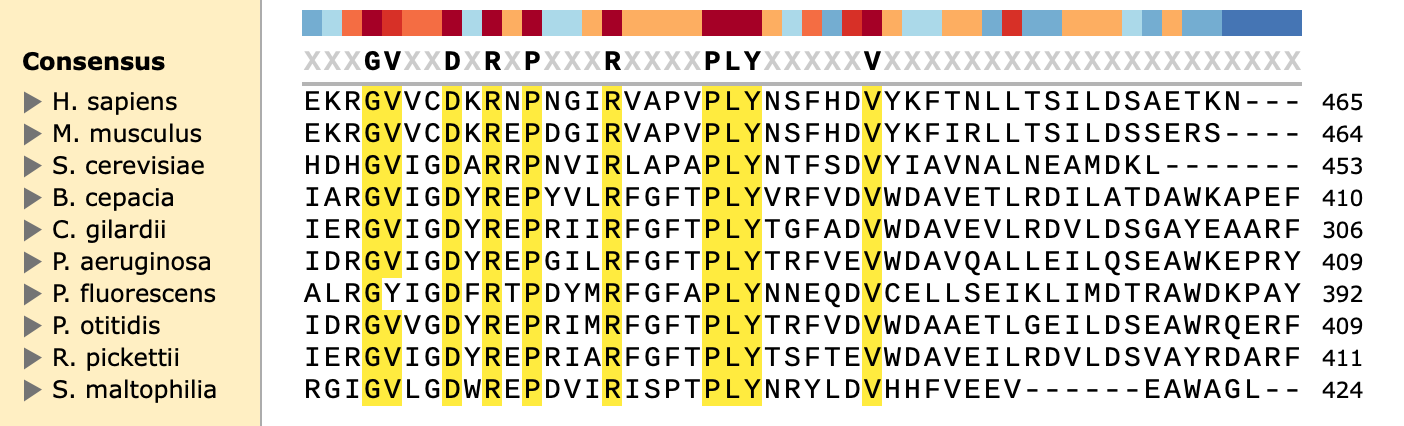


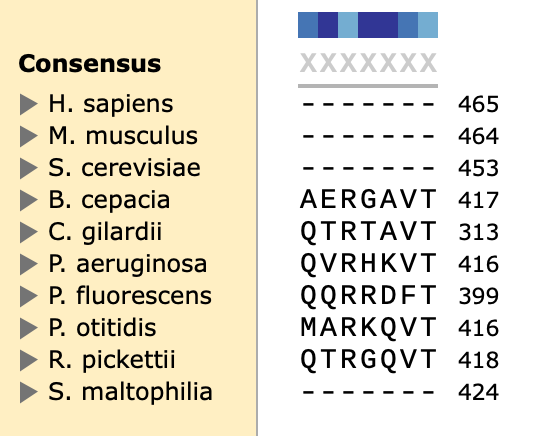


**Supplementary Table 2: Sequence identity matrix**

|  | **H. sapiens** | **M. musculus** | **S. cerevisiae** | **P. aeruginosa** | **P. fluorescens** | **B. cepacia** | **C. gilardii** | **P. otitidis** | **R. pickettii** | **S. maltophilia** |
| --- | --- | --- | --- | --- | --- | --- | --- | --- | --- | --- |
| **H. sapiens** | 100.00 | 83.41 | 45.92 | 27.43 | 25.26 | 28.36 | 30.10 | 28.43 | 28.54 | 41.19 |
| **M. musculus** | 83.41 | 100.00 | 45.45 | 28.25 | 26.36 | 29.18 | 30.54 | 30.00 | 28.86 | 42.14 |
| **S. cerevisiae** | 45.92 | 45.45 | 100.00 | 29.26 | 29.24 | 30.28 | 30.85 | 29.01 | 30.53 | 40.77 |
| **P. aeruginosa** | 27.43 | 28.25 | 29.26 | 100.00 | 45.98 | 66.59 | 68.37 | 73.08 | 70.67 | 35.53 |
| **P. fluorescens** | 25.26 | 26.36 | 29.24 | 45.98 | 100.00 | 42.46 | 45.90 | 43.22 | 45.73 | 34.12 |
| **B. cepacia** | 28.36 | 29.18 | 30.28 | 66.59 | 42.46 | 100.00 | 66.45 | 62.50 | 66.67 | 36.96 |
| **C. gilardii** | 30.10 | 30.54 | 30.85 | 68.37 | 45.90 | 66.45 | 100.00 | 69.97 | 84.98 | 35.27 |
| **P. otitidis** | 28.43 | 30.00 | 29.01 | 73.08 | 43.22 | 62.50 | 69.97 | 100.00 | 73.56 | 35.79 |
| **R. pickettii** | 28.54 | 28.86 | 30.53 | 70.67 | 45.73 | 66.67 | 84.98 | 73.56 | 100.00 | 35.61 |
| **S. maltophilia** | 41.19 | 42.14 | 40.77 | 35.53 | 34.12 | 36.96 | 35.27 | 35.79 | 35.61 | 100.00 |

**Supplementary Figure 2: Protein structures of KynU**

*Burkholderia cepacia* (predicted)


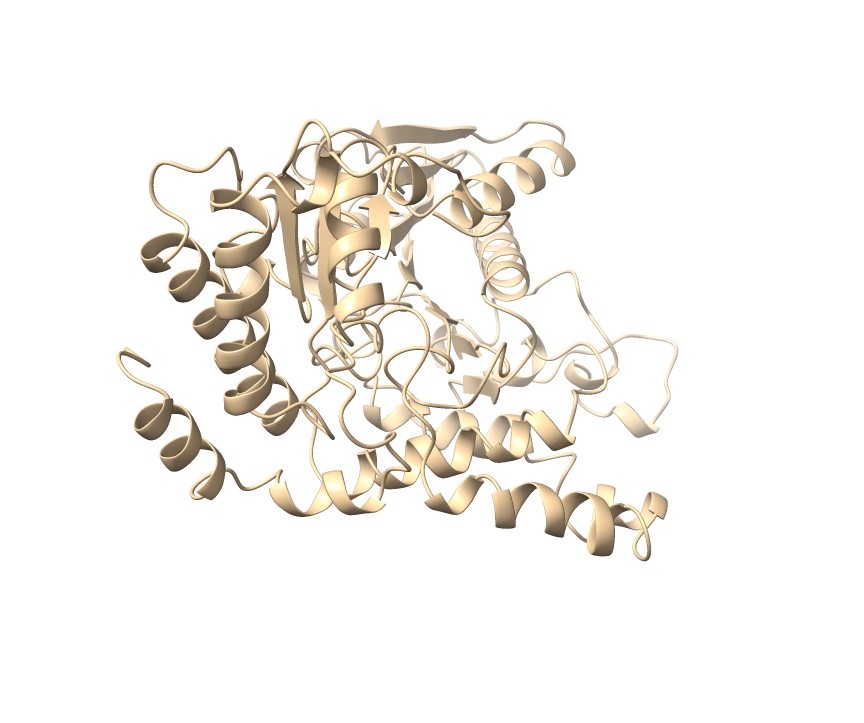


*Cupriavidus gilardii* (predicted)


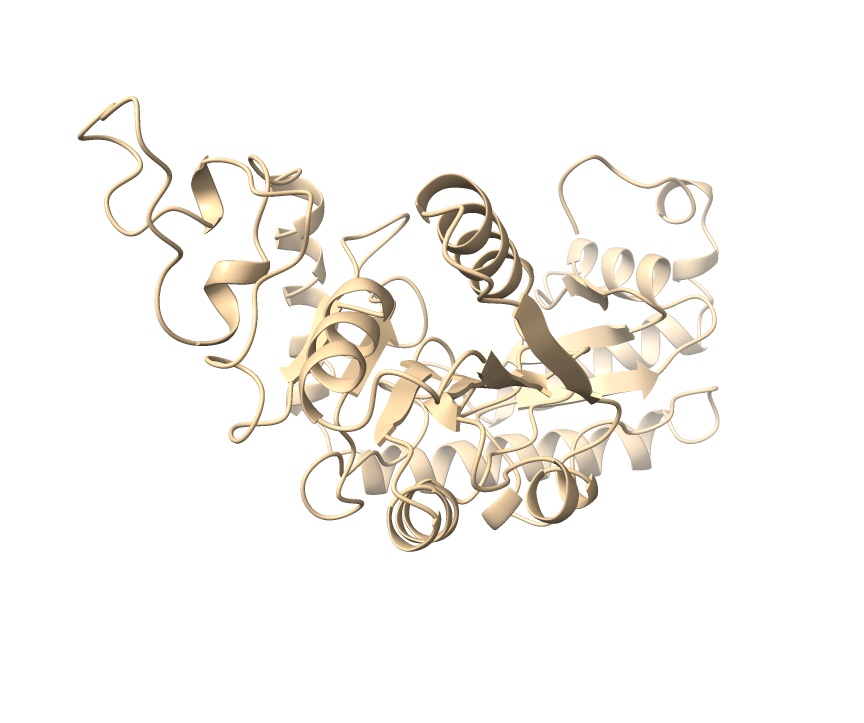


*Homo sapiens* (pdb_00002hzp)


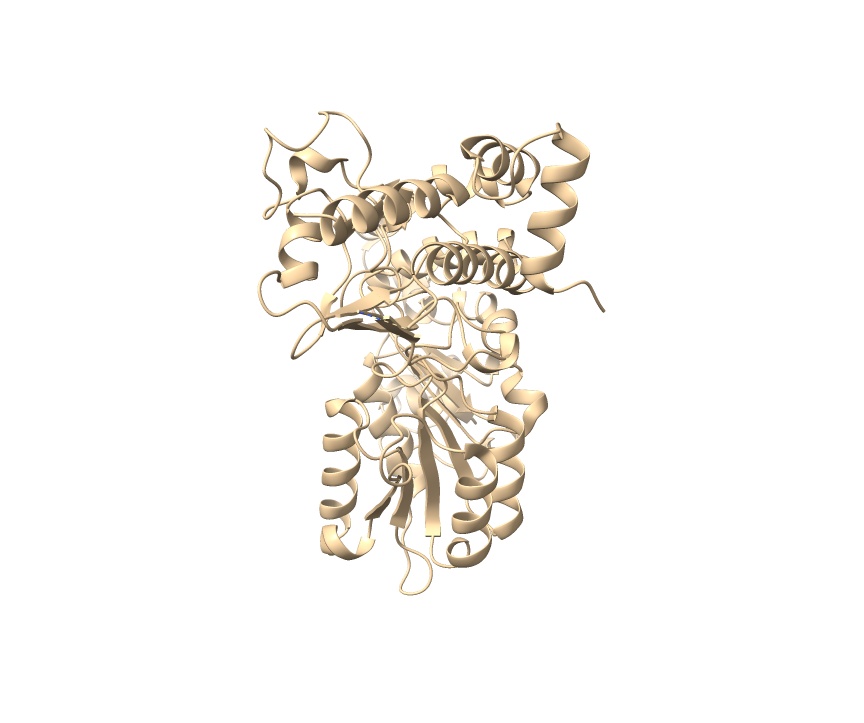


*Mus musculus* (predicted)


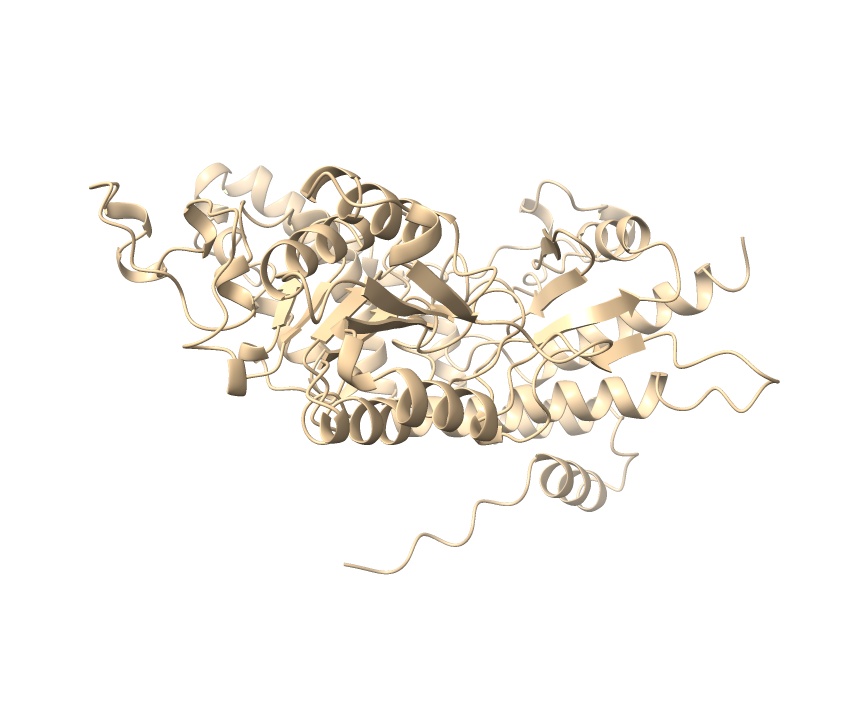


*Pseudomonas aeruginosa* (predicted)


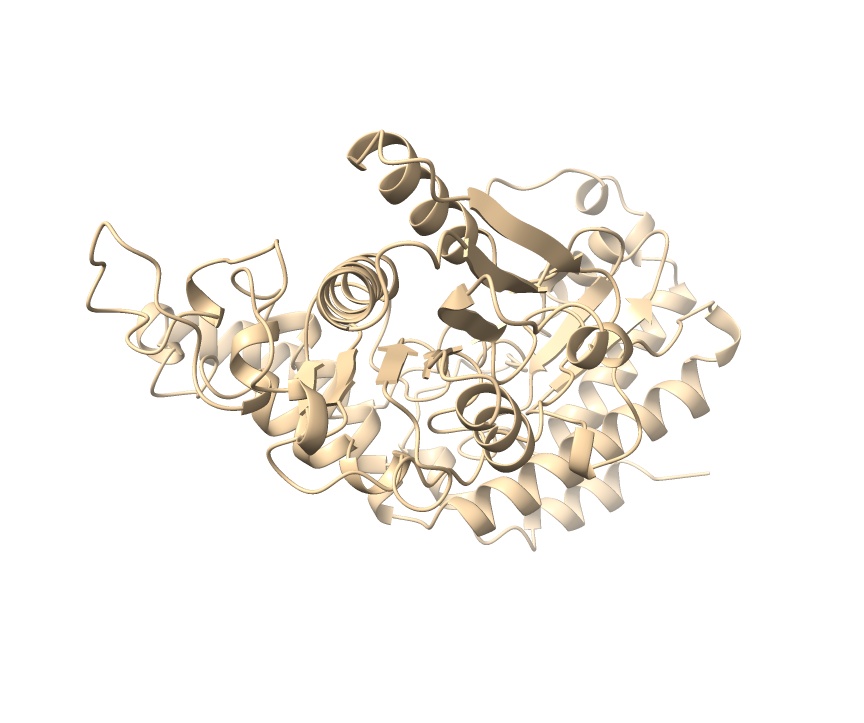


*Pseudomonas fluorescens* (pdb_00001qz9)


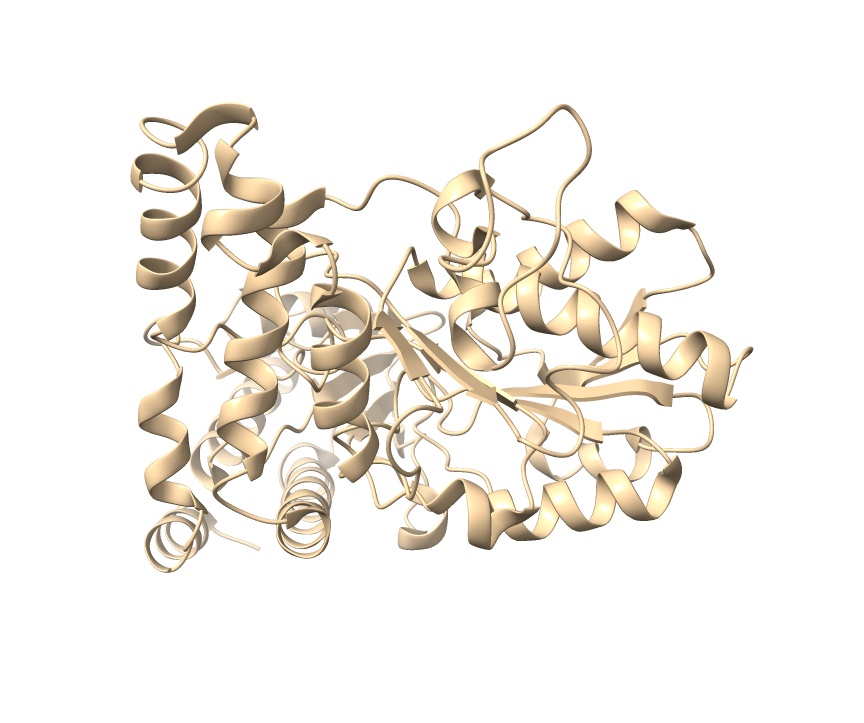


*Pseudomonas otitidis* (predicted)


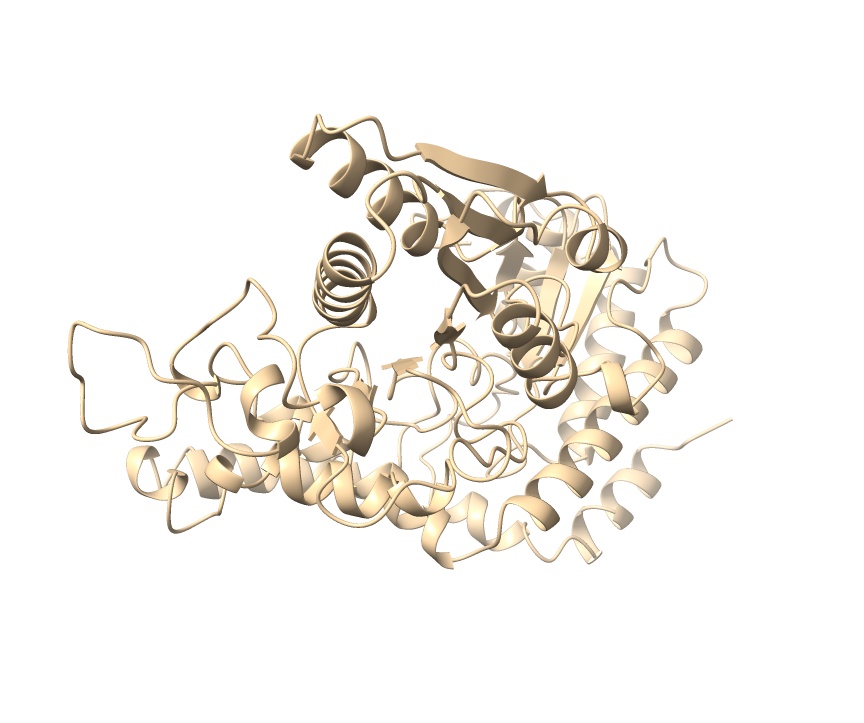


*Saccharomyces cerevisiae* (predicted)


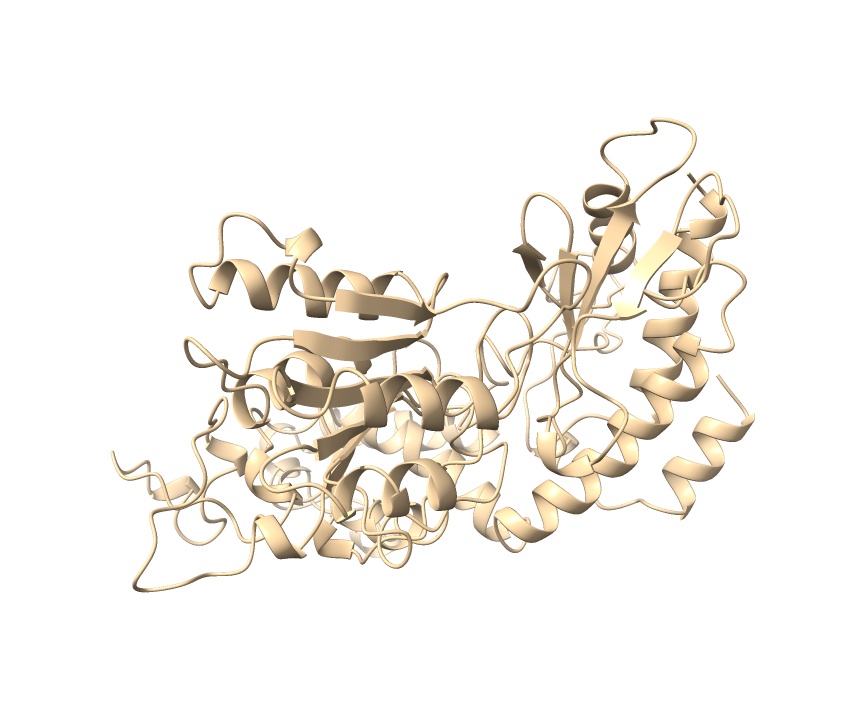


*Stenotrophomonas maltophilia* (predicted)


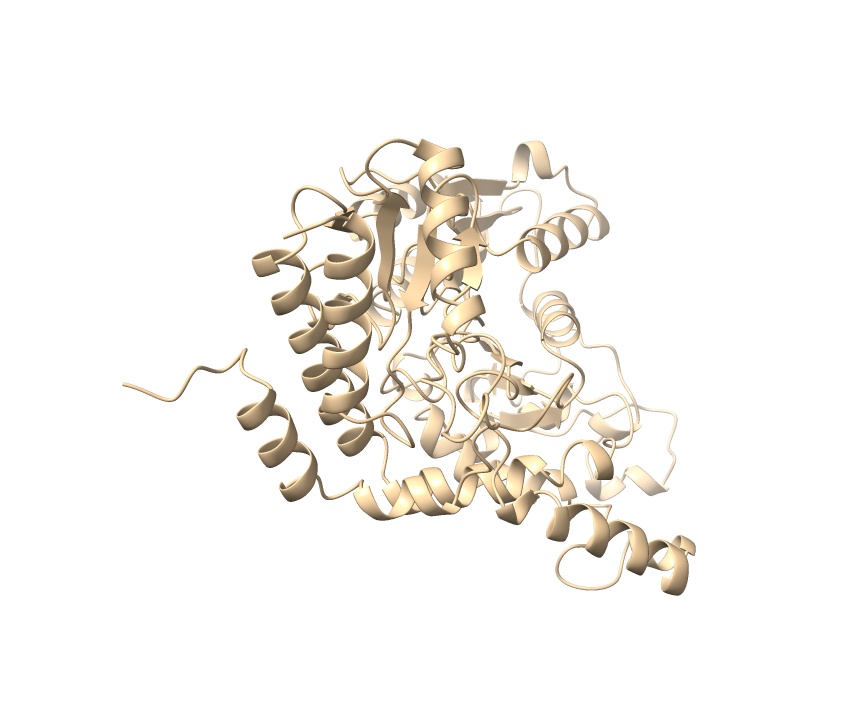


**Supplementary Figure 3: Superimposed 3D structures of KynU**

*Homo sapiens* (tan) vs *Mus musculus* (blue)


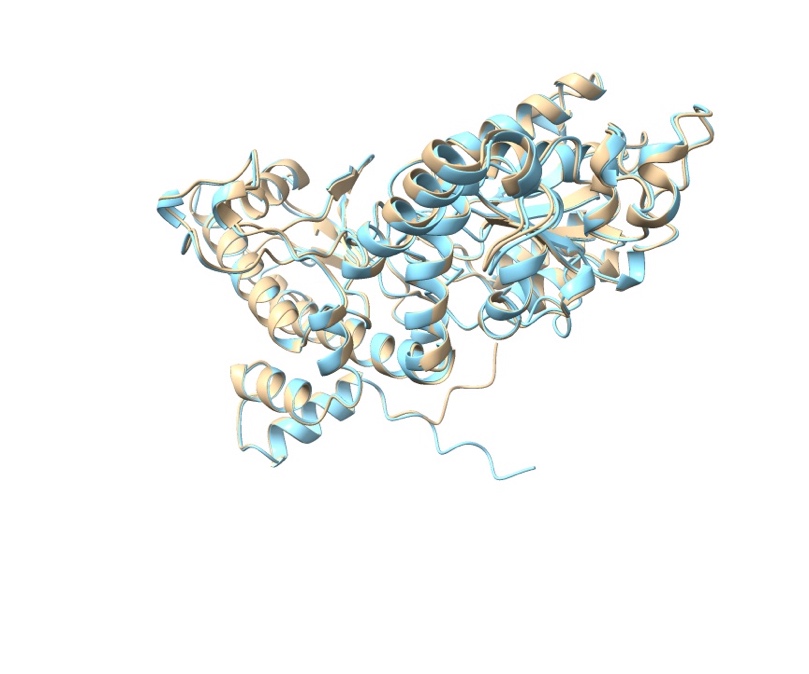


*Homo sapiens* (tan) vs *Saccharomyces cerevisiae* (blue)

**
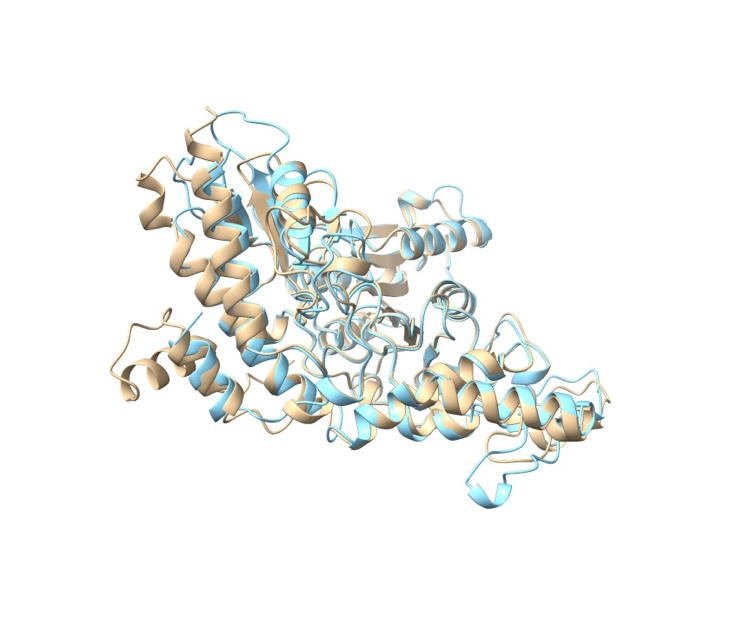
**

*Homo sapiens* (tan) vs *Pseudomonas aeruginosa* (blue)

**
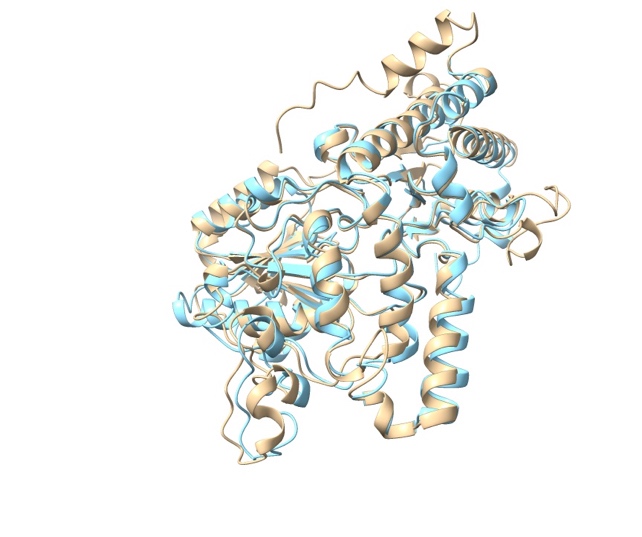
**

*Homo sapiens* (tan) vs *Pseudomonas fluorescens* (blue)

**
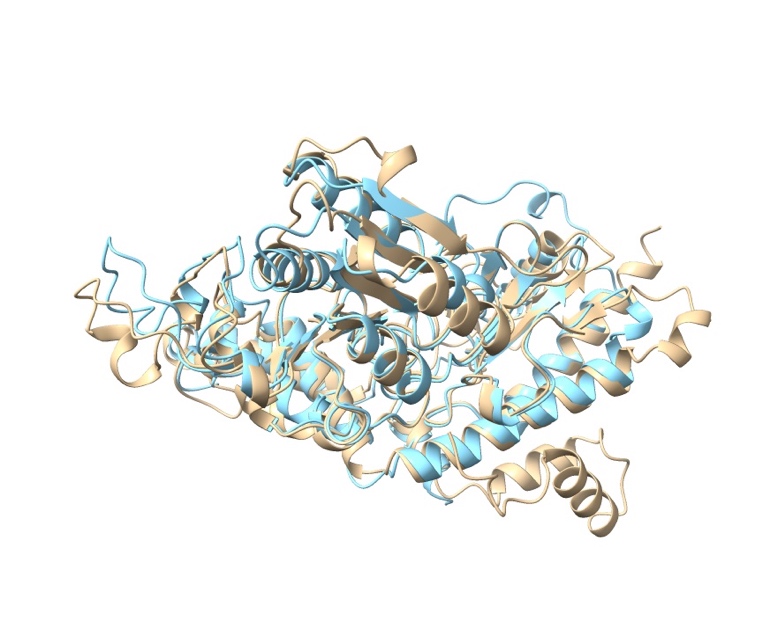
**

*Homo sapiens* (tan) vs *Burkholderia cepacia* (blue)

**
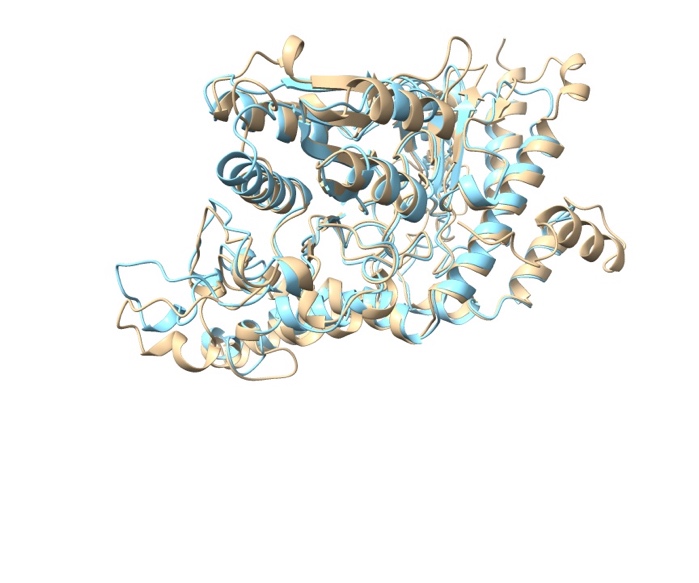
**

*Homo sapiens* (tan) vs *Cupriavidus gilardii* (blue)

**
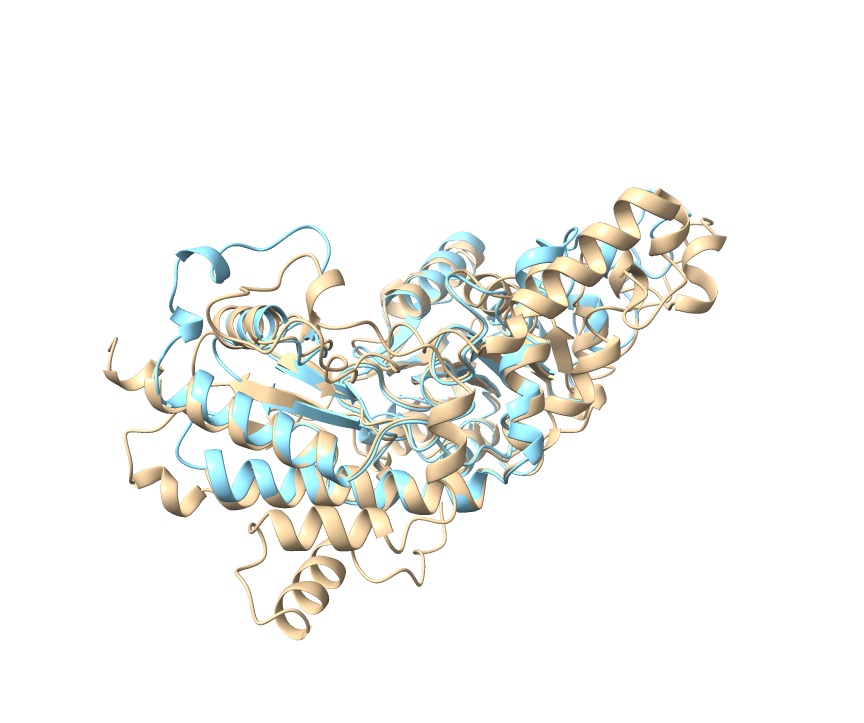
**

*Homo sapiens* (tan) vs *Pseudomonas otitidis* (blue)

**
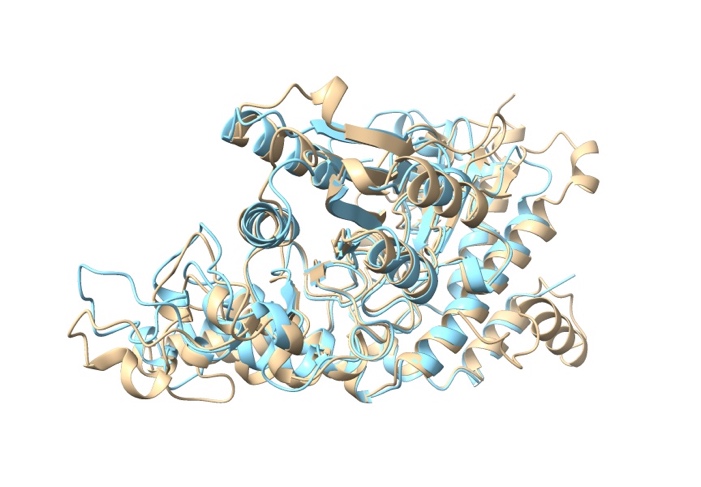
**

*Homo sapiens* (tan) vs *Stenotrophomonas maltophilia* (blue)


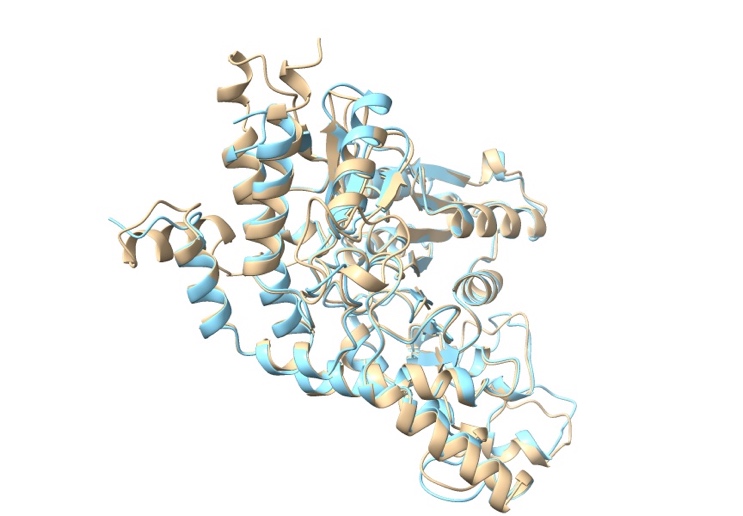


*Mus musculus* (tan) vs *Saccharomyces cerevisiae* (blue)


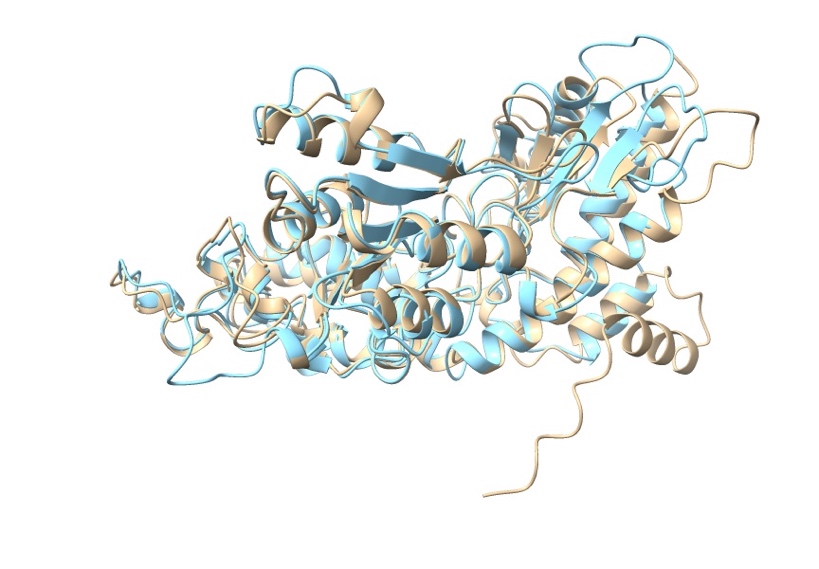


*Mus musculus* (tan) vs *Pseudomonas aeruginosa* (blue)


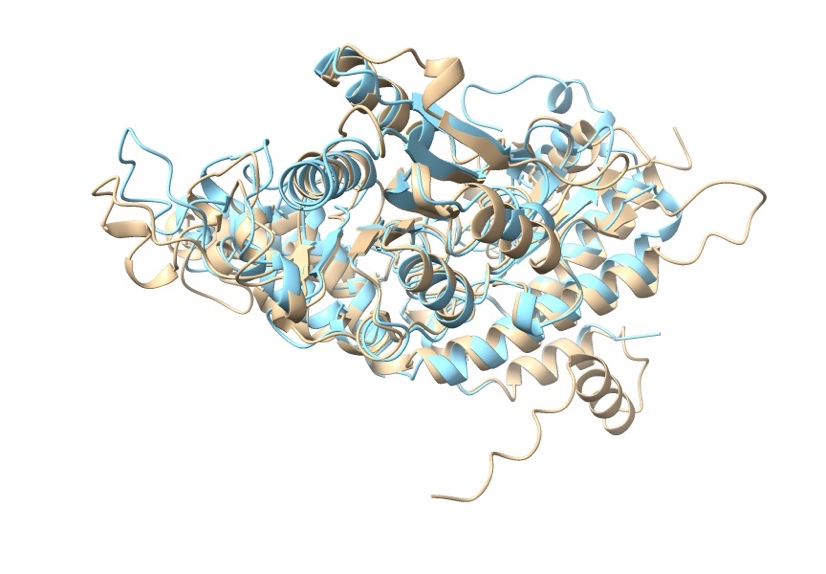


*Mus musculus* (tan) vs *Pseudomonas fluorescens* (blue)


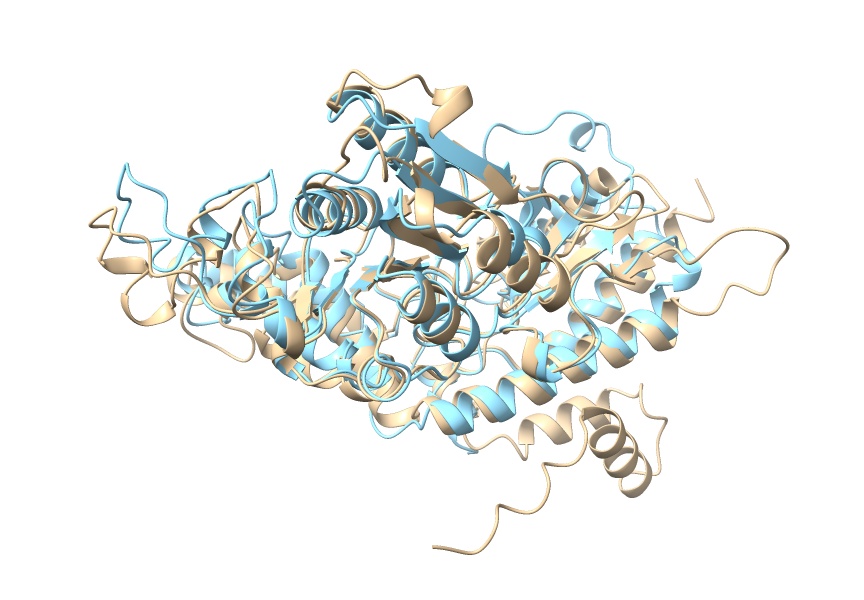


*Mus musculus* (tan) vs *Burkholderia cepacia* (blue)


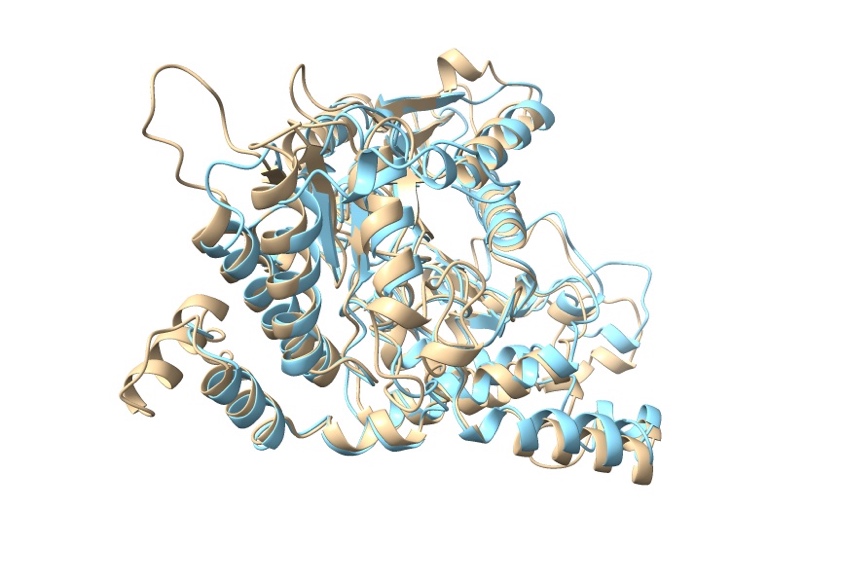


*Mus musculus* (tan) vs *Cupriavidus gilardii* (blue)


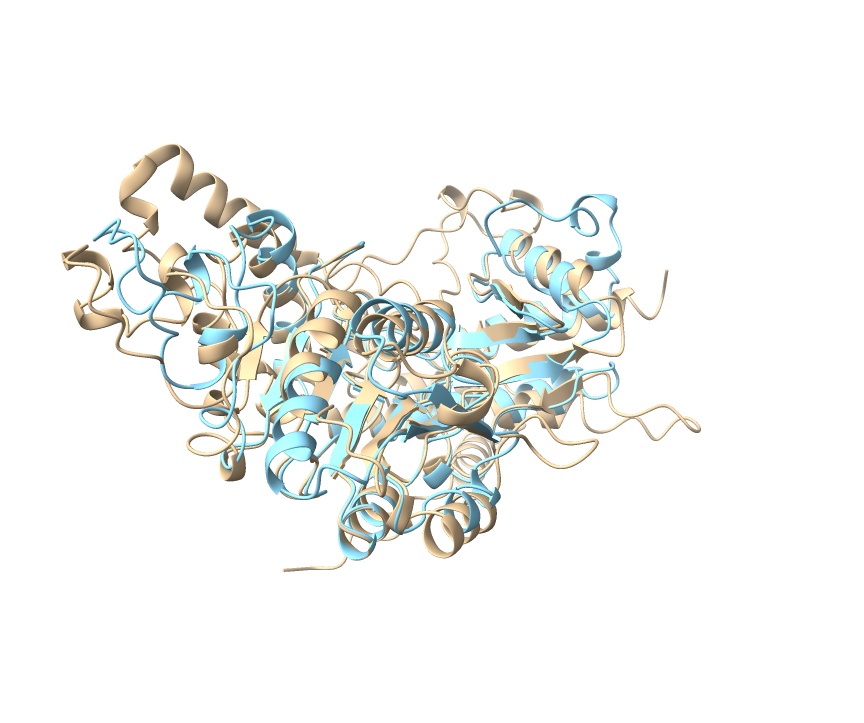


*Mus musculus* (tan) vs *Pseudomonas otitidis* (blue)


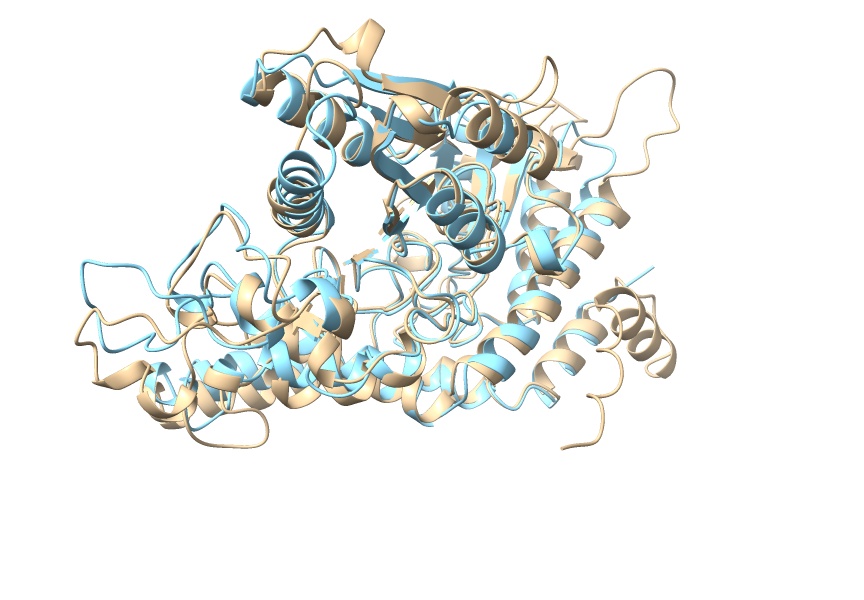


*Mus musculus* (tan) vs *Stenotrophomonas maltophilia* (blue)


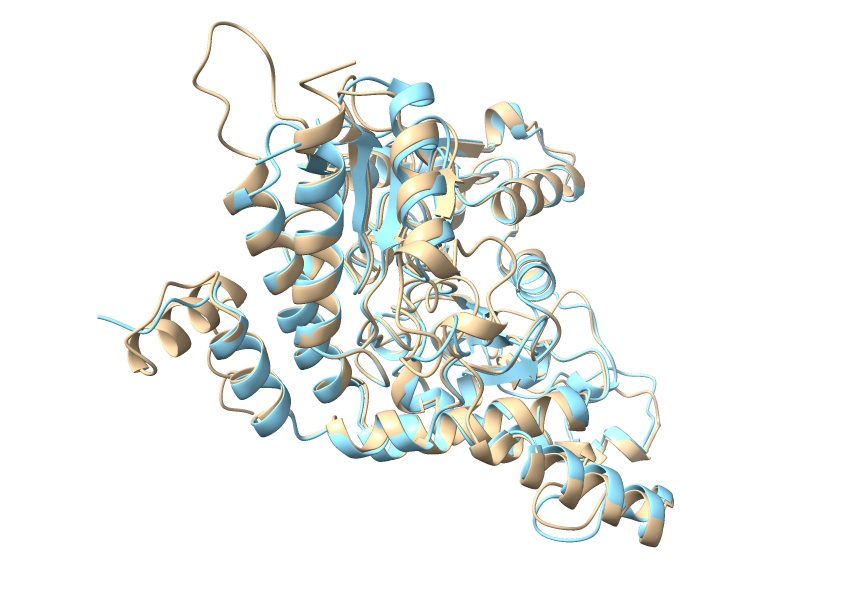


*Saccharomyces cerevisiae* (tan) vs *Pseudomonas aeruginosa* (blue)


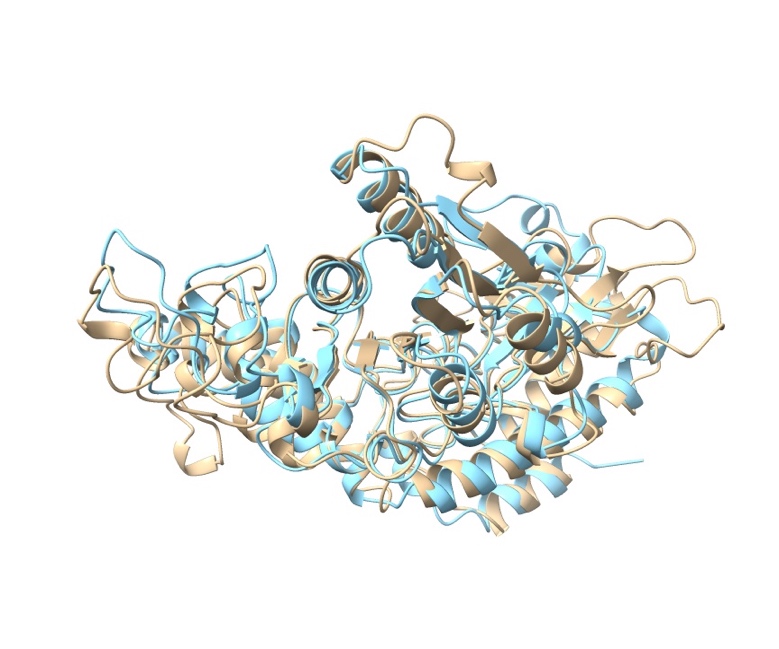


*Saccharomyces cerevisiae* (tan) vs *Pseudomonas fluorescens* (blue)


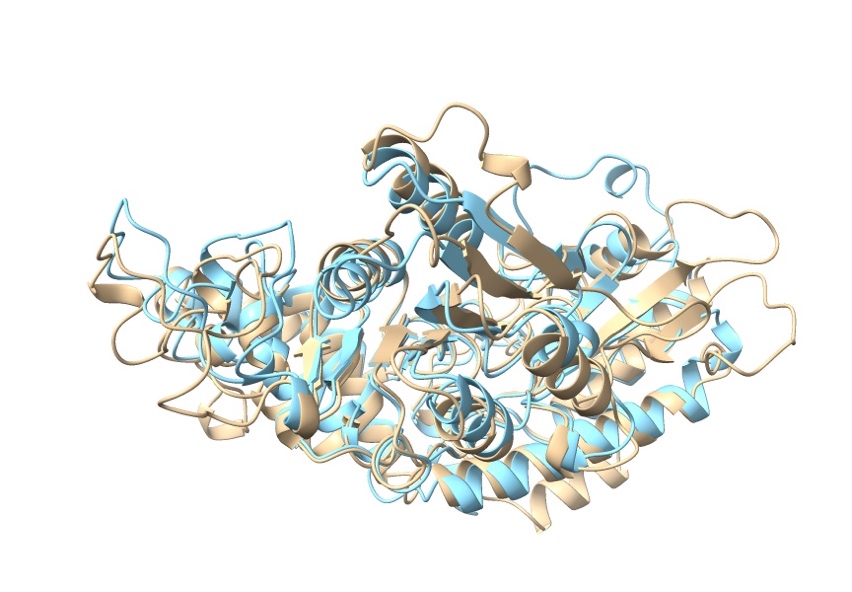


*Saccharomyces cerevisiae* (tan) vs *Burkholderia cepacia* (blue)


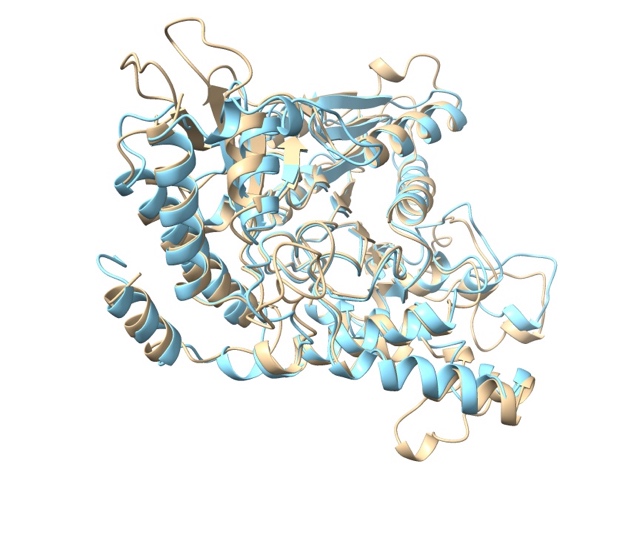


*Saccharomyces cerevisiae* (tan) vs *Cupriavidus gilardii* (blue)


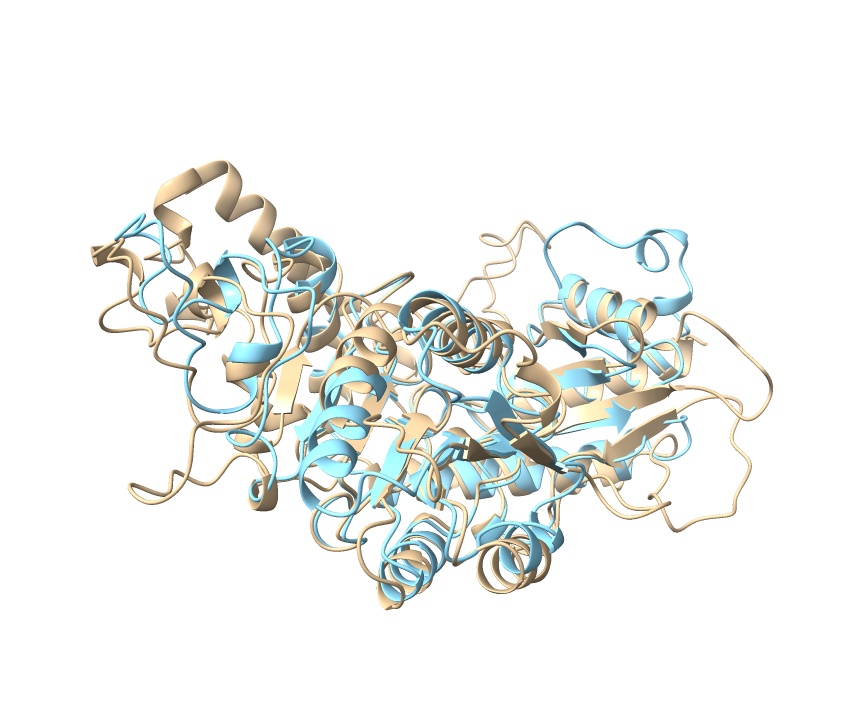


*Saccharomyces cerevisiae* (tan) vs *Pseudomonas otitidis* (blue)


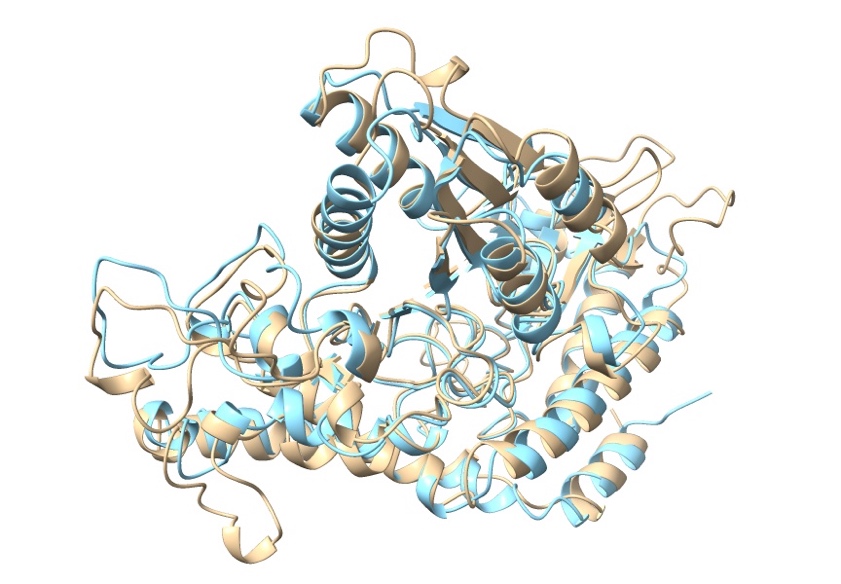


*Saccharomyces cerevisiae* (tan) vs *Stenotrophomonas maltophilia* (blue)


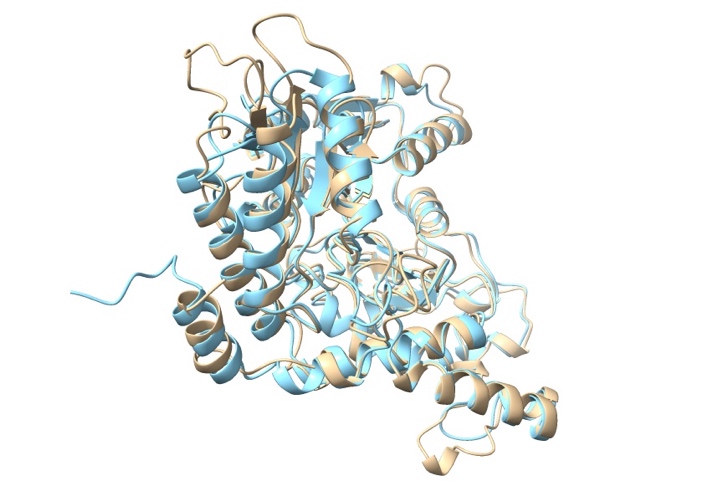


*Pseudomonas aeruginosa* (tan) vs *Pseudomonas fluorescens* (blue)


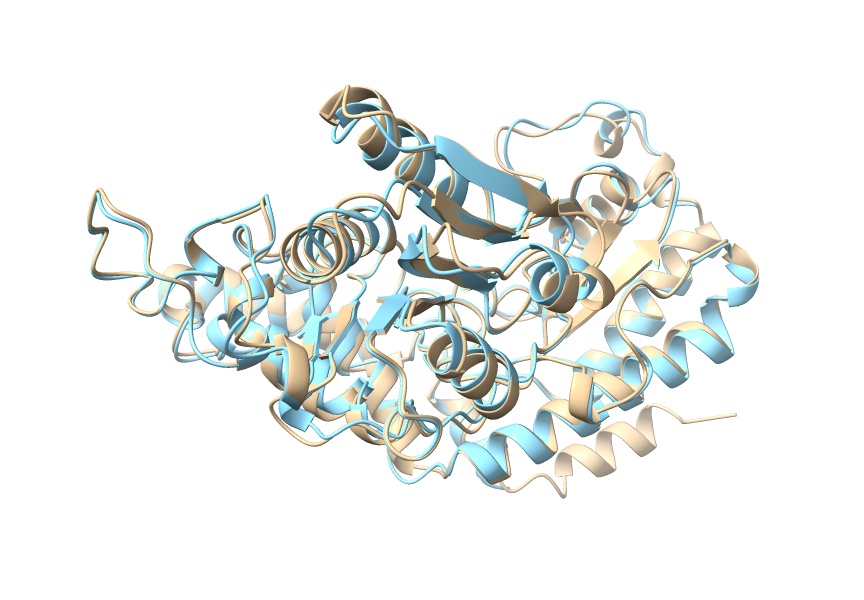


*Pseudomonas aeruginosa* (tan) vs *Burkholderia cepacia* (blue)


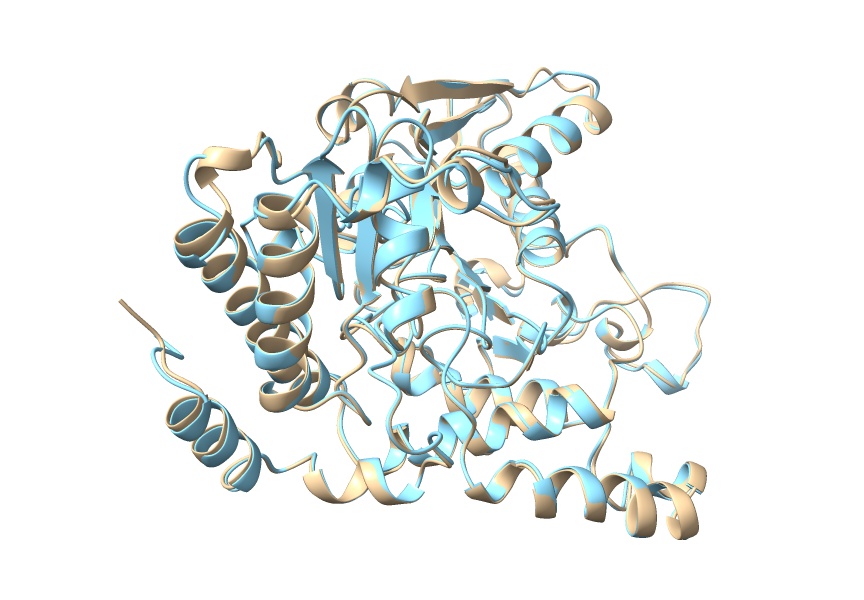


*Pseudomonas aeruginosa* (tan) vs *Cupriavidus gilardii* (blue)


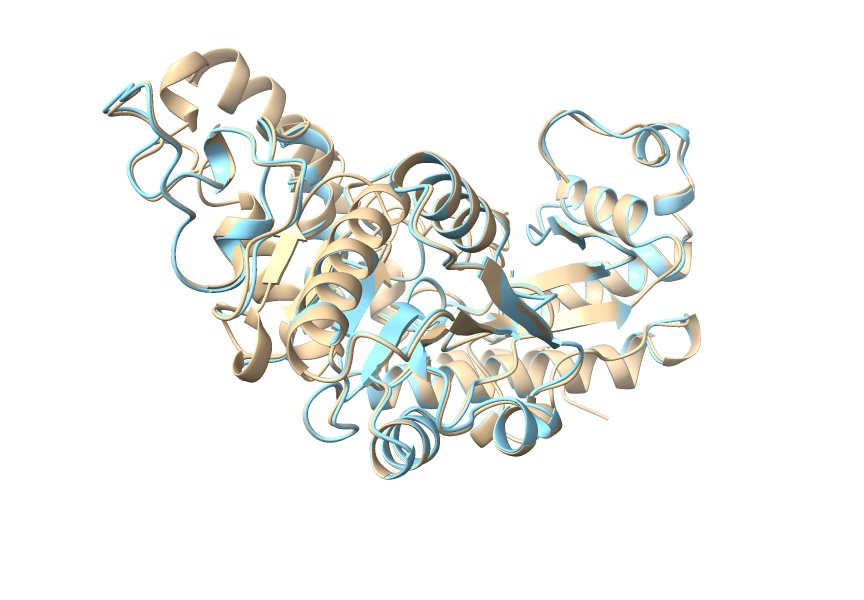


*Pseudomonas aeruginosa* (tan) vs *Pseudomonas otitidis* (blue)


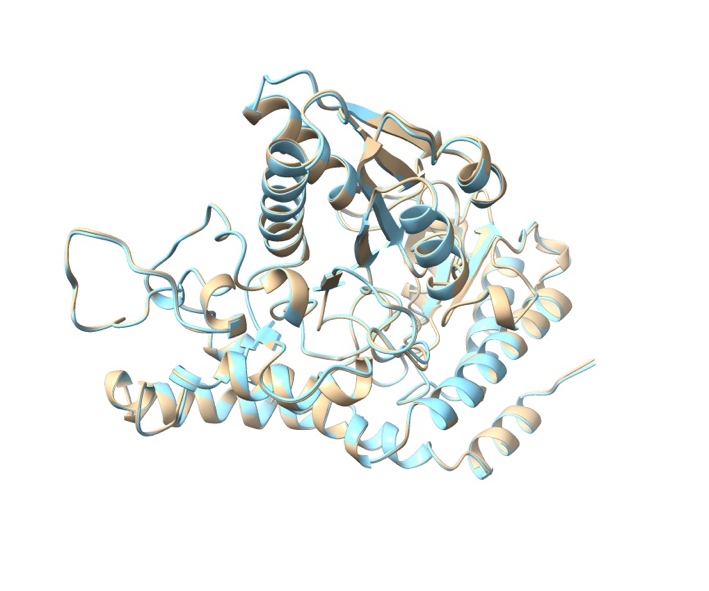


*Pseudomonas aeruginosa* (tan) vs *Stenotrophomonas maltophilia* (blue)


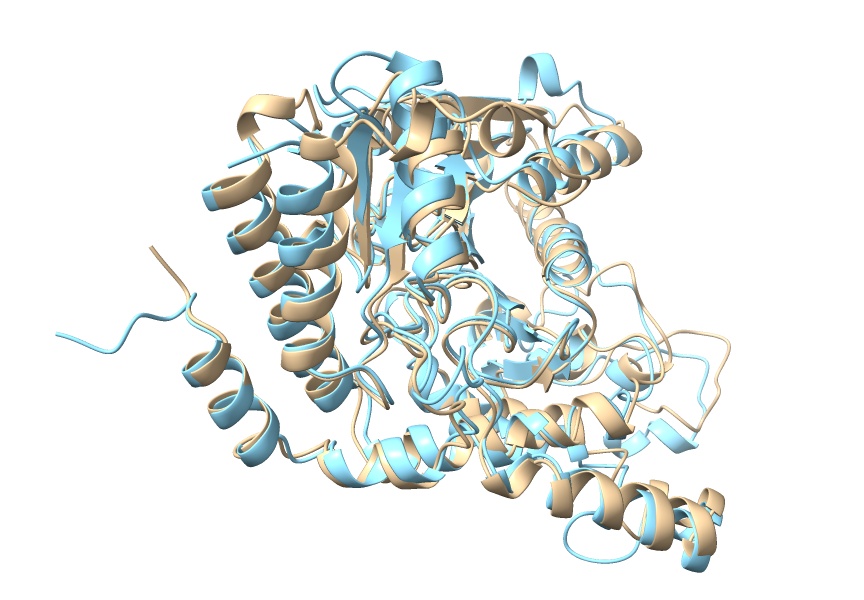


*Pseudomonas fluorescens* (tan) vs *Burkholderia cepacia* (blue)


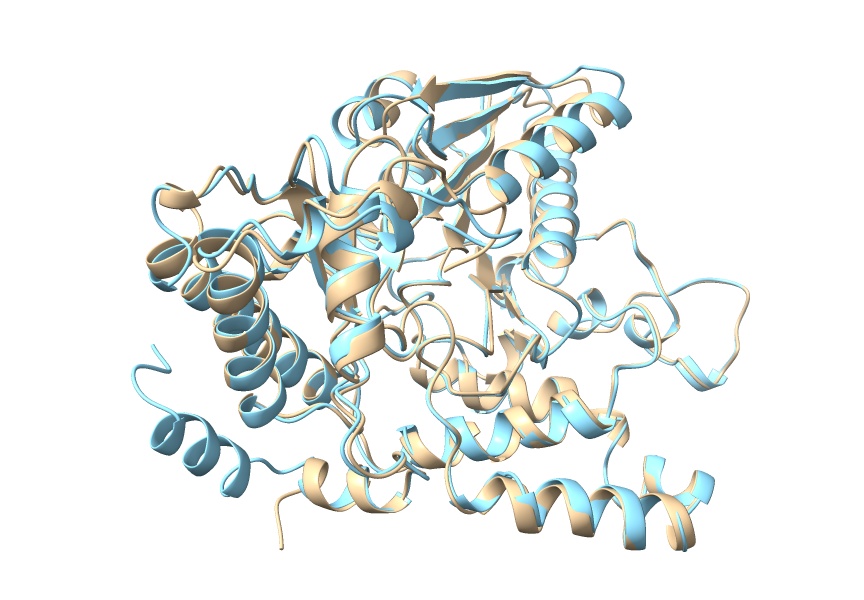


*Pseudomonas fluorescens* (tan) vs *Cupriavidus gilardii* (blue)


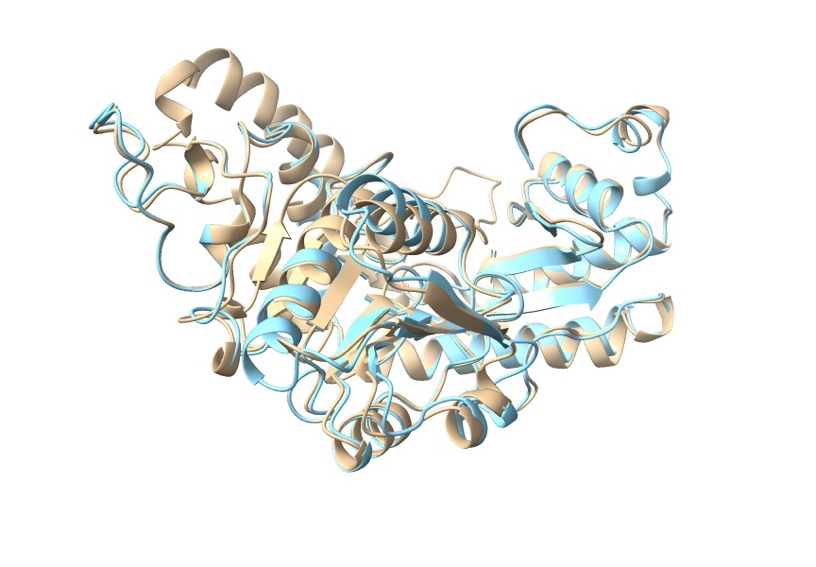


*Pseudomonas fluorescens* (tan) vs *Pseudomonas otitidis* (blue)


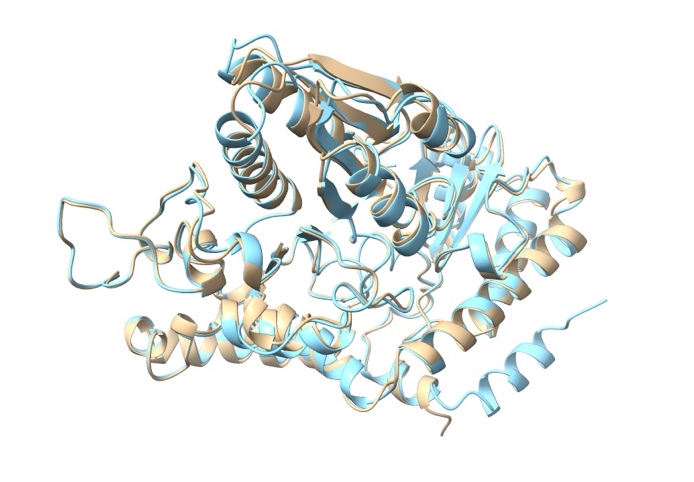


*Pseudomonas fluorescens* (tan) vs *Stenotrophomonas maltophilia* (blue)


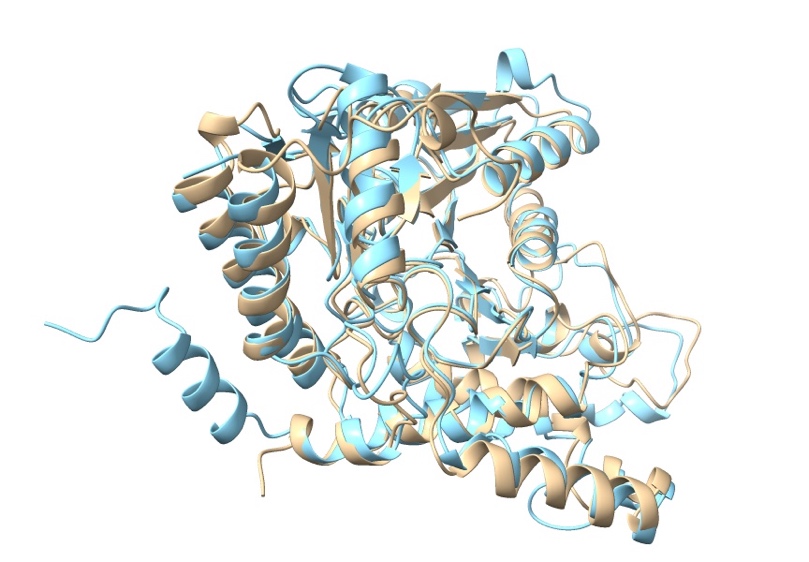


*Burkholderia cepacia* (tan) vs *Cupriavidus gilardii* (blue)


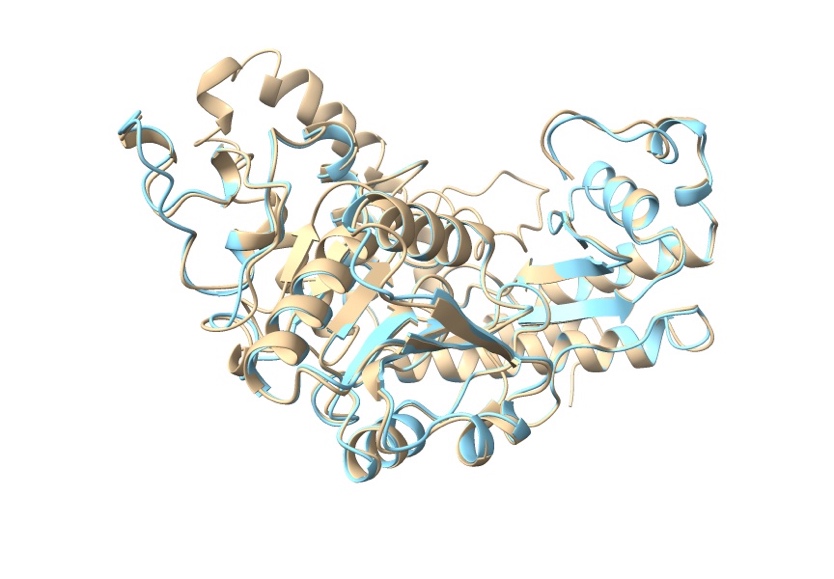


*Burkholderia cepacia* (tan) vs *Pseudomonas otitidis* (blue)


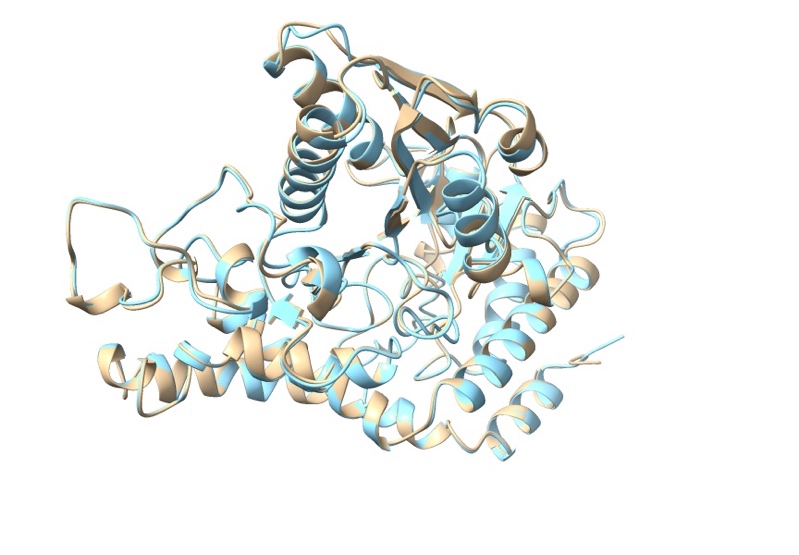


*Burkholderia cepacia* (tan) vs *Stenotrophomonas maltophilia* (blue)


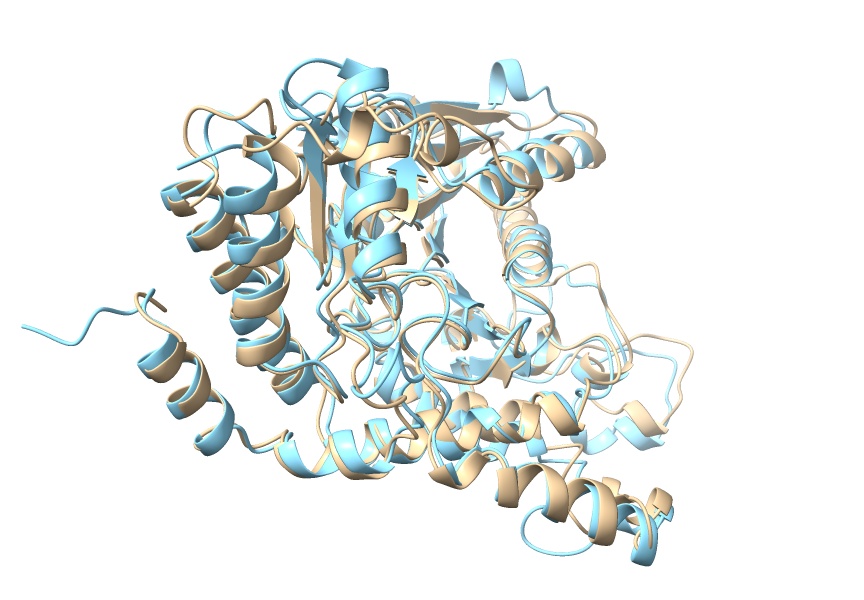


*Cupriavidus gilardii* (tan) vs *Pseudomonas otitidis* (blue)


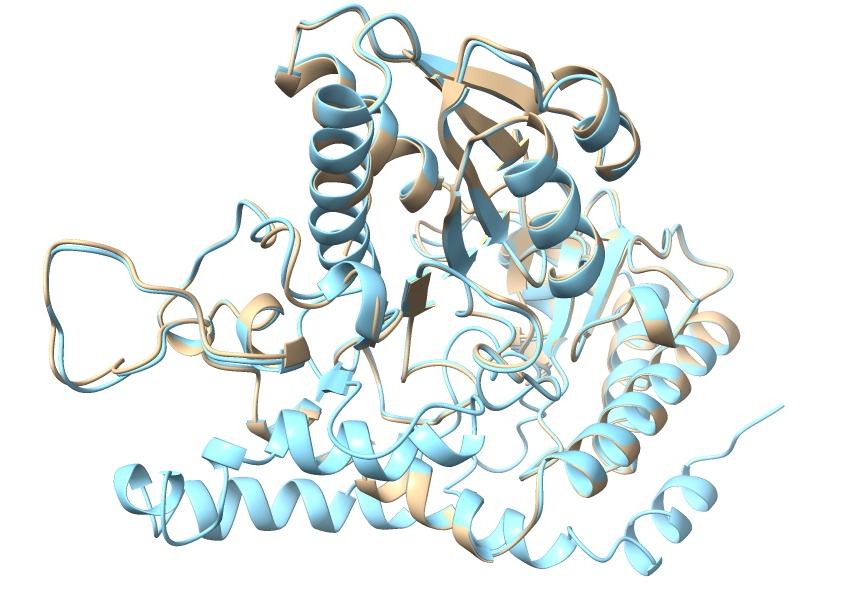


*Cupriavidus gilardii* (tan) vs *Stenotrophomonas maltophilia* (blue)


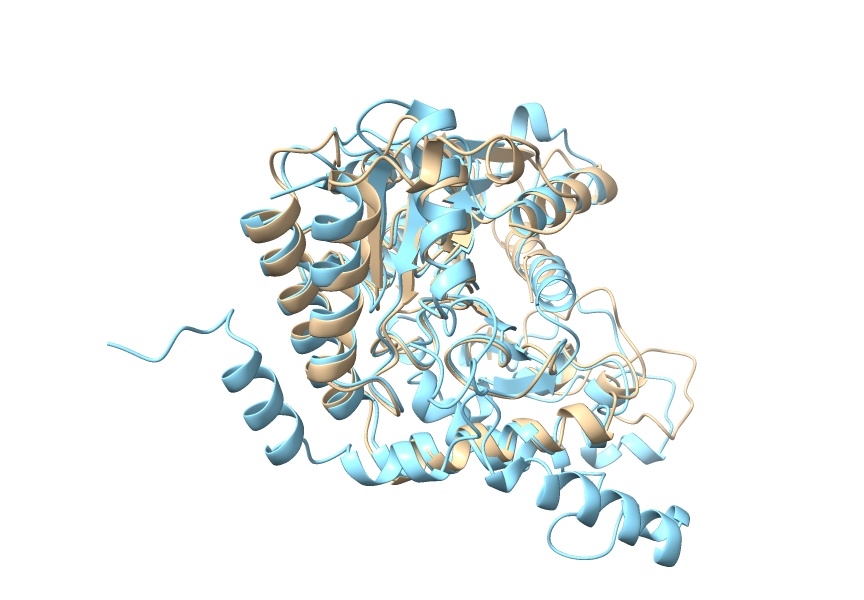


*Pseudomonas otitidis* (tan) vs *Stenotrophomonas maltophilia* (blue)


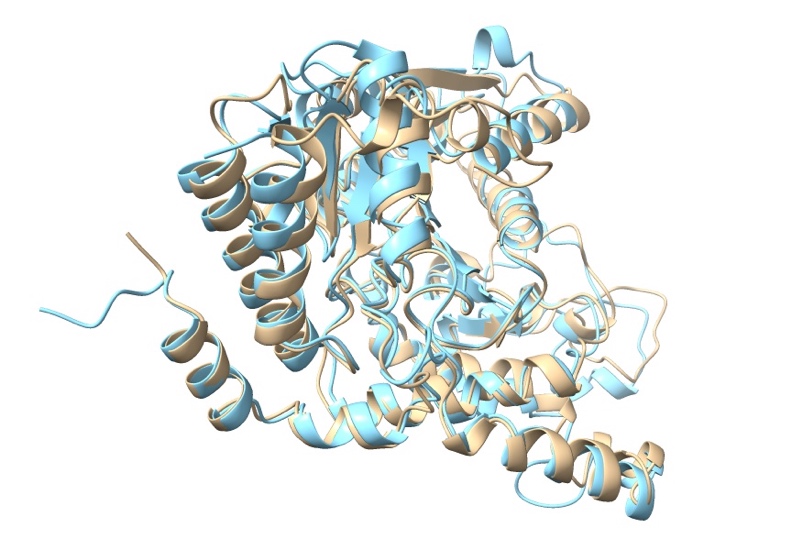


**Supplementary Table 3a: TM-score value matrix**

|  | **H. sapiens** | **M. musculus** | **S. cerevisiae** | **P. aeruginosa** | **P. fluorescens** | **B. cepacia** | **C. gilardii** | **P. otitidis** | **S. maltophilia** |
| --- | --- | --- | --- | --- | --- | --- | --- | --- | --- |
| **H. sapiens** | 1.0000 | 0.7931 | 0.2053 | 0.1604 | 0.1745 | 0.1622 | 0.1609 | 0.1601 | 0.2088 |
| **M. musculus** | 0.7931 | 1.0000 | 0.2033 | 0.1645 | 0.1718 | 0.1615 | 0.1549 | 0.1640 | 0.1953 |
| **S. cerevisiae** | 0.2053 | 0.2033 | 1.0000 | 0.2316 | 0.1515 | 0.2299 | 0.1746 | 0.2322 | 0.3210 |
| **P. aeruginosa** | 0.1604 | 0.1645 | 0.2316 | 1.0000 | 0.2035 | 0.7823 | 0.1575 | 0.9984 | 0.2635 |
| **P. fluorescens** | 0.1745 | 0.1718 | 0.1515 | 0.2035 | 1.0000 | 0.2082 | 0.1450 | 0.1992 | 0.1660 |
| **B. cepacia** | 0.1622 | 0.1615 | 0.2299 | 0.7823 | 0.2082 | 1.0000 | 0.1700 | 0.7839 | 0.2759 |
| **C. gilardii** | 0.1609 | 0.1549 | 0.1746 | 0.1575 | 0.1450 | 0.1700 | 1.0000 | 0.1571 | 0.1575 |
| **P. otitidis** | 0.1601 | 0.1640 | 0.2322 | 0.9984 | 0.1992 | 0.7839 | 0.1571 | 1.0000 | 0.2631 |
| **S. maltophilia** | 0.2088 | 0.1953 | 0.3210 | 0.2635 | 0.1660 | 0.2759 | 0.1575 | 0.2631 | 1.0000 |

**Supplementary Table 3b: RMSD value matrix**

|  | **H. sapiens** | **M. musculus** | **S. cerevisiae** | **P. aeruginosa** | **P. fluorescens** | **B. cepacia** | **C. gilardii** | **P. otitidis** | **S. maltophilia** |
| --- | --- | --- | --- | --- | --- | --- | --- | --- | --- |
| **H. sapiens** | 0.000 | 0.386 | 0.350 | 0.334 | 0.317 | 0.332 | 0.353 | 0.352 | 0.348 |
| **M. musculus** | 0.386 | 0.000 | 0.343 | 0.362 | 0.312 | 0.369 | 0.348 | 0.357 | 0.308 |
| **S. cerevisiae** | 0.350 | 0.343 | 0.000 | 0.340 | 0.338 | 0.384 | 0.337 | 0.324 | 0.207 |
| **P. aeruginosa** | 0.334 | 0.362 | 0.340 | 0.000 | 0.345 | 0.388 | 0.347 | 0.030 | 0.340 |
| **P. fluorescens** | 0.317 | 0.312 | 0.338 | 0.345 | 0.000 | 0.361 | 0.359 | 0.281 | 0.231 |
| **B. cepacia** | 0.332 | 0.369 | 0.384 | 0.388 | 0.361 | 0.000 | 0.350 | 0.386 | 0.344 |
| **C. gilardii** | 0.353 | 0.348 | 0.337 | 0.347 | 0.359 | 0.350 | 0.000 | 0.336 | 0.351 |
| **P. otitidis** | 0.352 | 0.357 | 0.324 | 0.030 | 0.281 | 0.386 | 0.336 | 0.000 | 0.354 |
| **S. maltophilia** | 0.348 | 0.308 | 0.207 | 0.340 | 0.231 | 0.344 | 0.351 | 0.354 | 0.000 |

**Supplementary Table 4: Functional test**

Kynurenine

| Conc. (µM) | Peak area |
| --- | --- |
| 0.1 | 17854.998 |
| 0.5 | 43292.824 |
| 1 | 83142.977 |
| 5 | 439600.625 |
| 10 | 887509.5 |
| 50 | 3358133.25 |
| 100 | 5730771.5 |
| 500 | 18898204 |

1 = kynurenine addition, 2 = without kynurenine, a = extracellular extraction, b = intracellular extraction, M = medium, BD = pBAD24, dilution is 1:10 dilution unless specific dilution is indicated.

| Batch | Samples | RT | Peak | Conc. (µM) | From dilution (µM) |
| --- | --- | --- | --- | --- | --- |
| 1st | @RP1a | 4.93 | 21202008 | 551.652383116362 | 436.055361009383 |
| 1st | @RP2a |  |  |  |  |
| 1st | @SM1a | 4.93 | 22672838 | 590.969927022909 | 701.683017455692 |
| 1st | @SM2a |  |  |  |  |
| 1st | @RP1b | 4.95 | 828075.563 | 7.02589117592023 |  |
| 1st | @SM1b | 4.95 | 794329.813 | 6.12381547221257 |  |
| 1st | @RP2b | 4.95 | 73714.063 | -13.1393498088695 |  |
| 1st | @SM2b |  |  |  |  |
| 1st | @PA1a | 4.92 | 20672340 | 537.493544334251 | 710.881004571092 |
| 1st | @PO1a |  |  |  |  |
| 1st | @PO2a |  |  |  |  |
| 1st | @PA2a |  |  |  |  |
| 1st | @PO2b |  |  |  |  |
| 1st | @PA2b |  |  |  |  |
| 1st | @PO1b |  |  |  |  |
| 1st | @PA1b | 5.07 | 713231.875 | 3.95594308856158 |  |
| 1st | @BC1a |  |  |  |  |
| 1st | @BC2a |  |  |  |  |
| 1st | @CG2a |  |  |  |  |
| 1st | @CG1a |  |  |  |  |
| 1st | @BC1b | 5.03 | 823809.375 | 6.91184942126226 |  |
| 1st | @BC2b |  |  |  |  |
| 1st | @CG2b |  |  |  |  |
| 1st | @CG1b | 5.03 | 1096579.125 | 14.2034035927183 |  |
| 1st | @BD1a | 5 | 22413752 | 584.044160496137 | 866.095792456361 |
| 1st | @BD2a |  |  |  |  |
| 1st | @BD2b |  |  |  |  |
| 1st | @BD1b | 5.01 | 1397279.625 | 22.2415895907402 |  |
| 1st | @M1 | 4.74 | 27127686 | 710.054853110214 | 630.218610494801 |
| 1st | @M2 | 4.77 | 4217.266 | -14.9971058836109 |  |
| 1st | @HS2a |  |  |  |  |
| 1st | @PF1a | 6.01 | 13002888 | 332.47731829239 |  |
| 1st | @PF2b |  |  |  |  |
| 1st | @HS1b | 5.99 | 473361.688 | -2.4561552567564 |  |
| 1st | @HS2b | 6.23 | 38772.543 | -14.0733902804138 |  |
| 1st | @HS1a | 5.95 | 13848483 | 355.081370793125 |  |
| 1st | @PF2a |  |  |  |  |
| 1st | @PF1b | 5.92 | 571549.563 | 0.16855737924029 |  |
|  |  |  |  |  |  |
| 2nd | @HS1a | 5.88 | 15193057 | 391.023897992462 |  |
| 2nd | @PF2a |  |  |  |  |
| 2nd | @PF1a | 5.85 | 14790860 | 380.272554732818 |  |
| 2nd | @HS2a |  |  |  |  |
| 2nd | @HS2b |  |  |  |  |
| 2nd | @PF1b | 5.86 | 145412.016 | -11.2227534550509 |  |
| 2nd | @HS1b | 5.85 | 96731.203 | -12.5240663209388 |  |
| 2nd | @PF2b | 6.08 | 15120.36 | -14.7056494426475 |  |
| 2nd | @M1 | 5.84 | 14355101 | 368.624047689059 |  |
| 2nd | @CG1a | 5.84 | 15660655 | 403.523510385201 |  |
| 2nd | @RP1a | 5.84 | 12277065 | 313.074955224679 |  |
| 2nd | @RP2a |  |  |  |  |
| 2nd | @CG2a |  |  |  |  |
| 2nd | @CG2b |  |  |  |  |
| 2nd | @RP1b | 5.84 | 182960.719 | -10.21901897939 |  |
| 2nd | @CG1b | 5.82 | 486198.438 | -2.11300922237964 |  |
| 2nd | @RP2b | 6.11 | 32366.254 | -14.2446402202678 |  |
| 2nd | @M2 |  |  |  |  |
| 2nd | @PA2a |  |  |  |  |
| 2nd | @PA1a | 5.78 | 13122557 | 335.676254377289 |  |
| 2nd | @SM1a | 5.78 | 14368106 | 368.971691304232 |  |
| 2nd | @SM2a |  |  |  |  |
| 2nd | @PA2b |  |  |  |  |
| 2nd | @SM1b | 5.8 | 177501.313 | -10.3649572830068 |  |
| 2nd | @PA1b | 5.77 | 256044.063 | -8.26538899730011 |  |
| 2nd | @SM2b |  |  |  |  |
| 2nd | @PO2a |  |  |  |  |
| 2nd | @PO1a | 5.74 | 8577979 | 214.1927076372 |  |
| 2nd | @BC2a |  |  |  |  |
| 2nd | @BC1a | 5.73 | 7929578 | 196.859953487129 |  |
| 2nd | @BC2b |  |  |  |  |
| 2nd | @PO2b |  |  |  |  |
| 2nd | @BC1b | 5.74 | 123197.836 | -11.8165725894838 |  |
| 2nd | @PO1b | 5.74 | 137906.453 | -11.4233886765217 |  |
| 2nd | @BD2a |  |  |  |  |
| 2nd | @BD1a | 5.7 | 12669983 | 323.578256569275 |  |
| 2nd | @BD2b |  |  |  |  |
| 2nd | @BD1b | 5.72 | 73053.18 | -13.1570162260419 |  |
|  |  |  |  |  |  |
| 3rd | @M1 | 5.64 | 33910988 | 891.382929241626 | 647.589136304098 |
| 3rd | @M2 | 5.66 | 6244.378 | -14.9429180678446 |  |
| 3rd | @PA-1a | 5.58 | 29731532 | 779.659654093935 | 1067.28193750167 |
| 3rd | @SM-1a | 5.55 | 33726960 | 886.463578283301 | 1320.01938036301 |
| 3rd | @CG-1a | 5.55 | 33466852 | 879.510492127563 | 950.413964553984 |
| 3rd | @BC-1a | 5.54 | 30328492 | 795.61731134219 | 1183.61998449571 |
| 3rd | @BD-1a | 5.52 | 32621608 | 856.91582239568 | 1132.29062525061 |
| 3rd | @RP-1a | 5.49 | 33805884 | 888.573337966799 | 1106.99457349836 |
| 3rd | @SM-2a | 5.54 | 669.802 | -15.0919350423695 |  |
| 3rd | @BD-2a | 5.53 | 3930.629 | -15.0047681306637 |  |
| 3rd | @PA-2a |  |  |  |  |
| 3rd | @BC-2a |  |  |  |  |
| 3rd | @CG-2a | 5.53 | 3476.641 | -15.0169039268625 |  |
| 3rd | @RP-2a |  |  |  |  |
| 3rd | @CG-2b |  |  |  |  |
| 3rd | @PA-1b | 5.48 | 528.725 | -15.0957062471598 |  |
| 3rd | @SM-2b |  |  |  |  |
| 3rd | @BC-2a |  |  |  |  |
| 3rd | @RP-1b |  |  |  |  |
| 3rd | @SM-1b |  |  |  |  |
| 3rd | @BD-2b |  |  |  |  |
| 3rd | @BD-1b |  |  |  |  |
| 3rd | @BC-1b |  |  |  |  |
| 3rd | @PA-2b |  |  |  |  |
| 3rd | @CG-1b |  |  |  |  |
| 3rd | @RP-2b |  |  |  |  |
|  |  |  |  |  |  |
| Dilution | @DSM3 | 5.35 | 5503304.5 | 132.001938036301 | 1320.01938036301 |
| Dilution | @DPA3 | 5.35 | 4557839 | 106.728193750167 | 1067.28193750167 |
| Dilution | @DBC3 | 5.34 | 4993048 | 118.361998449571 | 1183.61998449571 |
| Dilution | @DBD3 | 5.34 | 4801030 | 113.229062525061 | 1132.29062525061 |
| Dilution | @DRP3 | 5.34 | 4706400 | 110.699457349836 | 1106.99457349836 |
| Dilution | @DBD2 | 5.33 | 4676268 | 109.893982731428 | 1098.93982731428 |
| Dilution | @DBC1 | 5.32 | 3183976.5 | 70.0027399823572 | 700.027399823572 |
| Dilution | @DRP1 | 5.32 | 2196483.5 | 43.6055361009383 | 436.055361009383 |
| Dilution | @DSM1 | 5.31 | 3190170 | 70.1683017455692 | 701.683017455692 |
| Dilution | @DPA1 | 5.31 | 3224578.75 | 71.0881004571093 | 710.881004571093 |
| Dilution | @DBD1 | 5.31 | 3805221.75 | 86.6095792456361 | 866.095792456361 |
| Dilution | @DSM2 | 5.31 | 3130039 | 68.5609078029351 | 685.609078029351 |
| Dilution | @DBC2 | 5.3 | 3741806.5 | 84.9143922585474 | 849.143922585474 |
| Dilution | @DRP2 | 5.3 | 3467323.75 | 77.5770469673073 | 775.770469673073 |
| Dilution | @DPA2 | 5.29 | 3145028 | 68.961586783929 | 689.61586783929 |
|  |  |  |  |  |  |
| Dilution (1:1) | @FDM-1 | 4.69 | 12353168 | 315.1093052474 | 630.2186104948 |
| Dilution (1:1) | @TDM-1 | 4.7 | 12678075 | 323.794568152049 | 647.589136304098 |
| Dilution (1:1) | @DCG-3 | 4.69 | 18342262 | 475.206982276992 | 950.413964553984 |

Summary

|  | 1^st^ (µM) | | 2^nd^ (µM) | | 3^rd^ (µM) | |
| --- | --- | --- | --- | --- | --- | --- |
|  | intra | extra | intra | extra | intra | extra |
| Media + KYN | N/A | 630.218610494801 | N/A | 368.624047689059 | N/A | 647.589136304098 |
| Media | N/A | N/A | N/A | N/A | N/A | N/A |
| pBAD24 + KYN | 22.2415895907402 | 866.095792456361 | N/A | 323.578256569275 | N/A | 1132.29062525061 |
| pBAD24 | N/A | N/A | N/A | N/A | N/A | N/A |
| kynUPA + KYN | 3.95594308856158 | 710.881004571092 | N/A | 335.676254377289 | N/A | 1067.28193750167 |
| kynUPA | N/A | N/A | N/A | N/A | N/A | N/A |
| kynUBC + KYN | 6.91184942126226 | 700.027399823572 | N/A | 196.859953487129 | N/A | 1183.61998449571 |
| kynUBC | N/A | N/A | N/A | N/A | N/A | N/A |
| kynUCG + KYN | 14.2034035927183 | N/A | N/A | 403.523510385201 | N/A | 950.413964553984 |
| kynUCG | N/A | N/A | N/A | N/A | N/A | N/A |
| kynURP + KYN | 7.02589117592023 | 436.055361009383 | N/A | 313.074955224679 | N/A | 1106.99457349836 |
| kynURP | N/A | N/A | N/A | N/A | N/A | N/A |
| kynUSM + KYN | 6.12381547221257 | 701.683017455692 | N/A | 368.971691304232 | N/A | 1320.01938036301 |
| kynUSM | N/A | N/A | N/A | N/A | N/A | N/A |

Total kynurenine concentrations

|  | 1st total (µM) | 2nd total (µM) | 3rd total (µM) |
| --- | --- | --- | --- |
| Media + KYN | 630.218610494801 | 368.624047689059 | 647.589136304098 |
| Media | N/A | N/A | N/A |
| pBAD24 + KYN | 888.337382047101 | 323.578256569275 | 1132.29062525061 |
| pBAD24 | N/A | N/A | N/A |
| kynUPA + KYN | 714.836947659654 | 335.676254377289 | 1067.28193750167 |
| kynUPA | N/A | N/A | N/A |
| kynUBC + KYN | 706.939249244834 | 196.859953487129 | 1183.61998449571 |
| kynUBC | N/A | N/A | N/A |
| kynUCG + KYN | 14.2034035927183 | 403.523510385201 | 950.413964553984 |
| kynUCG | N/A | N/A | N/A |
| kynURP + KYN | 443.081252185303 | 313.074955224679 | 1106.99457349836 |
| kynURP | N/A | N/A | N/A |
| kynUSM + KYN | 707.806832927905 | 368.971691304232 | 1320.01938036301 |
| kynUSM | N/A | N/A | N/A |

Anthranilic acid

| Conc. (µM) | Peak area |
| --- | --- |
| 0.001 | 42100.359 |
| 0.05 | 96018.563 |
| 0.1 | 34310.125 |
| 0.5 | 136547.078 |
| 1 | 253402.188 |
| 5 | 1206957.375 |
| 10 | 1483431.375 |
| 50 | 8058553.5 |
| 100 | 15101181 |

| Batch |  | RT | Peak | Conc. (µM) | From dilution (µM) |
| --- | --- | --- | --- | --- | --- |
| 1st | @RP1a | 8.13 | 18886648 | 123.824569742341 | 5.55360914714251 |
| 1st | @RP2a |  |  |  |  |
| 1st | @SM1a | 8.11 | 8119911 | 52.7471464691475 |  |
| 1st | @SM2a |  |  |  |  |
| 1st | @RP1b | 8.1 | 1632899.5 | 9.92265264492108 |  |
| 1st | @SM1b | 8.1 | 840390.063 | 4.6908420507133 |  |
| 1st | @RP2b | 8.09 | 77310.219 | -0.346686874088157 |  |
| 1st | @SM2b |  |  |  |  |
| 1st | @PA1a | 8.1 | 21393820 | 140.375854078783 | 6.04965280430196 |
| 1st | @PO1a | 8.1 | 19064.676 | -0.731199202529724 |  |
| 1st | @PO2a | 8.09 | 18912.617 | -0.732203031443302 |  |
| 1st | @PA2a | 8.09 | 15695.033 | -0.753444153975138 |  |
| 1st | @PO2b |  |  |  |  |
| 1st | @PA2b |  |  |  |  |
| 1st | @PO1b | 7.9 | 31861.762 | -0.646718277781079 |  |
| 1st | @PA1b | 8.26 | 753436.188 | 4.11680951154946 |  |
| 1st | @BC1a | 8.28 | 22479896 | 147.545666396002 | 6.19489570925909 |
| 1st | @BC2a | 7.9 | 221162.016 | 0.602961572231134 |  |
| 1st | @CG2a | 7.89 | 247228.484 | 0.775041319258775 |  |
| 1st | @CG1a |  |  |  |  |
| 1st | @BC1b | 8.24 | 1273206.625 | 7.5481131047868 |  |
| 1st | @BC2b |  |  |  |  |
| 1st | @CG2b |  |  |  |  |
| 1st | @CG1b |  |  |  |  |
| 1st | @BD1a |  |  |  |  |
| 1st | @BD2a |  |  |  |  |
| 1st | @BD2b |  |  |  |  |
| 1st | @BD1b |  |  |  |  |
| 1st | @M1 | 7.9 | 44974.512 | -0.560153473418758 |  |
| 1st | @M2 | 7.94 | 32999.355 | -0.639208372117587 |  |
| 1st | @HS2a |  |  |  |  |
| 1st | @PF1a |  |  |  |  |
| 1st | @PF2b |  |  |  |  |
| 1st | @HS1b | 7.78 | 5157.201 | -0.823010443691865 |  |
| 1st | @HS2b | 7.78 | 4008.36 | -0.830594603872484 |  |
| 1st | @HS1a |  |  |  |  |
| 1st | @PF2a |  |  |  |  |
| 1st | @PF1b |  |  |  |  |
|  |  |  |  |  |  |
| 2nd | @HS1a |  |  |  |  |
| 2nd | @PF2a |  |  |  |  |
| 2nd | @PF1a |  |  |  |  |
| 2nd | @HS2a |  |  |  |  |
| 2nd | @HS2b |  |  |  |  |
| 2nd | @PF1b |  |  |  |  |
| 2nd | @HS1b |  |  |  |  |
| 2nd | @PF2b |  |  |  |  |
| 2nd | @M1 |  |  |  |  |
| 2nd | @CG1a |  |  |  |  |
| 2nd | @RP1a | 9.19 | 26776898 | 175.912647957803 | 14.8983997121713 |
| 2nd | @RP2a |  |  |  |  |
| 2nd | @CG2a |  |  |  |  |
| 2nd | @CG2b |  |  |  |  |
| 2nd | @RP1b | 9.15 | 2331818.25 | 14.5366172868847 |  |
| 2nd | @CG1b | 9.15 | 64138.613 | -0.433640220756673 |  |
| 2nd | @RP2b |  |  |  |  |
| 2nd | @M2 |  |  |  |  |
| 2nd | @PA2a |  |  |  |  |
| 2nd | @PA1a | 9.14 | 25160124 | 165.239392919151 | 6.56546966906304 |
| 2nd | @SM1a | 9.13 | 10854007 | 70.7964866417127 |  |
| 2nd | @SM2a |  |  |  |  |
| 2nd | @PA2b |  |  |  |  |
| 2nd | @SM1b | 9.1 | 991470 | 5.68820760633487 |  |
| 2nd | @PA1b | 9.07 | 2009543.5 | 12.4090963103797 |  |
| 2nd | @SM2b |  |  |  |  |
| 2nd | @PO2a |  |  |  |  |
| 2nd | @PO1a | 9.07 | 45336056 | 298.432323952495 |  |
| 2nd | @BC2a |  |  |  |  |
| 2nd | @BC1a | 9.07 | 44882628 | 295.438984941807 | 17.7961920794302 |
| 2nd | @BC2b |  |  |  |  |
| 2nd | @PO2b |  |  |  |  |
| 2nd | @BC1b | 9.03 | 2757582 | 17.3473286726213 |  |
| 2nd | @PO1b | 9.03 | 1867507.5 | 11.4714349843873 |  |
| 2nd | @BD2a |  |  |  |  |
| 2nd | @BD1a |  |  |  |  |
| 2nd | @BD2b |  |  |  |  |
| 2nd | @BD1b |  |  |  |  |
|  |  |  |  |  |  |
| 3rd | @M1 |  |  |  |  |
| 3rd | @M2 |  |  |  |  |
| 3rd | @PA-1a | 8.87 | 1516963.375 | 9.1572916047769 |  |
| 3rd | @SM-1a | 8.84 | 941177.5 | 5.35619788881627 |  |
| 3rd | @CG-1a |  |  |  |  |
| 3rd | @BC-1a | 8.81 | 2389352.25 | 14.9164323107493 |  |
| 3rd | @BD-1a | 8.45 | 2503.054 | -0.840531994533896 |  |
| 3rd | @RP-1a | 8.76 | 2230938 | 13.8706487367886 |  |
| 3rd | @SM-2a |  |  |  |  |
| 3rd | @BD-2a |  |  |  |  |
| 3rd | @PA-2a |  |  |  |  |
| 3rd | @BC-2a |  |  |  |  |
| 3rd | @CG-2a |  |  |  |  |
| 3rd | @RP-2a |  |  |  |  |
| 3rd | @CG-2b |  |  |  |  |
| 3rd | @PA-1b |  |  |  |  |
| 3rd | @SM-2b |  |  |  |  |
| 3rd | @BC-2a |  |  |  |  |
| 3rd | @RP-1b |  |  |  |  |
| 3rd | @SM-1b |  |  |  |  |
| 3rd | @BD-2b |  |  |  |  |
| 3rd | @BD-1b |  |  |  |  |
| 3rd | @BC-1b |  |  |  |  |
| 3rd | @PA-2b |  |  |  |  |
| 3rd | @CG-1b |  |  |  |  |
| 3rd | @RP-2b |  |  |  |  |
|  |  |  |  |  |  |
| Dilution | @DSM3 | 8.57 | 134472.938 | 0.0306771103585315 |  |
| Dilution | @DPA3 | 8.56 | 265757.438 | 0.897361601278065 |  |
| Dilution | @DBC3 | 8.56 | 407078.469 | 1.83030300569716 |  |
| Dilution | @DBD3 |  |  |  |  |
| Dilution | @DRP3 | 8.56 | 343922.688 | 1.4133753721638 |  |
| Dilution | @DBD2 |  |  |  |  |
| Dilution | @DBC1 | 8.56 | 185757.313 | 0.36923476521498 |  |
| Dilution | @DRP1 | 8.55 | 213951.516 | 0.555360914714251 | 5.55360914714251 |
| Dilution | @DSM1 | 8.55 | 99159.141 | -0.202449573868325 |  |
| Dilution | @DPA1 | 8.54 | 169618.328 | 0.262692043121489 |  |
| Dilution | @DBD1 |  |  |  |  |
| Dilution | @DSM2 | 8.54 | 114588.93 | -0.100588662454862 |  |
| Dilution | @DBC2 | 8.52 | 399400.938 | 1.77961920794302 | 17.7961920794302 |
| Dilution | @DRP2 | 8.52 | 355505.469 | 1.48983997121713 | 14.8983997121713 |
| Dilution | @DPA2 | 8.51 | 229279.078 | 0.656546966906304 | 6.56546966906304 |
|  |  |  |  |  |  |
| Dilution (70%) | @DBC-1 | 7.97 | 786703.625 | 4.33642699648136 | 6.19489570925909 |
| Dilution (70%) | @DPA-1 | 7.97 | 771302.75 | 4.23475696301137 | 6.04965280430196 |

Summary

|  | 1^st^ (µM) | | 2^nd^ (µM) | | 3^rd^ (µM) | |
| --- | --- | --- | --- | --- | --- | --- |
|  | intra | extra | intra | extra | intra | extra |
| Media + KYN | N/A | N/A | N/A | N/A | N/A | N/A |
| Media | N/A | N/A | N/A | N/A | N/A | N/A |
| pBAD24 + KYN | N/A | N/A | N/A | N/A | N/A | N/A |
| pBAD24 | N/A | N/A | N/A | N/A | N/A | N/A |
| kynUPA + KYN | 4.11680951154946 | 6.04965280430196 | 12.4090963103797 | 6.56546966906304 | N/A | 9.1572916047769 |
| kynUPA | N/A | N/A | N/A | N/A | N/A | N/A |
| kynUBC + KYN | 7.5481131047868 | 6.19489570925909 | 17.3473286726213 | 17.7961920794302 | N/A | 14.9164323107493 |
| kynUBC | N/A | 0.602961572231134 | N/A | N/A | N/A | N/A |
| kynUCG + KYN | N/A | N/A | N/A | N/A | N/A | N/A |
| kynUCG | N/A | 0.775041319258775 | N/A | N/A | N/A | N/A |
| kynURP + KYN | 9.92265264492108 | 5.55360914714251 | 14.5366172868847 | 14.8983997121713 | N/A | 13.8706487367886 |
| kynURP | N/A | N/A | N/A | N/A | N/A | N/A |
| kynUSM + KYN | 4.6908420507133 | 52.7471464691475 | 5.68820760633487 | 70.7964866417127 | N/A | 5.35619788881627 |
| kynUSM | N/A | N/A | N/A | N/A | N/A | N/A |

Total anthranilic acid concentrations

|  | 1st total (µM) | 2nd total (µM) | 3rd total (µM) |
| --- | --- | --- | --- |
| Media + KYN | N/A | N/A | N/A |
| Media | N/A | N/A | N/A |
| pBAD24 + KYN | N/A | N/A | N/A |
| pBAD24 | N/A | N/A | N/A |
| kynUPA + KYN | 10.1664623158514 | 18.9745659794427 | 9.1572916047769 |
| kynUPA | N/A | N/A | N/A |
| kynUBC + KYN | 13.7430088140459 | 35.1435207520515 | 14.9164323107493 |
| kynUBC | 0.602961572231134 | N/A | N/A |
| kynUCG + KYN | N/A | N/A | N/A |
| kynUCG | 0.775041319258775 | N/A | N/A |
| kynURP + KYN | 15.4762617920636 | 29.435016999056 | 13.8706487367886 |
| kynURP | N/A | N/A | N/A |
| kynUSM + KYN | 57.4379885198608 | 76.4846942480476 | 5.35619788881627 |
| kynUSM | N/A | N/A | N/A |

One trial detected AA from kynUBC and kynUCG without KYN, but both were considerably low (<1 µM).

Kynurenic acid

| Conc. (µM) | Peak area |
| --- | --- |
| 0.1 | 15122.342 |
| 0.5 | 66996.727 |
| 1 | 171523.484 |
| 5 | 1190480.375 |
| 10 | 2229409.5 |
| 50 | 8427638 |
| 100 | 28836888 |

| Batch | Sample | RT | Peak | Conc. (µM) | From dilution (µM) |
| --- | --- | --- | --- | --- | --- |
| 1st | @RP1a | 7.77 | 12073947 | 41.9967693143604 |  |
| 1st | @RP2a | 7.8 | 295929.75 | -1.24308431355273 |  |
| 1st | @SM1a | 7.76 | 17073312 | 60.3506064878042 |  |
| 1st | @SM2a | 7.79 | 310210.781 | -1.19065531154089 |  |
| 1st | @RP1b | 7.73 | 4237834 | 13.2285673377682 |  |
| 1st | @SM1b | 7.73 | 5090158 | 16.357647914005 |  |
| 1st | @RP2b | 7.73 | 454009.344 | -0.662737183723218 |  |
| 1st | @SM2b |  |  |  |  |
| 1st | @PA1a | 7.76 | 27165988 | 97.4031785541213 |  |
| 1st | @PO1a | 7.77 | 300721.344 | -1.22549325227249 |  |
| 1st | @PO2a | 7.77 | 395842.844 | -0.87627999765041 |  |
| 1st | @PA2a | 7.77 | 329782.563 | -1.11880272625813 |  |
| 1st | @PO2b |  |  |  |  |
| 1st | @PA2b |  |  |  |  |
| 1st | @PO1b | 7.88 | 133021.094 | -1.84116005844604 |  |
| 1st | @PA1b | 7.85 | 2778477.25 | 7.87092768403894 |  |
| 1st | @BC1a | 7.88 | 4968584.5 | 15.9113231860434 |  |
| 1st | @BC2a |  |  |  |  |
| 1st | @CG2a | 7.88 | 559336.813 | -0.276055431957355 |  |
| 1st | @CG1a | 7.85 | 13603818 | 47.6132832577059 |  |
| 1st | @BC1b | 7.84 | 2210889.25 | 5.78717950129962 |  |
| 1st | @BC2b |  |  |  |  |
| 1st | @CG2b |  |  |  |  |
| 1st | @CG1b | 7.83 | 7033517.5 | 23.4921747654082 |  |
| 1st | @BD1a | 7.85 | 26334338 | 94.3499970630131 |  |
| 1st | @BD2a | 7.85 | 717559.938 | 0.304818633713673 |  |
| 1st | @BD2b |  |  |  |  |
| 1st | @BD1b | 7.81 | 11698414 | 40.6180999162959 |  |
| 1st | @M1 | 7.58 | 277082.875 | -1.31227559584123 |  |
| 1st | @M2 |  |  |  |  |
| 1st | @HS2a |  |  |  |  |
| 1st | @PF1a | 8.99 | 9832129 | 33.7665315652672 |  |
| 1st | @PF2b |  |  |  |  |
| 1st | @HS1b | 8.87 | 1044025.563 | 1.50335023202197 |  |
| 1st | @HS2b |  |  |  |  |
| 1st | @HS1a | 8.88 | 7077560.5 | 23.6538669104366 |  |
| 1st | @PF2a |  |  |  |  |
| 1st | @PF1b | 8.79 | 1379446.625 | 2.73475933227602 |  |
|  |  |  |  |  |  |
| 2nd | @HS1a | 8.77 | 5963588.5 | 19.5642153839376 |  |
| 2nd | @PF2a | 8.76 | 191548.875 | -1.62629089754321 |  |
| 2nd | @PF1a | 8.76 | 6587274 | 21.8539105981174 |  |
| 2nd | @HS2a | 8.76 | 219404.125 | -1.52402776554033 |  |
| 2nd | @HS2b |  |  |  |  |
| 2nd | @PF1b | 8.72 | 695505.813 | 0.223852787200611 |  |
| 2nd | @HS1b | 8.73 | 1022774.938 | 1.42533422177188 |  |
| 2nd | @PF2b | 8.66 | 130456.781 | -1.85057425070121 |  |
| 2nd | @M1 |  |  |  |  |
| 2nd | @CG1a | 8.75 | 21378208 | 76.1548856777832 |  |
| 2nd | @RP1a | 8.73 | 3125604.75 | 9.14531385376742 |  |
| 2nd | @RP2a | 8.72 | 247606.203 | -1.42049134690221 |  |
| 2nd | @CG2a | 8.68 | 224927.031 | -1.50375188701411 |  |
| 2nd | @CG2b |  |  |  |  |
| 2nd | @RP1b | 8.66 | 545779.313 | -0.325828182592478 |  |
| 2nd | @CG1b | 8.66 | 1823980.625 | 4.3667475255885 |  |
| 2nd | @RP2b | 8.31 | 147512.078 | -1.7879602699091 |  |
| 2nd | @M2 |  |  |  |  |
| 2nd | @PA2a | 8.71 | 196308.922 | -1.60881565267192 |  |
| 2nd | @PA1a | 8.68 | 2838233.25 | 8.09030592390267 |  |
| 2nd | @SM1a | 8.68 | 9259280 | 31.6634690221302 |  |
| 2nd | @SM2a | 8.71 | 171375.141 | -1.70035338928293 |  |
| 2nd | @PA2b | 8.49 | 210022.797 | -1.55846881286988 |  |
| 2nd | @SM1b | 8.61 | 1059276.375 | 1.55933952670455 |  |
| 2nd | @PA1b | 8.58 | 689450 | 0.201620482546955 |  |
| 2nd | @SM2b | 8.52 | 36881.41 | -2.19411130446275 |  |
| 2nd | @PO2a | 8.65 | 158772.453 | -1.74662080194429 |  |
| 2nd | @PO1a | 8.62 | 15128854 | 53.212046786202 |  |
| 2nd | @BC2a | 8.65 | 106419.336 | -1.9388213283992 |  |
| 2nd | @BC1a | 8.62 | 2584912.25 | 7.16030533650528 |  |
| 2nd | @BC2b |  |  |  |  |
| 2nd | @PO2b |  |  |  |  |
| 2nd | @BC1b | 8.58 | 464128.125 | -0.625588774101649 |  |
| 2nd | @PO1b | 8.57 | 445800.125 | -0.69287514501373 |  |
| 2nd | @BD2a | 8.61 | 149988.281 | -1.7788695500536 |  |
| 2nd | @BD1a | 8.58 | 16525853 | 58.3407565678371 |  |
| 2nd | @BD2b |  |  |  |  |
| 2nd | @BD1b | 8.57 | 693489.875 | 0.216451807715465 |  |
|  |  |  |  |  |  |
| 3rd | @M1 | 8.51 | 11955.246 | -2.28562107728681 |  |
| 3rd | @M2 |  |  |  |  |
| 3rd | @PA-1a | 8.45 | 15891204 | 56.0108117831916 | 73.9067616781943 |
| 3rd | @SM-1a | 8.43 | 42059156 | 152.079478537968 | 136.682893519538 |
| 3rd | @CG-1a | 8.42 | 39366356 | 142.193580480785 | 91.7194736919394 |
| 3rd | @BC-1a | 8.39 | 11090087 | 38.3847893446114 |  |
| 3rd | @BD-1a | 8.37 | 34221764 | 123.306581053497 | 180.255352658707 |
| 3rd | @RP-1a | 8.36 | 18510804 | 65.6279755348987 |  |
| 3rd | @SM-2a | 8.36 | 94836.734 | -1.98134376697946 |  |
| 3rd | @BD-2a | 8.35 | 79038.219 | -2.03934380736303 |  |
| 3rd | @PA-2a | 8.33 | 39027.504 | -2.18623249188657 |  |
| 3rd | @BC-2a | 8.34 | 30792.955 | -2.21646344552623 |  |
| 3rd | @CG-2a | 8.32 | 108802.641 | -1.93007165881023 |  |
| 3rd | @RP-2a | 8.31 | 37427.301 | -2.1921072110372 |  |
| 3rd | @CG-2b |  |  |  |  |
| 3rd | @PA-1b | 8.35 | 7253.931 | -2.30288070326152 |  |
| 3rd | @SM-2b |  |  |  |  |
| 3rd | @BC-2a |  |  |  |  |
| 3rd | @RP-1b |  |  |  |  |
| 3rd | @SM-1b |  |  |  |  |
| 3rd | @BD-2b |  |  |  |  |
| 3rd | @BD-1b |  |  |  |  |
| 3rd | @BC-1b |  |  |  |  |
| 3rd | @PA-2b |  |  |  |  |
| 3rd | @CG-1b |  |  |  |  |
| 3rd | @RP-2b |  |  |  |  |
|  |  |  |  |  |  |
| Dilution | @DSM3 | 8.2 | 4357609 | 13.6682893519538 | 136.682893519538 |
| Dilution | @DPA3 | 8.19 | 2647662.5 | 7.39067616781943 | 73.9067616781943 |
| Dilution | @DBC3 | 8.19 | 1995889.375 |  |  |
| Dilution | @DBD3 | 8.2 | 5544470.5 | 18.0255352658707 | 180.255352658707 |

| Dilution (1:1) | @DCG-3 | 7.54 | 13126173 | 45.8597368459697 | 91.7194736919394 |
| --- | --- | --- | --- | --- | --- |

Summary

|  | 1^st^ (µM) | | 2^nd^ (µM) | | 3^rd^ (µM) | |
| --- | --- | --- | --- | --- | --- | --- |
|  | intra | extra | intra | extra | intra | extra |
| Media + KYN | N/A | N/A | N/A | N/A | N/A | N/A |
| Media | N/A | N/A | N/A | N/A | N/A | N/A |
| pBAD24 + KYN | 40.6180999162959 | 94.349997063013 | 0.216451807715465 | 58.3407565678371 | N/A | 180.255352658707 |
| pBAD24 | N/A | 0.304818633713673 | N/A | N/A | N/A | N/A |
| kynUPA + KYN | 7.87092768403894 | 97.4031785541213 | 0.201620482546955 | 8.09030592390267 | N/A | 73.9067616781943 |
| kynUPA | N/A | N/A | N/A | N/A | N/A | N/A |
| kynUBC + KYN | 5.78717950129962 | 15.9113231860434 | N/A | 7.16030533650528 | N/A | 38.3847893446114 |
| kynUBC | N/A | N/A | N/A | N/A | N/A | N/A |
| kynUCG + KYN | 23.4921747654082 | 47.6132832577059 | 4.3667475255885 | 76.1548856777832 | N/A | 91.7194736919394 |
| kynUCG | N/A | N/A | N/A | N/A | N/A | N/A |
| kynURP + KYN | 13.2285673377682 | 41.9967693143604 | N/A | 9.14531385376742 | N/A | 65.6279755348988 |
| kynURP | N/A | N/A | N/A | N/A | N/A | N/A |
| kynUSM + KYN | 16.357647914005 | 60.3506064878042 | 1.55933952670455 | 31.6634690221302 | N/A | 136.682893519538 |
| kynUSM | N/A | N/A | N/A | N/A | N/A | N/A |

Total kynurenic acid concentrations

|  | 1st total (µM) | 2nd total (µM) | 3rd total (µM) |
| --- | --- | --- | --- |
| Media + KYN | N/A | N/A | N/A |
| Media | N/A | N/A | N/A |
| pBAD24 + KYN | 134.968096979309 | 58.5572083755526 | 180.255352658707 |
| pBAD24 | 0.304818633713673 | N/A | N/A |
| kynUPA + KYN | 105.27410623816 | 8.29192640644963 | 73.9067616781943 |
| kynUPA | N/A | N/A | N/A |
| kynUBC + KYN | 21.698502687343 | 7.16030533650528 | 38.3847893446114 |
| kynUBC | N/A | N/A | N/A |
| kynUCG + KYN | 71.1054580231141 | 80.5216332033717 | 91.7194736919394 |
| kynUCG | N/A | N/A | N/A |
| kynURP + KYN | 55.2253366521286 | 9.14531385376742 | 65.6279755348988 |
| kynURP | N/A | N/A | N/A |
| kynUSM + KYN | 76.7082544018092 | 33.2228085488348 | 136.682893519538 |
| kynUSM | N/A | N/A | N/A |

One trial of pBAD24 without KYN was detected KYNA concentrations, but it was considerably low (< 1 µM).

Picolinic acid

| Conc. (µM) | Peak area |
| --- | --- |
| 0.1 | 35935.078 |
| 0.5 | 59970.199 |
| 1 | 628371 |
| 5 | 1112103.875 |
| 10 | 1348235.625 |
| 50 | 3224397 |
| 100 | 5626997 |

| Batch | Sample | RT | Peak | Conc. (µM) |
| --- | --- | --- | --- | --- |
| 1st | @RP1a |  |  |  |
| 1st | @RP2a |  |  |  |
| 1st | @SM1a |  |  |  |
| 1st | @SM2a |  |  |  |
| 1st | @RP1b | 2.73 | 380611.156 | -1.56436435973704 |
| 1st | @SM1b | 2.73 | 357507.156 | -2.00208104882254 |
| 1st | @RP2b | 2.74 | 378887.094 | -1.5970275656935 |
| 1st | @SM2b | 2.72 | 348858.469 | -2.16593469488282 |
| 1st | @PA1a |  |  |  |
| 1st | @PO1a |  |  |  |
| 1st | @PO2a |  |  |  |
| 1st | @PA2a |  |  |  |
| 1st | @PO2b | 2.69 | 409321.469 | -1.02043330238903 |
| 1st | @PA2b | 2.69 | 334998.969 | -2.42850976640206 |
| 1st | @PO1b | 2.89 | 329914.219 | -2.5248428660743 |
| 1st | @PA1b | 2.86 | 191736.328 | -5.14269124528731 |
| 1st | @BC1a |  |  |  |
| 1st | @BC2a |  |  |  |
| 1st | @CG2a |  |  |  |
| 1st | @CG1a |  |  |  |
| 1st | @BC1b | 2.88 | 199404.531 | -4.99741335278404 |
| 1st | @BC2b | 2.82 | 296050.219 | -3.16641306860163 |
| 1st | @CG2b | 2.84 | 306259.25 | -2.97299793494117 |
| 1st | @CG1b | 2.84 | 273701.25 | -3.58982532254703 |
| 1st | @BD1a |  |  |  |
| 1st | @BD2a |  |  |  |
| 1st | @BD2b | 2.85 | 306300.281 | -2.97222058238448 |
| 1st | @BD1b | 2.84 | 282519.281 | -3.42276337078226 |
| 1st | @M1 |  |  |  |
| 1st | @M2 |  |  |  |
| 1st | @HS2a |  |  |  |
| 1st | @PF1a |  |  |  |
| 1st | @PF2b | 3.4 | 11852.785 | -8.55067379648751 |
| 1st | @HS1b | 3.38 | 11257.427 | -8.5619531477938 |
| 1st | @HS2b | 3.38 | 11823.896 | -8.55122111285831 |
| 1st | @HS1a |  |  |  |
| 1st | @PF2a |  |  |  |
| 1st | @PF1b | 3.32 | 18165.939 | -8.43106797643181 |
|  |  |  |  |  |
| 2nd | @HS1a |  |  |  |
| 2nd | @PF2a |  |  |  |
| 2nd | @PF1a |  |  |  |
| 2nd | @HS2a |  |  |  |
| 2nd | @HS2b | 3.24 | 12212.837 | -8.54385243354868 |
| 2nd | @PF1b | 3.26 | 15422.807 | -8.4830379667696 |
| 2nd | @HS1b | 3.22 | 14021.45 | -8.50958736714472 |
| 2nd | @PF2b | 3.24 | 14826.004 | -8.49434469431446 |
| 2nd | @M1 |  |  |  |
| 2nd | @CG1a |  |  |  |
| 2nd | @RP1a |  |  |  |
| 2nd | @RP2a |  |  |  |
| 2nd | @CG2a |  |  |  |
| 2nd | @CG2b | 3.22 | 18252.404 | -8.42942985430915 |
| 2nd | @RP1b | 3.23 | 17950.223 | -8.43515482257545 |
| 2nd | @CG1b | 3.19 | 17635.512 | -8.44111717787924 |
| 2nd | @RP2b | 3.19 | 17631.063 | -8.44120146638122 |
| 2nd | @M2 |  |  |  |
| 2nd | @PA2a |  |  |  |
| 2nd | @PA1a |  |  |  |
| 2nd | @SM1a |  |  |  |
| 2nd | @SM2a |  |  |  |
| 2nd | @PA2a | 3.16 | 11895.521 | -8.54986414186386 |
| 2nd | @SM1b | 3.17 | 15490.985 | -8.48174630089233 |
| 2nd | @PA1b | 3.16 | 16869.744 | -8.45562503078643 |
| 2nd | @SM2b | 3.13 | 9099.727 | -8.6028318397969 |
| 2nd | @PO2a |  |  |  |
| 2nd | @PO1a |  |  |  |
| 2nd | @BC2a |  |  |  |
| 2nd | @BC1a |  |  |  |
| 2nd | @BC2b | 3.13 | 9785.157 | -8.58984602997177 |
| 2nd | @PO2b | 3.13 | 11773.246 | -8.55218070212 |
| 2nd | @BC1b | 3.13 | 13107.715 | -8.52689852793513 |
| 2nd | @PO1b | 3.15 | 9488.661 | -8.59546329310573 |
| 2nd | @BD2a |  |  |  |
| 2nd | @BD1a |  |  |  |
| 2nd | @BD2b | 3.11 | 7738.804 | -8.62861519807514 |
| 2nd | @BD1b | 3.11 | 7644.729 | -8.63039749540572 |
|  |  |  |  |  |
| 3rd | @M1 |  |  |  |
| 3rd | @M2 |  |  |  |
| 3rd | @PA-1a | 3.66 | 6218.187 | -8.65742403804255 |
| 3rd | @SM-1a | 3.64 | 7366.114 | -8.63567599416479 |
| 3rd | @CG-1a | 3.63 | 6360.441 | -8.65472896576549 |
| 3rd | @BC-1a | 3.61 | 8592.076 | -8.61244953867723 |
| 3rd | @BD-1a | 3.61 | 16768.65 | -8.45754030653809 |
| 3rd | @RP-1a | 3.59 | 8868.177 | -8.6072186688896 |
| 3rd | @SM-2a | 3.59 | 7060.714 | -8.64146194797567 |
| 3rd | @BD-2a |  |  |  |
| 3rd | @PA-2a | 3.55 | 9118.137 | -8.60248305325578 |
| 3rd | @BC-2a | 3.55 | 7423.754 | -8.63458397590133 |
| 3rd | @CG-2a | 3.54 | 9742.748 | -8.59064948941894 |
| 3rd | @RP-2a | 3.53 | 10843.64 | -8.56979254684273 |
| 3rd | @CG-2b |  |  |  |
| 3rd | @PA-1b |  |  |  |
| 3rd | @SM-2b |  |  |  |
| 3rd | @BC-2a |  |  |  |
| 3rd | @RP-1b |  |  |  |
| 3rd | @SM-1b |  |  |  |
| 3rd | @BD-2b |  |  |  |
| 3rd | @BD-1b |  |  |  |
| 3rd | @BC-1b |  |  |  |
| 3rd | @PA-2b |  |  |  |
| 3rd | @CG-1b |  |  |  |
| 3rd | @RP-2b |  |  |  |

Picolinic acid from all conditions was lower than the detection limit (< 0.1 µM).

Quinolinic acid

| Conc. (µM) | Peak area |
| --- | --- |
| 0.1 | 16543.262 |
| 0.5 | 35935.078 |
| 1 | 59970.199 |
| 5 | 628371 |
| 10 | 652422.563 |
| 50 | 3224397 |
| 100 | 5626997 |

| Batch | Sample | RT | Peak | Conc. (µM) |
| --- | --- | --- | --- | --- |
| 1st | @RP1a |  |  |  |
| 1st | @RP2a |  |  |  |
| 1st | @SM1a |  |  |  |
| 1st | @SM2a |  |  |  |
| 1st | @RP1b |  |  |  |
| 1st | @SM1b |  |  |  |
| 1st | @RP2b |  |  |  |
| 1st | @SM2b |  |  |  |
| 1st | @PA1a |  |  |  |
| 1st | @PO1a |  |  |  |
| 1st | @PO2a |  |  |  |
| 1st | @PA2a |  |  |  |
| 1st | @PO2b |  |  |  |
| 1st | @PA2b |  |  |  |
| 1st | @PO1b |  |  |  |
| 1st | @PA1b |  |  |  |
| 1st | @BC1a | 3.15 | 15287.383 | -1.80698099229083 |
| 1st | @BC2a | 3.16 | 30191.793 | -1.5434473265436 |
| 1st | @CG2a | 3.16 | 28924.666 | -1.56585214654502 |
| 1st | @CG1a | 3.15 | 16838.861 | -1.77954839451163 |
| 1st | @BC1b |  |  |  |
| 1st | @BC2b |  |  |  |
| 1st | @CG2b |  |  |  |
| 1st | @CG1b |  |  |  |
| 1st | @BD1a | 3.15 | 27132.211 | -1.59754560082043 |
| 1st | @BD2a | 3.13 | 35852.086 | -1.44336434684207 |
| 1st | @BD2b |  |  |  |
| 1st | @BD1b |  |  |  |
| 1st | @M1 |  |  |  |
| 1st | @M2 |  |  |  |
| 1st | @HS2a |  |  |  |
| 1st | @PF1a |  |  |  |
| 1st | @PF2b |  |  |  |
| 1st | @HS1b |  |  |  |
| 1st | @HS2b |  |  |  |
| 1st | @HS1a |  |  |  |
| 1st | @PF2a |  |  |  |
| 1st | @PF1b |  |  |  |
|  |  |  |  |  |
| 2nd | @HS1a |  |  |  |
| 2nd | @PF2a |  |  |  |
| 2nd | @PF1a |  |  |  |
| 2nd | @HS2a |  |  |  |
| 2nd | @HS2b |  |  |  |
| 2nd | @PF1b |  |  |  |
| 2nd | @HS1b |  |  |  |
| 2nd | @PF2b |  |  |  |
| 2nd | @M1 |  |  |  |
| 2nd | @CG1a |  |  |  |
| 2nd | @RP1a |  |  |  |
| 2nd | @RP2a |  |  |  |
| 2nd | @CG2a |  |  |  |
| 2nd | @CG2b |  |  |  |
| 2nd | @RP1b |  |  |  |
| 2nd | @CG1b |  |  |  |
| 2nd | @RP2b |  |  |  |
| 2nd | @M2 |  |  |  |
| 2nd | @PA2a |  |  |  |
| 2nd | @PA1a |  |  |  |
| 2nd | @SM1a |  |  |  |
| 2nd | @SM2a |  |  |  |
| 2nd | @PA2a |  |  |  |
| 2nd | @SM1b |  |  |  |
| 2nd | @PA1b |  |  |  |
| 2nd | @SM2b |  |  |  |
| 2nd | @PO2a |  |  |  |
| 2nd | @PO1a |  |  |  |
| 2nd | @BC2a |  |  |  |
| 2nd | @BC1a |  |  |  |
| 2nd | @BC2b |  |  |  |
| 2nd | @PO2b |  |  |  |
| 2nd | @BC1b |  |  |  |
| 2nd | @PO1b |  |  |  |
| 2nd | @BD2a |  |  |  |
| 2nd | @BD1a |  |  |  |
| 2nd | @BD2b |  |  |  |
| 2nd | @BD1b |  |  |  |
|  |  |  |  |  |
| 3rd | @M1 |  |  |  |
| 3rd | @M2 |  |  |  |
| 3rd | @PA-1a |  |  |  |
| 3rd | @SM-1a |  |  |  |
| 3rd | @CG-1a |  |  |  |
| 3rd | @BC-1a |  |  |  |
| 3rd | @BD-1a |  |  |  |
| 3rd | @RP-1a |  |  |  |
| 3rd | @SM-2a |  |  |  |
| 3rd | @BD-2a |  |  |  |
| 3rd | @PA-2a |  |  |  |
| 3rd | @BC-2a |  |  |  |
| 3rd | @CG-2a |  |  |  |
| 3rd | @RP-2a |  |  |  |
| 3rd | @CG-2b |  |  |  |
| 3rd | @PA-1b |  |  |  |
| 3rd | @SM-2b |  |  |  |
| 3rd | @BC-2a |  |  |  |
| 3rd | @RP-1b |  |  |  |
| 3rd | @SM-1b |  |  |  |
| 3rd | @BD-2b |  |  |  |
| 3rd | @BD-1b |  |  |  |
| 3rd | @BC-1b |  |  |  |
| 3rd | @PA-2b |  |  |  |
| 3rd | @CG-1b |  |  |  |
| 3rd | @RP-2b |  |  |  |

Quinolinic acid from all conditions was lower than the detection limit (< 0.1 µM).
